# Supplementary material for: Repeated ketamine anesthesia during neurodevelopment upregulates hippocampal activity and enhances drug reward in male mice
Source: Commun Biol. 2022 Jul 15;5:709. doi: 10.1038/s42003-022-03667-4 (PMC9287305; doi:10.1038/s42003-022-03667-4)
Supplement: Supplementary file 2 — Supplementary Information [file 42003_2022_3667_MOESM2_ESM.pdf]

Supplementary information

Repeated ketamine anesthesia during neurodevelopment upregulates hippocampal activity and enhances drug reward in male mice

File contains:

1. Supplementary Figures
2. Supplementary Tables
3. Supplementary Notes

Supplementary figures

(Supplementary Figure 1-10)

Supplementary Figure 1

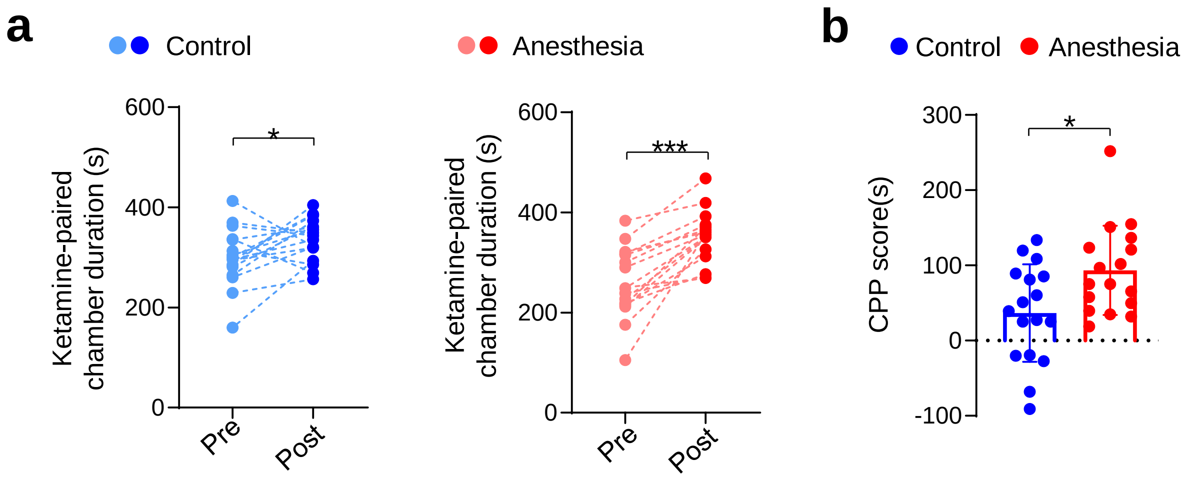

Supplementary Figure 1: Conditioned place preference (CPP) test was repeated to confirm enhanced place preference to low-dose ketamine in young mice that received early ketamine anesthesia. Place preference to low-dose ketamine (20 mg·kg^{-1}, i.p.) was measured 1 week after ketamine anesthesia injections. **a** Time spent in the ketamine-paired chamber was significantly increased after conditioning in both groups (Control, $p = 0.034$; paired t-test; n = 17 mice; Anesthesia, $p < 0.001$; paired t-test; n = 17 mice). **b** Summary graph comparing ketamine-CPP scores between groups ($p = 0.012$, Student's t-test; Control, n = 17 mice; Anesthesia, n = 17 mice). Values are presented as means ± SD (*$p < 0.05$, ***$p < 0.001$).

Supplementary Figure 2

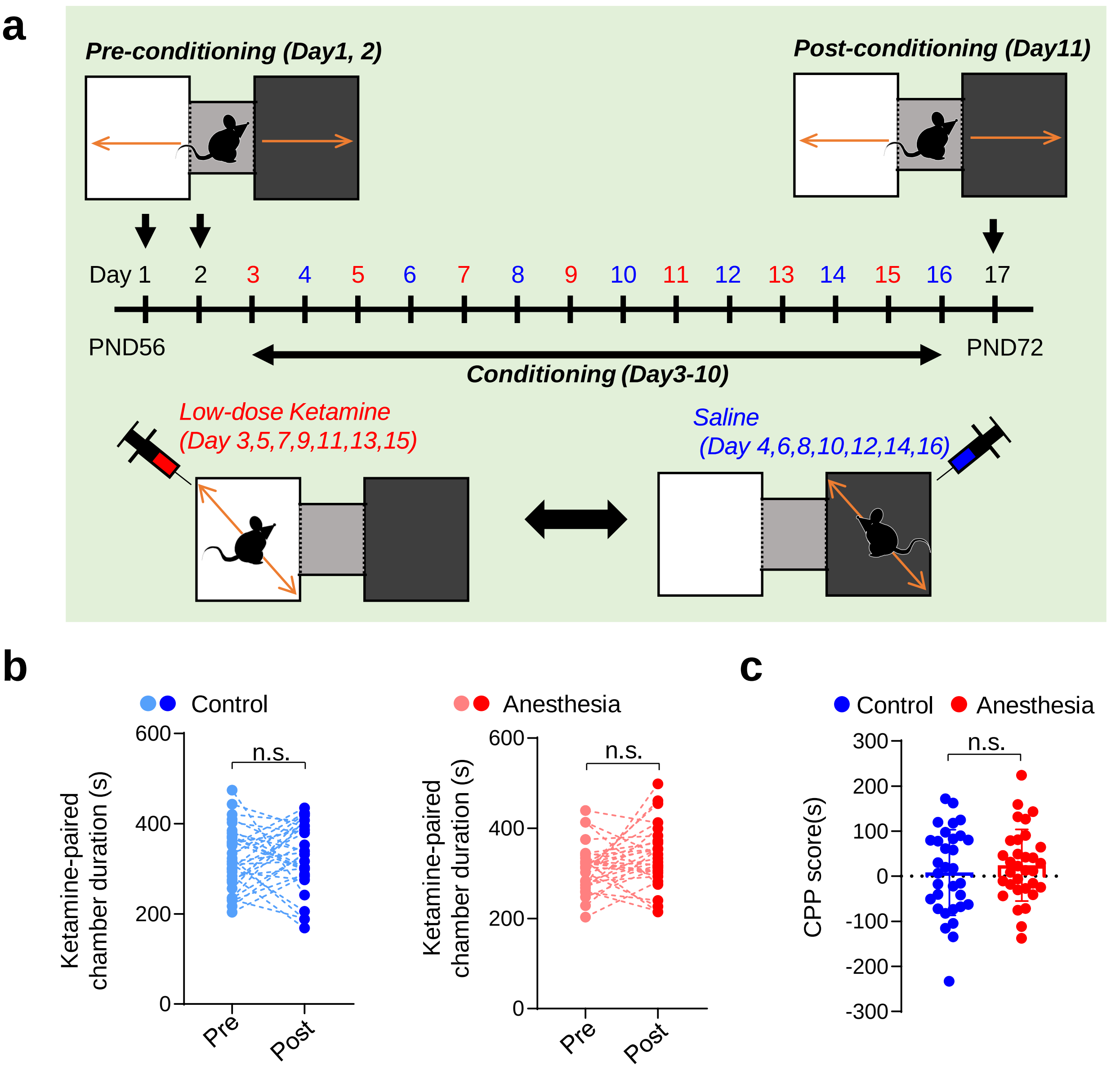

Supplementary Figure 2: Place preference to low-dose ketamine does not develop after a 14-day conditioning period in adult male mice.

a Experimental scheme of conditioned place preference test with low dose ketamine in adult mice (14-day conditioning). **b** Place preference to low-dose ketamine did not develop in both groups (paired t-test, Control p = 0.620; Anesthesia p = 0.091; Control n = 32 mice, Anesthesia n = 32 mice). **c** Summary bar graph of ketamine-CPP scores in adult male mice (Student's t-test, p = 0.465; Control n = 32 mice, Anesthesia n = 32 mice). Values are presented as means ± SD. n.s. = not significant.

Supplementary Figure 3

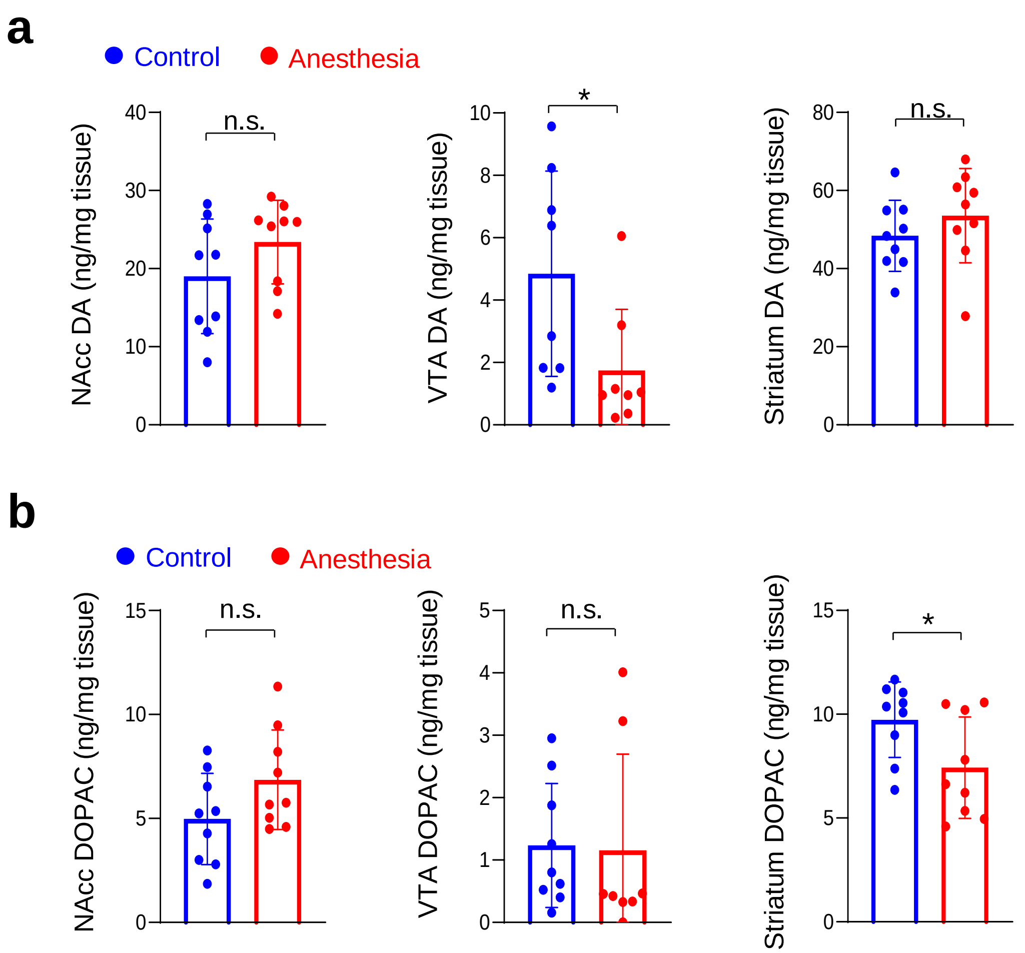

Supplementary Figure 3: The level of dopamine and its metabolite (DOPAC) in the NAcc, VTA, and striatum was not increased after the ketamine-CPP test.

The level of dopamine (DA) and its metabolite, 3,4-dihydroxyphenylacetic acid (DOPAC), were evaluated in the nucleus accumbens (NAcc), ventral tegmental area (VTA), and striatum by high-performance liquid chromatography (HPLC). HPLC was performed 1 day after the ketamine-CPP test at postnatal day 39 in male. **a, b** n = 9 mice per group (VTA DA n = 8 mice per group; VTA DOPAC, Control n = 9, Anesthesia n = 8) (Kruskal Wallis test & Student's t-test). Values are presented as means ± SD. n.s. = not significant; *p < 0.05.

Supplementary Figure 4

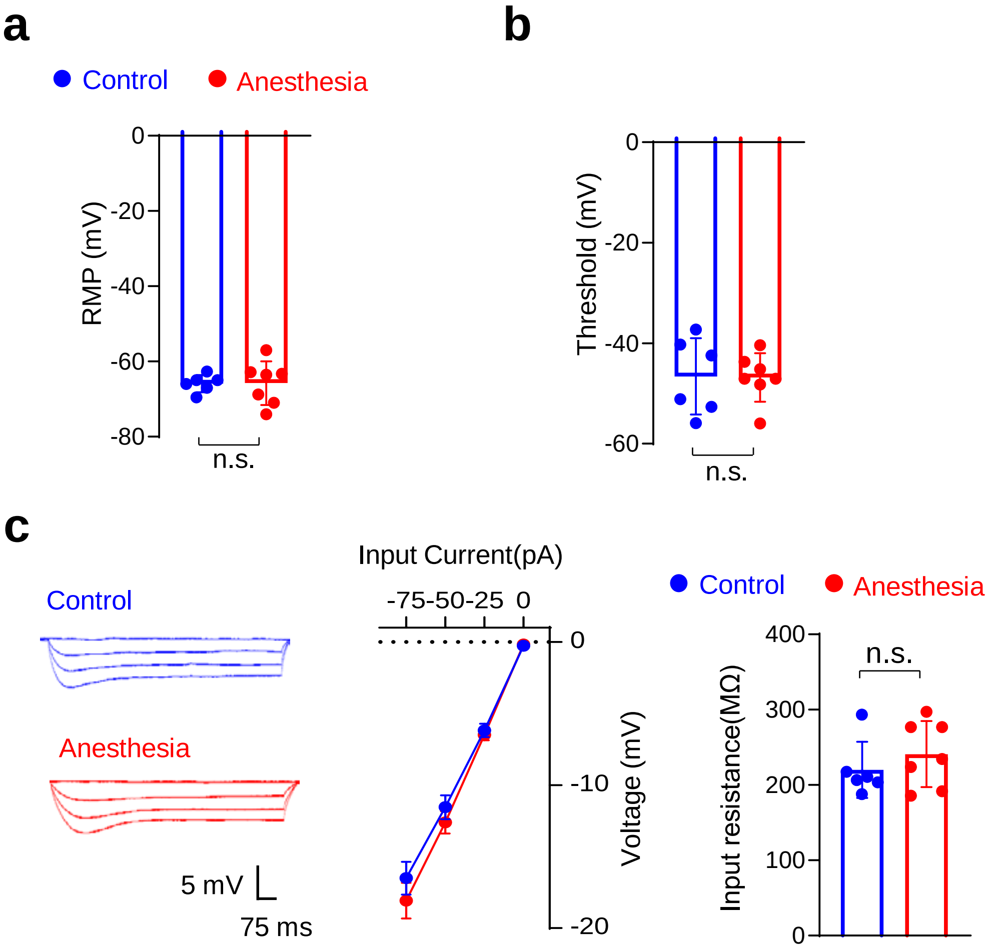

Supplementary Figure 4: Repeated ketamine anesthesia does not affect resting membrane potential (RMP), action potential firing thresholds, and input resistance in male mice.

Repeated ketamine anesthesia did not affect **a** Resting membrane potential (RMP) (Student's t-test, $p = 0.968$), **b** Action potential threshold (Student's t-test, $p = 0.959$), and **c** Input resistance (Student's t-test, $p = 0.377$). Control, n = 6 mice [total 20 cells]; Anesthesia, n = 7 mice [total 20 cells]. Values are presented as means ± SD. n.s. = not significant.

Supplementary Figure 5

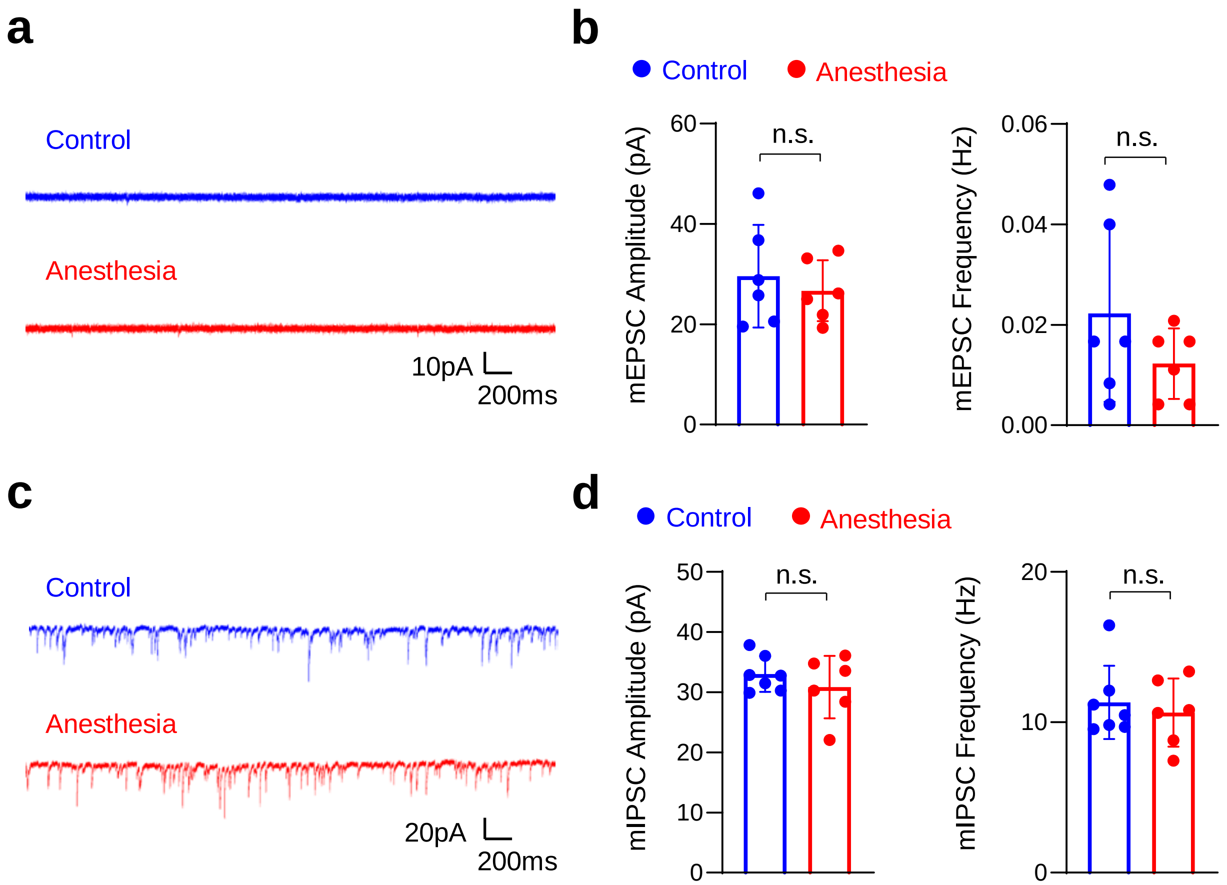

Supplementary Figure 5: Repeated ketamine anesthesia does not affect miniature excitatory/ inhibitory synaptic transmission (mEPSC/mIPSC) in hippocampal CA1 pyramidal neurons male mice. mEPSCs/mIPSCs were measured 1 week after repeated saline injections (14 ml/kg, control group) or ketamine anesthesia (35 mg/kg, Anesthesia group). **a** Example of mEPSCs **b** Repeated ketamine anesthesia did not affect the amplitude (Student's t-test, p = 0.564) and frequency (Student's t-test, p = 0.225) of mEPSCs (Control n = 6 mice [total 21 cells]; Anesthesia n = 6 mice [total 21 cells]). **c** Example of mIPSCs **d** Repeated ketamine anesthesia did not affect the amplitude (Student's t-test, p = 0.364) and frequency (Student's t-test, p = 0.613) of mIPSCs (Control n = 7 mice [total 23 cells]; Anesthesia n= 6 mice [total 22 cells]). Values are presented as means ± SD. n.s. = not significant.

Supplementary Figure 6

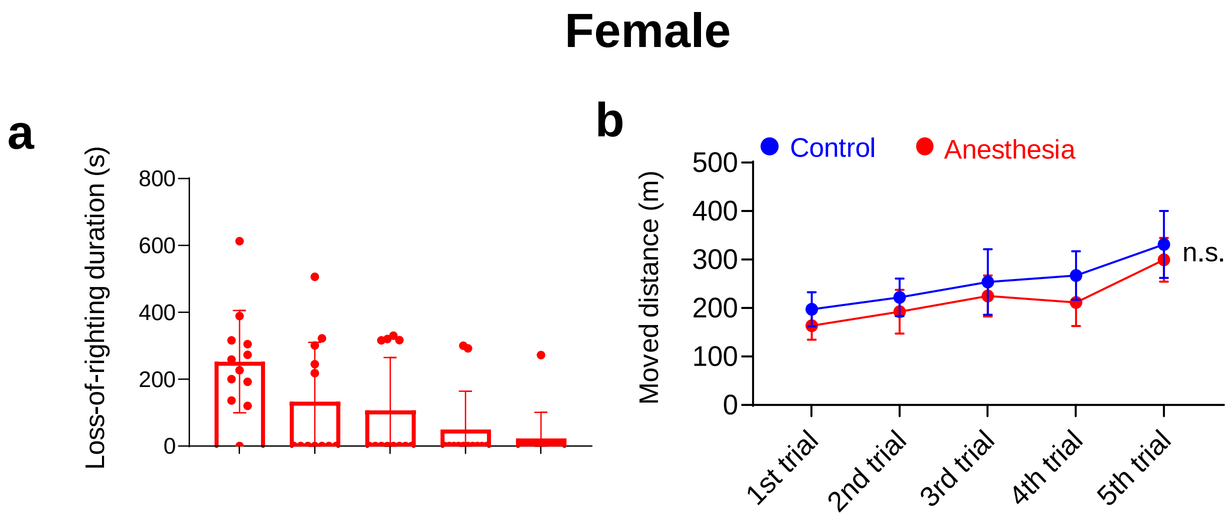

Supplementary Figure 6: Early repeated ketamine exposures induce tolerance in female mice but do not affect behavioral sensitization.

a PND16 mice were injected with ketamine for 5 consecutive days. The duration of ketamine anesthesia, measured as the LOR reflex duration, was significantly reduced by repeated injections (acute tolerance) (p = 0.006, RM-ANOVA; n = 12). **b** Early repeated ketamine anesthesia does not affect low-dose ketamine-induced behavioral sensitization in female mice (p = 0.700, RM-ANOVA; n = 12 mice/group).Values are presented as means ± SD. n.s. = not significant.

Supplementary Figure 7

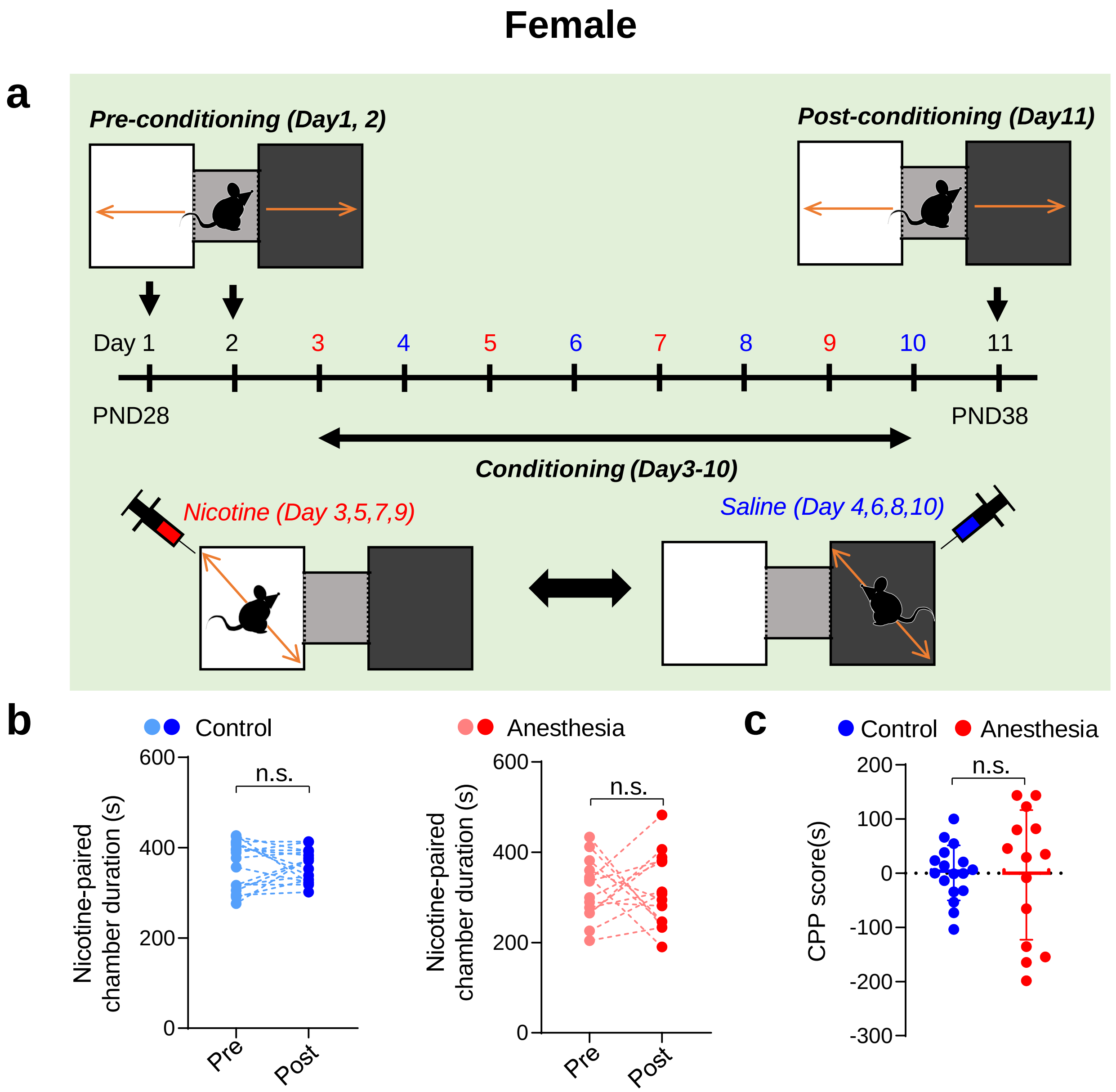

Supplementary Figure 7: Repeated subcutaneous nicotine injections doe not induce place preference in late postnatal female mice.

a Early repeated ketamine anesthesia does not affect nicotine-CPP in female mice (0.5 mg/kg, s.c.) **b** The time spent in the nicotine-paired (white) chamber was not increased after conditioning in either the Control (p = 0.946) or Anesthesia (p = 0.921) group (paired t-test; Control, n = 17; Anesthesia, n = 14). **c** Summary bar graph showing nicotine-CPP scores in female mice (p = 0.912, Welch ANOVA; Control, n = 17 mice; Anesthesia, n = 14 mice). Values are presented as means ± SD (n.s., not significant).

Supplementary Figure 8

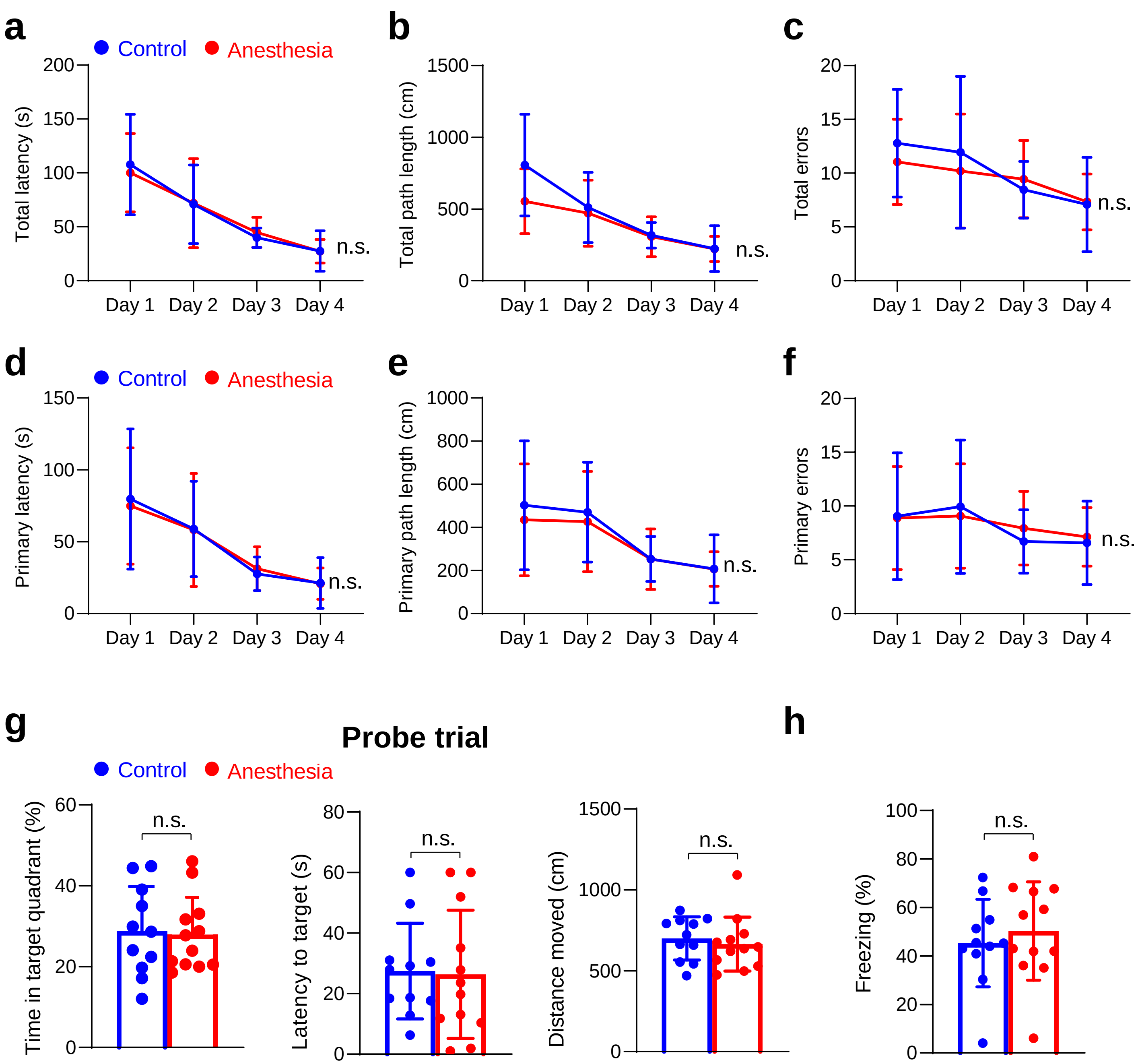

Supplementary Figure 8: Early repeated ketamine anesthesia in female mice did not affect non-drug associated contextual learning and memory in the Barnes maze and fear chamber test.

a-g Early repeated ketamine anesthesia did not affect contextual learning and memory in the Barnes maze test (Control n = 11 mice, Anesthesia n = 12 mice). **a-c** Total latency (p = 0.244, RM-ANOVA), total path length (p = 0.723, RM-ANOVA), and total number of errors (p = 0.442, RM-ANOVA) for mice to enter the escape box were comparable between groups. **d-f** Primary latency (p = 0.900, RM-ANOVA), primary path length (p = 0.736, RM-ANOVA), and primary errors (p = 0.902, RM-ANOVA) to the first encounter of the escape hole were comparable between groups. **g** The results of time in target quadrant (p = 0.834, Student's t-test), latency to target (p = 0.666, Kruskal-Wallis test) and distance moved (p = 0.591, Student's t-test) during the probe trial were comparable between groups. **h** Early repeated ketamine anesthesia did not affect contextual fear memory (p = 0.542, Student's t-test, control n = 11 mice, anesthesia n = 12 mice). Values are presented as means ± SD (n.s., not significant).

Supplementary Figure 9

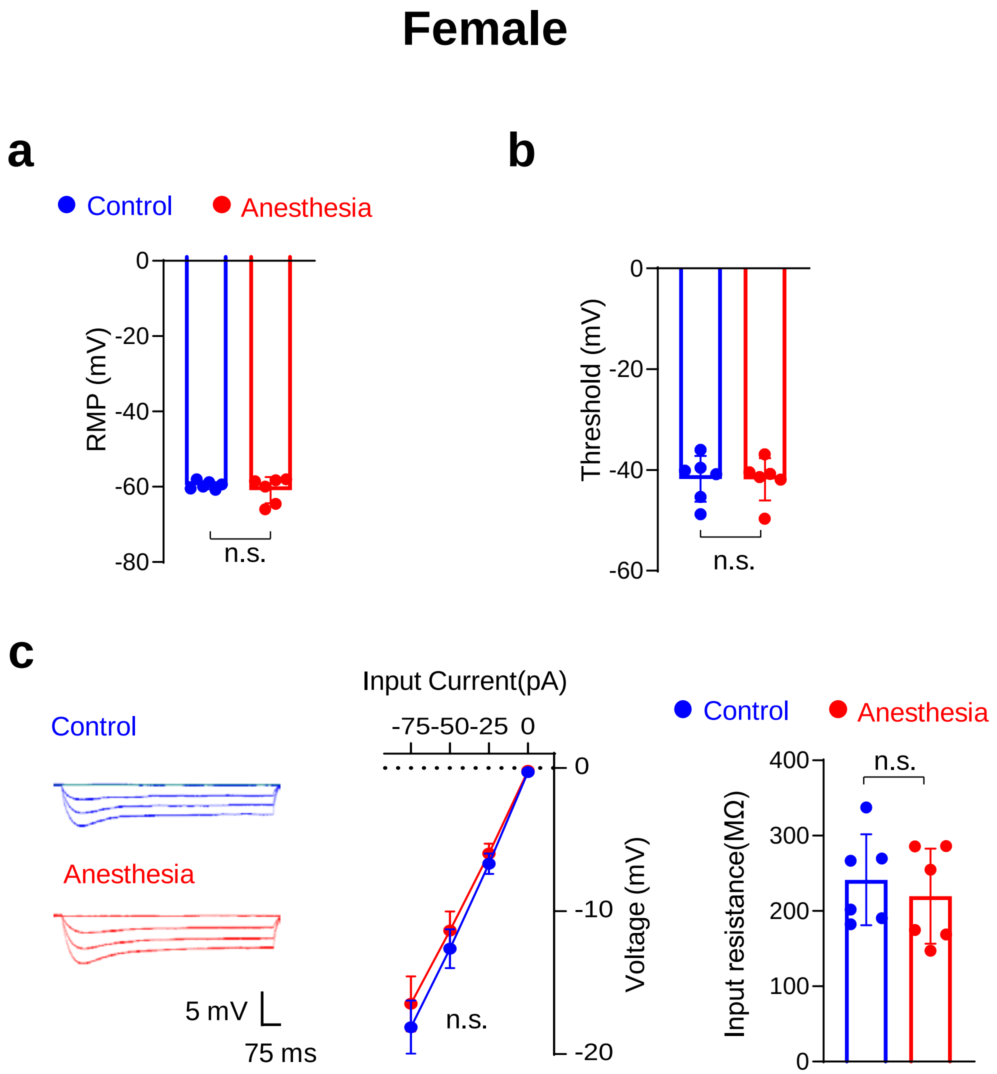

Supplementary Figure 9: Repeated ketamine anesthesia does not affect resting membrane potential (RMP), action potential firing thresholds, and input resistance in female mice.

Repeated ketamine anesthesia did not affect **a** Resting membrane potential (RMP) (Welch ANOVA, p = 0.417), **b** Action potential threshold (Student's t-test, p = 0.975), and **c** Input resistance (Student's t-test, p=0.555) (Control n = 6 mice [total 20 cells]; Anesthesia n = 6 mice [total 21 cells]). Values are presented as means ± SD. n.s. = not significant.

Supplementary Figure 10

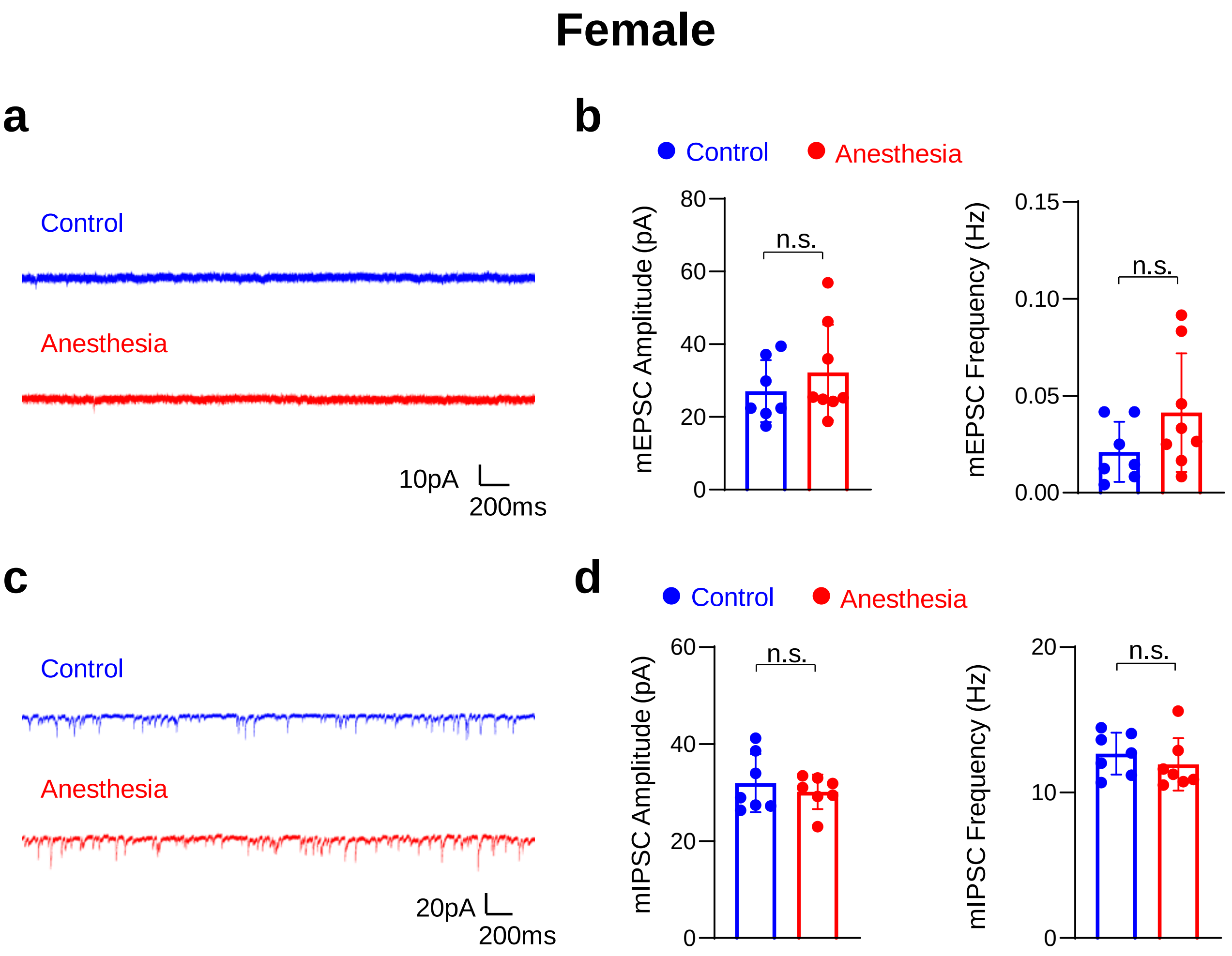

Supplementary Figure 10: Repeated ketamine anesthesia does not affect miniature excitatory/ inhibitory synaptic transmission (mEPSC/mIPSC) in hippocampal CA1 pyramidal neurons in late postnatal female mice.

mEPSCs/mIPSCs were measured 1 week after repeated saline injections (14 ml/kg, Control group) or ketamine anesthesia (35 mg/kg, Anesthesia group). **a** Example of mEPSCs. **b** Repeated ketamine anesthesia did not affect the amplitude (Student's t-test, p = 0.396) and frequency (Student’s t-test, p = 0.140) of mEPSCs (Control n = 7 mice [total 22 cells]; Anesthesia n = 8 mice [total 21 cells]). **c** Example of mIPSCs. **d** Repeated ketamine anesthesia did not affect the amplitude (Student’s t-test, p = 0.506) and frequency (Student’s t-test, p = 0.410) of mIPSCs (Control n = 7 mice [total 21 cells]; Anesthesia n = 7 mice[total 22 cells]). Values are presented as means ± SD. n.s. = not significant.

Supplementary tables

(Supplementary Table 1-11)

Supplementary Table 1

Supplementary Table 1: Summary of RNA-Seq mapping results. Raw reads were mapped to the Mus musculus genome using HISAT2.

	Sample ID	Total Reads	Mapped Reads	Unmapped Reads	Mapping Rate %
	K1-1	85,412,446	83,415,348	1,997,098	97.66
2	K2-1	77,017,482	75,396,088	1,621,394	97.89
3	K3-1	79,810,698	77,978,999	1,831,699	97.7
4	K4-1	76,976,782	75,209,308	1,767,474	97.7
5	S1-1	83,926,934	82,050,301	1,876,633	97.76
6	S2-1	89,503,340	87,662,414	1,840,926	97.94
7	S3-1	64,561,238	63,062,154	1,499,084	97.68
8	S4-1	77,762,164	75,875,467	1,886,697	97.57

Supplementary Table 2

Supplementary Table 2: Differential expression analysis between Control and Anesthesia group.

38 differentially expressed genes were identified using DESeq2 with pairwise comparison.

	HGNC symbol	Full name	baseMean	FC	p-value	adjusted p-value	direction
1	MAP1A	microtubule associated protein 1A	12,665.97	1.58	3.40E-09	2.07E-05	up
2	SZT2	SZT2, KICSTOR complex subunit	742.55	1.67	4.17E-09	2.07E-05	up
3	WDR90	WD repeat domain 90	395.83	1.62	3.09E-08	1.02E-04	up
4	DOT1L	DOT1 like histone lysine methyltransferase	360.11	1.48	3.91E-07	9.69E-04	up
5	SRGAP3	SLIT-ROBO Rho GTPase activating protein 3	4,312.37	1.31	1.44E-06	2.83E-03	up
6	SCN8A	sodium voltage-gated channel alpha subunit 8	8,531.85	1.2	1.72E-06	2.83E-03	up
7	MIAT	myocardial infarction associated transcript	8,420.01	1.39	2.33E-06	3.30E-03	up
8	ZFHX2	zinc finger homeobox 2	618.19	1.39	3.11E-06	3.86E-03	up
9	ZMYND8	zinc finger MYND-type containing 8	1,787.08	1.24	4.15E-06	4.57E-03	up
10	HDAC4	histone deacetylase 4	1,384.19	1.24	6.50E-06	6.44E-03	up
11	CBX6	chromobox 6	5,129.30	1.22	7.41E-06	6.68E-03	up
12	TUBGCP6	tubulin gamma complex associated protein 6	945.44	1.29	9.32E-06	7.70E-03	up
13	PPFIA3	PTPRF interacting protein alpha 3	1,754.01	1.2	1.39E-05	1.06E-02	up
14	MAPKBP1	mitogen-activated protein kinase binding protein 1	897.44	1.33	1.78E-05	1.16E-02	up
15	NAV2	neuron navigator 2	1,526.25	1.35	1.81E-05	1.16E-02	up
16	NEURL1B	neuralized E3 ubiquitin protein ligase 1B	861.73	1.43	1.87E-05	1.16E-02	up
17	PGPEP1	pyroglutamyl-peptidase I	283.78	-1.89	2.36E-05	1.38E-02	down
18	NAV1	neuron navigator 1	2,066.97	1.26	2.88E-05	1.59E-02	up
19	SRRM2	serine/arginine repetitive matrix 2	5,877.69	1.29	3.19E-05	1.66E-02	up
20	KCNQ2	potassium voltage-gated channel subfamily Q member 2	7,476.03	1.2	4.74E-05	2.35E-02	up
21	TOP2B	DNA topoisomerase II beta	4,060.62	-1.17	7.00E-05	3.15E-02	down
22	PRRC2B	proline rich coiled-coil 2B	5,377.15	1.38	7.51E-05	3.23E-02	up
23	LRP1	LDL receptor related protein 1	12,239.16	1.24	8.50E-05	3.51E-02	up
24	PCSK1N	proprotein convertase subtilisin/kexin type 1 inhibitor	778.08	1.32	1.03E-04	4.08E-02	up
25	NISCH	nischarin	5,128.98	1.15	1.14E-04	4.16E-02	up
26	PRRC2A	proline rich coiled-coil 2A	1,286.28	1.3	1.17E-04	4.16E-02	up
27	CHPF2	chondroitin polymerizing factor 2	1,640.00	1.28	1.17E-04	4.16E-02	up
28	PHLDB2	pleckstrin homology like domain family B member 2	256.98	-1.6	1.30E-04	4.44E-02	down
29	TMEM184B	transmembrane protein 184B	245.78	1.62	1.41E-04	4.67E-02	up
30	PLEC	plectin	3,828.15	1.37	1.51E-04	4.72E-02	up
31	CERS4	ceramide synthase 4	1,058.02	1.23	1.59E-04	4.72E-02	up
32	ABCA5	ATP binding cassette subfamily A member 5	2,071.70	-1.2	1.63E-04	4.72E-02	down
33	PTPN1	protein tyrosine phosphatase, non- receptor type 1	595.82	1.23	1.71E-04	4.72E-02	up
34	ZNF236	zinc finger protein 236	1,393.57	1.23	1.72E-04	4.72E-02	up
35	IQSEC3	IQ motif and Sec7 domain 3	1,758.34	1.23	1.93E-04	5.00E-02	up
36	PRRT3	proline rich transmembrane protein 3	673.65	1.31	1.93E-04	5.00E-02	up
37	SSH1	slingshot protein phosphatase 1	239.51	1.32	1.97E-04	5.00E-02	up
38	SRRT	serrate, RNA effector molecule	1,285.28	1.21	2.02E-04	5.00E-02	up

Supplementary Table 3

Supplementary Table. 3: Differential expression analysis was carried out using DESeq2 with pairwise comparison (Top 100). Majority of gene changes were less than 2-fold at the single-gene level.

Gene	HGNC	Ensembl ID	baseMean	log2FC	FC	p-value	adjusted p-
Map1a	MAP1A	ENSMUSG00000027254	12665.97231	0.660721894	1.580873462	3.40E-09	0.000020679
Szt2	SZT2	ENSMUSG00000033253	742.5537206	0.741792797	1.67225261	4.17E-09	0.000020679
Wdr90	WDR90	ENSMUSG00000073434	395.8348409	0.698256313	1.622542551	3.09E-08	0.000102215
Dot1l	DOT1L	ENSMUSG00000061589	360.1148716	0.565774626	1.48018205	3.91E-07	0.000969095
Srgap3	SRGAP3	ENSMUSG00000030257	4312.371075	0.390736215	1.311062276	1.44E-06	0.002833377
Scn8a	SCN8A	ENSMUSG00000023033	8531.853254	0.266591324	1.202962211	1.72E-06	0.002833377
Miat	MIAT	ENSMUSG00000097767	8420.012448	0.477192144	1.392031776	2.33E-06	0.003304382
Zfhx2	ZFHX2	ENSMUSG00000040721	618.1851095	0.473854476	1.38881504	3.11E-06	0.003858837
Zmynd8	ZMYND8	ENSMUSG00000039671	1787.077686	0.312682931	1.242015287	4.15E-06	0.00457074
Hdac4	HDAC4	ENSMUSG00000026313	1384.18703	0.305934093	1.236218785	6.50E-06	0.006440208
Cbx6	CBX6	ENSMUSG00000089715	5129.296461	0.288177828	1.221097017	7.41E-06	0.006678353
Tubgcp6	TUBGCP6	ENSMUSG00000051786	945.4398006	0.362060401	1.285260146	9.32E-06	0.007699383
Ppfia3	PPFIA3	ENSMUSG00000003863	1754.01428	0.26755134	1.203762968	1.39E-05	0.010600519
Mapkbp1	MAPKBP1	ENSMUSG00000033902	897.4414141	0.409608989	1.328325751	1.78E-05	0.011560003
Nav2	NAV2	ENSMUSG00000052512	1526.250446	0.436147404	1.352986462	1.81E-05	0.011560003
Neurl1b	NEURL1B	ENSMUSG00000034413	861.7304421	0.51122186	1.425256776	1.87E-05	0.011560003
Pgpep1	PGPEP1	ENSMUSG00000056204	283.7798296	-0.91756768	-1.88892795	2.36E-05	0.013762294
Nav1	NAV1	ENSMUSG00000009418	2066.966461	0.33606125	1.262305622	2.88E-05	0.015865098
Srrm2	SRRM2	ENSMUSG00000039218	5877.69434	0.36997812	1.292333231	3.19E-05	0.016633549
Kcnq2	KCNQ2	ENSMUSG00000016346	7476.03238	0.261034526	1.1983377	4.74E-05	0.02350255
Tspoap1	NA	ENSMUSG00000034156	3211.569189	0.315867309	1.244759744	5.33E-05	0.025139716
Top2b	TOP2B	ENSMUSG00000017485	4060.616757	-0.22444531	-1.16832796	7.00E-05	0.031546514
Prrc2b	PRRC2B	ENSMUSG00000039262	5377.154237	0.464134765	1.379489776	7.51E-05	0.032346407
Lrp1	LRP1	ENSMUSG00000040249	12239.1621	0.314073203	1.243212749	8.50E-05	0.035118586
Pcsk1n	PCSK1N	ENSMUSG00000039278	778.0843823	0.395987273	1.315842917	0.000102954	0.040815133
Nisch	NISCH	ENSMUSG00000021910	5128.983767	0.202742014	1.150883669	0.000114087	0.041575114
Prrc2a	PRRC2A	ENSMUSG00000024393	1286.279599	0.378173426	1.299695289	0.000117186	0.041575114
Chpf2	CHPF2	ENSMUSG00000038181	1639.99892	0.354866215	1.278866983	0.000117456	0.041575114
Phldb2	PHLDB2	ENSMUSG00000033149	256.9830424	-0.67898278	-1.60101051	0.000130039	0.044441825
Tmem184b	TMEM184	ENSMUSG00000009035	245.7809012	0.69565999	1.619625194	0.000141236	0.046659582
Plec	PLEC	ENSMUSG00000022565	3828.154398	0.455397929	1.371160951	0.000151026	0.04723263
Cers4	CERS4	ENSMUSG00000008206	1058.021529	0.301746826	1.232635995	0.000158645	0.04723263
Abca5	ABCA5	ENSMUSG00000018800	2071.701074	-0.26417806	-1.20095164	0.000163027	0.04723263
Gm42771	NA	ENSMUSG00000106579	444.0472856	0.462498981	1.377926543	0.000168777	0.04723263
Ptpn1	PTPN1	ENSMUSG00000027540	595.8182854	0.294599358	1.226544314	0.000171107	0.04723263
Zfp236	ZNF236	ENSMUSG00000041258	1393.56767	0.293241139	1.225390132	0.000171564	0.04723263
Iqsec3	IQSEC3	ENSMUSG00000040797	1758.341552	0.298417268	1.229794507	0.000192556	0.049951829
Prrt3	PRRT3	ENSMUSG00000045009	673.6530226	0.393394452	1.313480199	0.000192781	0.049951829
Ssh1	SSH1	ENSMUSG00000042121	239.5139321	0.403654436	1.322854543	0.000196953	0.049951829
Srrt	SRRT	ENSMUSG00000037364	1285.276769	0.279172275	1.213498457	0.000201602	0.049951829
Marf1	MARF1	ENSMUSG00000060657	3485.459124	0.215659106	1.161234312	0.00022832	0.054162199
Slc36a1	SLC36A1	ENSMUSG00000020261	619.1708971	0.491973161	1.406367036	0.000235439	0.054162199
Dpysl5	DPYSL5	ENSMUSG00000029168	303.3220633	0.473209533	1.388194322	0.000247039	0.054162199
Ppp1r12b	PPP1R12B	ENSMUSG00000073557	2636.095738	0.295643177	1.227432065	0.000248451	0.054162199
Adgrb2	ADGRB2	ENSMUSG00000028782	5081.931847	0.313374665	1.242610943	0.00025038	0.054162199
Csmd2	CSMD2	ENSMUSG00000028804	1277.638798	0.390875537	1.311188892	0.000251383	0.054162199
Gan	GAN	ENSMUSG00000052557	624.6311088	0.449383546	1.365456681	0.000263797	0.05562746
Zfx	ZFX	ENSMUSG00000079509	1124.707212	-0.29026776	-1.22286722	0.000274843	0.056749279
Plekhm2	PLEKHM2	ENSMUSG00000028917	800.9076446	0.262359274	1.199438573	0.000286404	0.057929598
Ppfia4	PPFIA4	ENSMUSG00000026458	1181.215663	0.258711762	1.196409907	0.000296775	0.058826658
Sbf1	SBF1	ENSMUSG00000036529	4875.500541	0.395120541	1.315052632	0.000331256	0.06437408
Ttyh3	TTYH3	ENSMUSG00000036565	760.512075	0.561352038	1.475651493	0.000357446	0.068127896
D630045J12Rik	KIAA1549	ENSMUSG00000063455	1055.003359	0.287010358	1.220109271	0.000372891	0.069730589
Rapgef1	RAPGEF1	ENSMUSG00000039844	1056.352629	0.397745361	1.317447399	0.000404055	0.073770608
Hspa5	HSPA5	ENSMUSG00000026864	4320.535778	-0.30800416	-1.23799386	0.000418257	0.073770608
Zfp652	ZNF652	ENSMUSG00000075595	1489.217417	0.31264217	1.241980197	0.000423792	0.073770608
Malsu1	MALSU1	ENSMUSG00000029815	349.5893421	0.414249108	1.332604904	0.000424268	0.073770608
Ddx1	DDX1	ENSMUSG00000037149	2488.990903	-0.22118095	-1.16568739	0.000440125	0.074241704
Atp8a2	ATP8A2	ENSMUSG00000021983	1314.674346	0.527933417	1.441862325	0.000441959	0.074241704
Crtc1	CRTC1	ENSMUSG00000003575	758.679754	0.373898701	1.295849974	0.000459689	0.075933035
Atp13a2	ATP13A2	ENSMUSG00000036622	2270.912643	0.199750389	1.148499628	0.000487563	0.079216969
Rab11fip3	RAB11FIP	ENSMUSG00000037098	1250.389118	0.280998643	1.215035649	0.000526467	0.084158261
Actr6	ACTR6	ENSMUSG00000019948	366.9520732	-0.39084694	-1.3111629	0.000543681	0.085066287
Bcl11a	BCL11A	ENSMUSG00000000861	1805.389264	0.268015822	1.204150587	0.000549313	0.085066287
Dnmt3a	DNMT3A	ENSMUSG00000020661	2582.684713	0.40090916	1.320339703	0.000562432	0.085757958
Gon4l	GON4L	ENSMUSG00000054199	1314.619639	0.250483792	1.18960597	0.000585317	0.086619966
Hectd4	NA	ENSMUSG00000042744	8910.13598	0.288470173	1.221344483	0.000591447	0.086619966
Dusp7	DUSP7	ENSMUSG00000053716	591.2369282	0.316433145	1.245248044	0.000594305	0.086619966
Smg9	SMG9	ENSMUSG00000002210	301.9575774	0.350793534	1.275261874	0.000613848	0.088171707
Soga1	SOGA1	ENSMUSG00000055485	1033.125844	0.393644055	1.313707466	0.000645288	0.09037316
Myo18a	MYO18A	ENSMUSG00000000631	2428.517479	0.276634436	1.211365674	0.000655486	0.09037316
Ccnyl1	CCNYL1	ENSMUSG00000070871	265.1978515	0.555982911	1.470169922	0.00065653	0.09037316
Srcap	SRCAP	ENSMUSG00000053877	999.0405197	0.268312951	1.204398613	0.000668655	0.090781384
Map1s	MAP1S	ENSMUSG00000019261	650.6413257	0.470927102	1.38599985	0.00068661	0.09120386
Med13l	MED13L	ENSMUSG00000018076	2322.254861	0.280608484	1.214707102	0.000702343	0.09120386
Kcnb1	KCNB1	ENSMUSG00000050556	3537.336573	0.471338265	1.386394912	0.00071262	0.09120386
Phkb	PHKB	ENSMUSG00000036879	890.5537466	-0.31643997	-1.24525394	0.000713143	0.09120386
Rapgef3	RAPGEF3	ENSMUSG00000022469	549.538955	0.274910264	1.20991883	0.000717778	0.09120386
Adgrb1	ADGRB1	ENSMUSG00000034730	2239.478481	0.354086597	1.278176083	0.000771836	0.096831281
Ubr4	UBR4	ENSMUSG00000066036	6994.026329	0.198294078	1.147340874	0.000802007	0.099358607
Cacna1a	CACNA1	ENSMUSG00000034656	1828.935772	0.45390828	1.369745895	0.000840746	0.102871961
Arhgap35	ARHGAP3	ENSMUSG00000058230	4548.468917	0.16495838	1.121133734	0.000855742	0.103429998
Fbxo31	FBXO31	ENSMUSG00000052934	1587.314624	0.279024874	1.21337448	0.000868527	0.103710519
Crebbp	CREBBP	ENSMUSG00000022521	2354.837888	0.233553502	1.17572732	0.000879193	0.103734302
Sipa1l1	SIPA1L1	ENSMUSG00000042700	5106.07045	0.262608475	1.199645773	0.000891706	0.10397297

Gcn1	GCN1	ENSMUSG00000041638	2124.814031	0.230145127	1.172952936	0.000920132	0.106039902
Mapk11	MAPK11	ENSMUSG00000053137	341.3816259	0.441721541	1.358224104	0.00094009	0.106365439
Fam193b	FAM193B	ENSMUSG00000021495	1031.871415	0.291691916	1.224074967	0.000949943	0.106365439
Ccdc85c	CCDC85C	ENSMUSG00000084883	694.0426375	0.269108426	1.205062878	0.000955153	0.106365439
Denn2b	NA	ENSMUSG00000031024	273.4589087	-0.48654202	-1.40108261	0.000985488	0.108524153
Adgrl1	ADGRL1	ENSMUSG00000013033	6879.615383	0.384284201	1.30521204	0.000996832	0.108567055
Kcnq5	KCNQ5	ENSMUSG00000028033	1974.765006	0.281897032	1.215792506	0.001013591	0.109192343
Ralgapb	RALGAPB	ENSMUSG00000027652	3605.877901	0.218976104	1.163907256	0.001030101	0.109777731
Neurl1a	NEURL1	ENSMUSG00000006435	1484.843126	0.251189268	1.190187827	0.001085586	0.113785855
Rbfox3	RBFOX3	ENSMUSG00000025576	930.4715509	0.299635069	1.230833034	0.001090673	0.113785855
Sptbn4	SPTBN4	ENSMUSG00000011751	2543.399852	0.249168494	1.188521906	0.001140336	0.117727766
Palm	PALM	ENSMUSG00000035863	1107.244605	0.24688313	1.186640666	0.001165799	0.11911581
Wdfy3	WDFY3	ENSMUSG00000043940	7200.636565	0.205664219	1.153217168	0.001206517	0.12155176

Supplementary Table 4

Supplementary Table. 4: Functional enrichment analysis of differentially expression genes (DEGs) using Gene ontological term (GO) Molecular Function analysis.

Term	Overlap	P-value	Adjusted P-value	Odds Ratio	Combined Score	Genes
ankyrin binding (GO:0030506)	2/18	5.28E-04	0.607246	58.47953	441.3572	KCNQ2;PLEC
RNA binding (GO:0003723)	8/1387	0.003942	1	3.035708	16.80575	SRRM2;MAPKBP1;PTPN1;LRP1;SRRT;PRRC2B;PRRC2A;PLEC
repressing transcription factor binding (GO:0070491)	2/53	0.004557	1	19.86097	107.0728	HDAC4;ZMYND8
clathrin heavy chain binding (GO:0032050)	1/6	0.011347	1	87.7193	392.8751	LRP1
sphingosine N-acyltransferase activity (GO:0050291)	1/6	0.011347	1	87.7193	392.8751	CERS4
histone deacetylase binding (GO:0042826)	2/85	0.011364	1	12.3839	55.4466	HDAC4;TOP2B
microtubule minus-end binding (GO:0051011)	1/10	0.018842	1	52.63158	209.0339	TUBGCP6
cadherin binding (GO:0045296)	3/313	0.021386	1	5.04456	19.39634	PTPN1;PHLDB2;PLEC
C2H2 zinc finger domain binding (GO:0070742)	1/12	0.022569	1	43.85965	166.2793	SRRM2
potassium ion binding (GO:0030955)	1/12	0.022569	1	43.85965	166.2793	HDAC4
low-density lipoprotein receptor activity (GO:0005041)	1/13	0.024427	1	40.48583	150.2853	LRP1
lipoprotein transporter activity (GO:0042954)	1/14	0.026282	1	37.59398	136.7993	LRP1
phosphoprotein phosphatase activity (GO:0004721)	2/136	0.027507	1	7.739938	27.8121	PTPN1;SSH1
lipoprotein particle receptor activity (GO:0030228)	1/15	0.028134	1	35.08772	125.2909	LRP1
alkali metal ion binding (GO:0031420)	1/16	0.029982	1	32.89474	115.3676	HDAC4
lysine-acetylated histone binding (GO:0070577)	1/18	0.033667	1	29.23977	99.15889	ZMYND8
acetylation-dependent protein binding (GO:0140033)	1/18	0.033667	1	29.23977	99.15889	ZMYND8
ephrin receptor binding (GO:0046875)	1/19	0.035505	1	27.70083	92.4678	PTPN1
gamma-tubulin binding (GO:0043015)	1/19	0.035505	1	27.70083	92.4678	TUBGCP6
scavenger receptor activity (GO:0005044)	1/21	0.03917	1	25.06266	81.19913	LRP1
voltage-gated ion channel activity involved in regulation of postsynaptic membrane potential (GO:1905030)	1/23	0.042822	1	22.8833	72.09872	SCN8A
voltage-gated sodium channel activity (GO:0005248)	1/23	0.042822	1	22.8833	72.09872	SCN8A
N-acyltransferase activity (GO:0016410)	1/25	0.04646	1	21.05263	64.61414	CERS4
lipoprotein particle receptor binding (GO:0070325)	1/25	0.04646	1	21.05263	64.61414	LRP1
RNA polymerase II transcription corepressor activity (GO:0001106)	1/26	0.048274	1	20.24291	61.35364	ZMYND8
microtubule binding (GO:0008017)	2/195	0.052872	1	5.398111	15.86982	MAP1A;TUBGCP6
delayed rectifier potassium channel activity (GO:0005251)	1/29	0.053695	1	18.14882	53.0749	KCNQ2
glucuronosyltransferase activity (GO:0015020)	1/33	0.060878	1	15.94896	44.63934	CHPF2
protein tyrosine/serine/threonine phosphatase activity (GO:0008138)	1/34	0.062665	1	15.47988	42.8785	SSH1
endopeptidase regulator activity (GO:0061135)	1/36	0.06623	1	14.61988	39.68752	PCSK1N
peptidase inhibitor activity (GO:0030414)	1/36	0.06623	1	14.61988	39.68752	PCSK1N
receptor tyrosine kinase binding (GO:0030971)	1/39	0.071552	1	13.49528	35.59153	PTPN1
sodium channel activity (GO:0005272)	1/40	0.073319	1	13.15789	34.38066	SCN8A
histone deacetylase activity (GO:0004407)	1/40	0.073319	1	13.15789	34.38066	HDAC4
protein kinase C binding (GO:0005080)	1/40	0.073319	1	13.15789	34.38066	TOP2B
protein methyltransferase activity (GO:0008276)	1/41	0.075084	1	12.83697	33.23688	DOT1L
histone-lysine N-methyltransferase activity (GO:0018024)	1/41	0.075084	1	12.83697	33.23688	DOT1L
protein deacetylase activity (GO:0033558)	1/42	0.076845	1	12.53133	32.15502	HDAC4
hydrolase activity, acting on acid anhydrides, catalyzing transmembrane movement of substances (GO:00168	1/42	0.076845	1	12.53133	32.15502	ABCA5
Rac GTPase binding (GO:0048365)	1/43	0.078602	1	12.2399	31.13042	SRGAP3
ATPase activity, coupled to transmembrane movement of substances (GO:0042626)	1/45	0.082108	1	11.69591	29.23651	ABCA5
P-P-bond-hydrolysis-driven transmembrane transporter activity (GO:0015405)	1/45	0.082108	1	11.69591	29.23651	ABCA5
actin binding (GO:0003779)	2/254	0.083828	1	4.144219	10.27347	MAP1A;SSH1
tubulin binding (GO:0015631)	2/255	0.084392	1	4.127967	10.20549	MAP1A;TUBGCP6
transcriptional repressor activity, RNA polymerase II transcription factor binding (GO:0001191)	1/47	0.0856	1	11.19821	27.52592	ZMYND8
protein-lysine N-methyltransferase activity (GO:0016279)	1/47	0.0856	1	11.19821	27.52592	DOT1L
histone methyltransferase activity (GO:0042054)	1/48	0.087342	1	10.96491	26.73163	DOT1L
protein tyrosine kinase binding (GO:1990782)	1/48	0.087342	1	10.96491	26.73163	PTPN1
methylation-dependent protein binding (GO:0140034)	1/52	0.094275	1	10.12146	23.90217	ZMYND8
methylated histone binding (GO:0035064)	1/55	0.099442	1	9.569378	22.08786	ZMYND8
ATPase activity, coupled to movement of substances (GO:0043492)	1/56	0.101158	1	9.398496	21.53265	ABCA5
transferase activity, transferring acyl groups other than amino-acyl groups (GO:0016747)	1/56	0.101158	1	9.398496	21.53265	CERS4
protein tyrosine phosphatase activity (GO:0004725)	1/62	0.111386	1	8.488964	18.63121	PTPN1
zinc ion binding (GO:0008270)	2/302	0.112153	1	3.485535	7.62598	HDAC4;PTPN1
activating transcription factor binding (GO:0033613)	1/68	0.1215	1	7.739938	16.31453	HDAC4
Rho GTPase binding (GO:0017048)	1/72	0.128181	1	7.309942	15.01689	SRGAP3
DNA-dependent ATPase activity (GO:0008094)	1/72	0.128181	1	7.309942	15.01689	TOP2B
hydrolase activity, acting on carbon-nitrogen (but not peptide) bonds, in linear amides (GO:0016811)	1/75	0.133159	1	7.017544	14.14884	HDAC4
protein transporter activity (GO:0008565)	1/80	0.141395	1	6.578947	12.86975	LRP1
RNA polymerase II transcription cofactor activity (GO:0001104)	1/84	0.147928	1	6.265664	11.97388	ZMYND8
voltage-gated potassium channel activity (GO:0005249)	1/85	0.149554	1	6.19195	11.76533	KCNQ2
ion channel activity (GO:0005216)	1/93	0.162451	1	5.65931	10.28509	SCN8A
core promoter binding (GO:0001047)	1/97	0.168829	1	5.425936	9.652037	HDAC4
cation transmembrane transporter activity (GO:0008324)	1/97	0.168829	1	5.425936	9.652037	SCN8A
voltage-gated cation channel activity (GO:0022843)	1/100	0.173581	1	5.263158	9.216384	KCNQ2
potassium channel activity (GO:0005267)	1/100	0.173581	1	5.263158	9.216384	KCNQ2
transition metal ion binding (GO:0046914)	2/399	0.175256	1	2.638174	4.594399	HDAC4;PTPN1
phosphatase activity (GO:0016791)	1/104	0.179875	1	5.060729	8.681634	SSH1
endopeptidase inhibitor activity (GO:0004866)	1/111	0.190779	1	4.741584	7.855093	PCSK1N
RNA polymerase II transcription factor binding (GO:0001085)	1/121	0.206111	1	4.349717	6.869679	HDAC4
protein kinase binding (GO:0019901)	2/495	0.242039	1	2.126528	3.016812	TOP2B;PTPN1
cation channel activity (GO:0005261)	1/146	0.243215	1	3.604903	5.09665	SCN8A
RNA polymerase II regulatory region DNA binding (GO:0001012)	1/201	0.318997	1	2.618487	2.991814	ZMYND8
transcription corepressor activity (GO:0003714)	1/203	0.321609	1	2.592689	2.941198	ZMYND8
regulatory region DNA binding (GO:0000975)	1/224	0.348449	1	2.349624	2.477125	HDAC4
GTPase activator activity (GO:0005096)	1/249	0.379055	1	2.113718	2.050464	SRGAP3
protein heterodimerization activity (GO:0046982)	1/265	0.397903	1	1.986097	1.830281	TOP2B

GTPase regulator activity (GO:0030695)	1/275	0.409399	1	1.913876	1.709214	SRGAP3
calcium ion binding (GO:0005509)	1/284	0.419563	1	1.853225	1.609603	LRP1
transcription regulatory region sequence-specific DNA binding (GO:0000976)	1/292	0.428454	1	1.802451	1.527707	ZMYND8
DNA binding (GO:0003677)	2/893	0.510875	1	1.178759	0.79169	HDAC4;SRRT
transcription regulatory region DNA binding (GO:0044212)	1/374	0.512272	1	1.407261	0.941317	HDAC4
sequence-specific DNA binding (GO:0043565)	1/394	0.530824	1	1.335827	0.846012	HDAC4
kinase binding (GO:0019900)	1/418	0.552181	1	1.259129	0.74777	PTPN1
metal ion binding (GO:0046872)	1/442	0.57259	1	1.19076	0.663949	LRP1
RNA polymerase II regulatory region sequence-specific DNA binding (GO:0000977)	1/460	0.5873	1	1.144165	0.608947	ZMYND8

Supplementary Table 5

Supplementary Table. 5: Functional enrichment analysis of differentially expression genes (DEGs): Gene ontological term (GO) Cellular Component.

Differentially expressed genes were associated with several cellular component categories (marked in red)

Term	Overlap	P-value	Adjusted P-value	Odds Ratio	Combined Score	Genes
**axon initial segment (GO:0043194)**	**3/9**	**5.27E-07**	**2.35E-04**	**175.439**	**2536.044**	**SCN8A;KCNQ2;NAV1**
**main axon (GO:0044304)**	**3/33**	**3.32E-05**	**0.007401**	**47.8469**	**493.4569**	**SCN8A;KCNQ2;NAV1**
**node of Ranvier (GO:0033268)**	**2/12**	**2.29E-04**	**0.034078**	**87.7193**	**735.1581**	**SCN8A;KCNQ2**
intermediate filament cytoskeleton (GO:0045111)	2/71	0.0080431	0.8968047	14.8258	71.503955	PHLDB2;PLEC
early endosome (GO:0005769)	3/222	0.0085513	0.7627719	7.112376	33.866837	NEURL1B;PTPN1;LRP1
lytic vacuole membrane (GO:0098852)	3/233	0.0097525	0.7249363	6.776598	31.377214	SZT2;LRP1;ABCA5
spindle pole body (GO:0005816)	1/6	0.0113473	0.7229844	87.7193	392.87513	TUBGCP6
gamma-tubulin ring complex (GO:0008274)	1/8	0.0151018	0.8419253	65.78947	275.85141	TUBGCP6
gamma-tubulin large complex (GO:0000931)	1/8	0.0151018	0.748378	65.78947	275.85141	TUBGCP6
lysosomal membrane (GO:0005765)	3/291	0.0176692	0.7880442	5.425936	21.898725	SZT2;LRP1;ABCA5
focal adhesion (GO:0005925)	3/356	0.0298038	1	4.435245	15.581541	LRP1;PHLDB2;PLEC
U5 snRNP (GO:0005682)	1/23	0.0428215	1	22.8833	72.098715	MAPKBP1
lysosome (GO:0005764)	3/422	0.0456658	1	3.741581	11.548037	SZT2;LRP1;ABCA5
mitotic spindle pole (GO:0097431)	1/25	0.0464596	1	21.05263	64.614136	MAPKBP1
U2-type catalytic step 2 spliceosome (GO:0071007)	1/29	0.0536955	1	18.14882	53.074899	SRRM2
sarcoplasm (GO:0016528)	1/31	0.0572933	1	16.97793	48.549606	PLEC
precatalytic spliceosome (GO:0071011)	1/33	0.0608778	1	15.94896	44.639341	MAPKBP1
Cajal body (GO:0015030)	1/34	0.0626651	1	15.47988	42.878497	SRRM2
heterochromatin (GO:0000792)	1/41	0.0750836	1	12.83697	33.236879	TOP2B
cytoskeleton (GO:0005856)	3/520	0.0754818	1	3.036437	7.8457408	NAV1;PHLDB2;PLEC
spliceosomal snRNP complex (GO:0097525)	1/59	0.106286	1	8.920607	19.996628	MAPKBP1
U2-type spliceosomal complex (GO:0005684)	1/60	0.107989	1	8.77193	19.523907	SRRM2
mitotic spindle (GO:0072686)	1/84	0.1479279	1	6.265664	11.973875	MAPKBP1
peroxisome (GO:0005777)	1/92	0.1608497	1	5.720824	10.453575	SZT2
microbody (GO:0042579)	1/92	0.1608497	1	5.720824	10.453575	SZT2
microtubule cytoskeleton (GO:0015630)	2/388	0.16781	1	2.712968	4.8424394	MAP1A;NAV1
spindle pole (GO:0000922)	1/107	0.1845658	1	4.918839	8.3116042	MAPKBP1
spliceosomal complex (GO:0005681)	1/114	0.1954088	1	4.616805	7.5376812	MAPKBP1
late endosome (GO:0005770)	1/140	0.2344669	1	3.759398	5.4527847	ABCA5
lytic vacuole (GO:0000323)	1/183	0.2950474	1	2.876043	3.5105525	ABCA5
microtubule (GO:0005874)	1/210	0.3306728	1	2.506266	2.7734988	MAP1A
dendrite (GO:0030425)	1/215	0.3370748	1	2.44798	2.662057	MAP1A
polymeric cytoskeletal fiber (GO:0099513)	1/221	0.3446787	1	2.381519	2.536658	MAP1A
nuclear speck (GO:0016607)	1/296	0.4328497	1	1.778094	1.4889132	SRRM2
chromatin (GO:0000785)	1/296	0.4328497	1	1.778094	1.4889132	TOP2B
Golgi membrane (GO:0000139)	1/442	0.5725904	1	1.19076	0.6639494	CHPF2
centrosome (GO:0005813)	1/461	0.5881026	1	1.141683	0.6060667	TUBGCP6
Golgi subcompartment (GO:0098791)	1/479	0.602292	1	1.09878	0.5570958	CHPF2
microtubule organizing center (GO:0005815)	1/507	0.6234233	1	1.038098	0.4905321	TUBGCP6
nuclear body (GO:0016604)	1/618	0.6969457	1	0.851644	0.3074841	SRRM2
integral component of plasma membrane (GO:0005887)	2/1463	0.7772761	1	0.719502	0.1812855	LRP1;KCNQ2

Supplementary Table 6

Supplementary Table. 6: Functional enrichment analysis of differentially expression genes (DEGs) using KEGG pathway analysis.

Term	Overlap	P-value	Adjusted P-value	Odds Ratio	Combined Score	Genes
Axon guidance	2/181	0.046289	1	5.815644	17.870576	SRGAP3;SSH1
ABC transporters	1/45	0.082108	1	11.69591	29.236511	ABCA5
Sphingolipid metabolism	1/47	0.0856	1	11.19821	27.52592	CERS4
Malaria	1/49	0.08908	1	10.74114	25.974426	LRP1
Cholesterol metabolism	1/50	0.090815	1	10.52632	25.25189	LRP1
Glycosaminoglycan biosynthesis	1/53	0.096001	1	9.930487	23.271088	CHPF2
Lysine degradation	1/59	0.106286	1	8.920607	19.996628	DOT1L
Adherens junction	1/72	0.128181	1	7.309942	15.016894	PTPN1
Insulin resistance	1/108	0.186123	1	4.873294	8.193689	PTPN1
Cholinergic synapse	1/112	0.192325	1	4.699248	7.7470287	KCNQ2
Sphingolipid signaling pathway	1/119	0.203068	1	4.422822	7.0509343	CERS4
Insulin signaling pathway	1/137	0.230056	1	3.841721	5.6451463	PTPN1
Apelin signaling pathway	1/137	0.230056	1	3.841721	5.6451463	HDAC4
Alzheimer disease	1/171	0.278628	1	3.07787	3.9331463	LRP1
Alcoholism	1/180	0.290977	1	2.923977	3.6096822	HDAC4
Transcriptional misregulation in cancer	1/186	0.299095	1	2.829655	3.4153745	DOT1L
Viral carcinogenesis	1/201	0.318997	1	2.618487	2.9918138	HDAC4
Regulation of actin cytoskeleton	1/214	0.335799	1	2.45942	2.6838216	SSH1
Endocytosis	1/244	0.373048	1	2.157032	2.1269387	IQSEC3
MicroRNAs in cancer	1/299	0.436125	1	1.760253	1.4607055	HDAC4

Supplementary Table 7

Supplementary Table. 7: Gene set enrichment analysis (GSEA) identification of the up- and down-regulated GO-biological processes after early repeated ketamine anesthesia (top 10). Significant changes are marked in red.

Up regulated

NAME	SIZE	ES	NES	NOM p-val	FDR q-val
GO_CELL_MORPHOGENESIS_INVOLVED_IN_NEURON_DIFFERENTIATION	441	0.18118502	4.3243165	0	0
GO_SYNAPTIC_SIGNALING	483	0.164537	4.029498	0	0
GO_REGULATION_OF_TRANS_SYNAPTIC_SIGNALING	328	0.19423412	4.0293503	0	0
GO_SYNAPSE_ORGANIZATION	317	0.19819386	4.0129027	0	0
GO_REGULATION_OF_NEURON_DIFFERENTIATION	469	0.16322348	3.9294293	0	0
GO_REGULATION_OF_NEURON_PROJECTION_DEVELOPMENT	383	0.17499566	3.8295906	0	0
GO_NERVOUS_SYSTEM_PROCESS	488	0.15375303	3.8240967	0	0
GO_POSITIVE_REGULATION_OF_NERVOUS_SYSTEM_DEVELOPMENT	369	0.16713043	3.642433	0	0
GO_REGULATION_OF_CELL_MORPHOGENESIS_INVOLVED_IN_DIFFERENTIATION	237	0.19911109	3.60875	0	0
GO_AXON_DEVELOPMENT	377	0.1621545	3.5804563	0	0

Down regulated

NAME	SIZE	ES	NES	NOM p-val	FDR q-val
GO_ORGANIC_ACID_METABOLIC_PROCESS	485	-0.2270723	-5.596495	0	0
GO_GENERATION_OF_PRECURSOR_METABOLITES_AND_ENERGY	326	-0.2176701	-4.5525103	0	0
GO_TRANSLATIONAL_INITIATION	164	-0.3015388	-4.4902916	0	0
GO_ESTABLISHMENT_OF_PROTEIN_LOCALIZATION_TO_ORGANELLE	418	-0.1864161	-4.2972703	0	0
GO_COTRANSLATIONAL_PROTEIN_TARGETING_TO_MEMBRANE	93	-0.3831721	-4.2645864	0	0
GO_ENERGY_DERIVATION_BY_OXIDATION_OF_ORGANIC_COMPOUNDS	198	-0.2507331	-4.122318	0	0
GO_ESTABLISHMENT_OF_PROTEIN_LOCALIZATION_TO_ENDOPLASMIC_RETICULUM	104	-0.351114	-4.0865355	0	0
GO_COFACTOR_METABOLIC_PROCESS	236	-0.2281749	-3.9893894	0	0
GO_CELLULAR_AMINO_ACID_METABOLIC_PROCESS	185	-0.2533908	-3.986226	0	0
GO_CELLULAR_RESPIRATION	149	-0.2795848	-3.984667	0	0

Supplementary Table 8

Supplementary Table. 8: Gene set enrichment analysis (GSEA) identification of the up- and down-regulated GO-molecular function after early repeated ketamine anesthesia (top 10). Significant changes are marked in red.

Up regulated

NAME	SIZE	ES	NES	NOM p-val	FDR q-val
GO_CATION_CHANNEL_ACTIVITY	161	0.24580063	3.6102238	0	0
GO_VOLTAGE_GATED_CATION_CHANNEL_ACTIVITY	81	0.33119586	3.5280998	0	0
GO_GATED_CHANNEL_ACTIVITY	170	0.2332068	3.4975314	0	0
GO_PASSIVE_TRANSMEMBRANE_TRANSPORTER_ACTIVITY	213	0.20930183	3.4619002	0	0
GO_METAL_ION_TRANSMEMBRANE_TRANSPORTER_ACTIVITY	241	0.18717594	3.4302657	0	0
GO_VOLTAGE_GATED_ION_CHANNEL_ACTIVITY	108	0.28293413	3.4248512	0	0
GO_REGULATORY_REGION_NUCLEIC_ACID_BINDING	448	0.13735116	3.273669	0	0
GO_TRANSMEMBRANE_SIGNALING_RECEPTOR_ACTIVITY	264	0.1643355	3.119773	0	0
GO_SEQUENCE_SPECIFIC_DOUBLE_STRANDED_DNA_BINDING	406	0.13138528	2.9234815	0	6.03E-04
GO_POTASSIUM_CHANNEL_ACTIVITY	65	0.3158592	2.9101298	0	5.42E-04

Down regulated

NAME	SIZE	ES	NES	NOM p-val	FDR q-val
GO_STRUCTURAL_CONSTITUENT_OF_RIBOSOME	140	-0.3625131	-4.9154854	0	0
GO_OXIDOREDUCTASE_ACTIVITY	380	-0.2054846	-4.6645784	0	0
GO_ELECTRON_TRANSFER_ACTIVITY	71	-0.3483952	-3.5111516	0	0
GO_UNFOLDED_PROTEIN_BINDING	92	-0.3063211	-3.4522877	0	0
GO_COFACTOR_BINDING	257	-0.1882064	-3.4358764	0	0
GO_STRUCTURAL_MOLECULE_ACTIVITY	422	-0.1360299	-3.1657846	0	0
GO_COENZYME_BINDING	167	-0.2015422	-3.064216	0	0
GO_OXIDOREDUCTASE_ACTIVITY_ACTING_ON_A_SULFUR_GROUP_OF_DONORS	39	-0.3863761	-2.844314	0	1.24E-04
GO_CARBON_OXYGEN_LYASE_ACTIVITY	37	-0.3907485	-2.8337123	0	1.11E-04
GO_VITAMIN_B6_BINDING	23	-0.4779838	-2.768192	0	1.92E-04

Supplementary Table 9

Supplementary Table. 9: Gene set enrichment analysis (GSEA) identification of the up- and down-regulated GO-cellular components after early repeated ketamine anesthesia (top 10). Significant changes are marked in red.

Up regulated

NAME	SIZE	ES	NES	NOM p-val	FDR q-val
GO_AXON	465	0.1885717	4.5603476	0	0
GO_SYNAPTIC_MEMBRANE	333	0.22234212	4.522456	0	0
GO_NEURON_TO_NEURON_SYNAPSE	308	0.21065162	4.2779417	0	0
GO_POSTSYNAPTIC_MEMBRANE	249	0.22514796	4.138114	0	0
GO_AXON_PART	301	0.18656564	3.7905285	0	0
GO_PRESYNAPTIC_MEMBRANE	127	0.28577015	3.692235	0	0
GO_PRESYNAPSE	373	0.15829703	3.5496469	0	0
GO_PLASMA_MEMBRANE_PROTEIN_COMPLEX	303	0.1749063	3.5438	0	0
GO_PRESYNAPTIC_ACTIVE_ZONE	58	0.38782075	3.5294006	0	0.00E+00
GO_MAIN_AXON	58	0.38558477	3.4209569	0	0.00E+00

Down regulated

NAME	SIZE	ES	NES	NOM p-val	FDR q-val
GO_MITOCHONDRIAL_MATRIX	328	-0.2680792	-5.5291944	0	0
GO_ORGANELLE_INNER_MEMBRANE	373	-0.2292796	-5.116826	0	0
GO_RIBOSOMAL_SUBUNIT	165	-0.3340354	-5.0792055	0	0
GO_RIBOSOME	200	-0.2999541	-4.9023514	0	0
GO_MITOCHONDRIAL_PROTEIN_COMPLEX	219	-0.2804582	-4.7518125	0	0
GO_MITOCHONDRIAL_MEMBRANE_PART	177	-0.2659288	-4.061971	0	0
GO_LARGE_RIBOSOMAL_SUBUNIT	102	-0.3275839	-3.8572156	0	0
GO_INNER_MITOCHONDRIAL_MEMBRANE_PROTEIN_COMPLEX	106	-0.3053613	-3.6948695	0	0.00E+00
GO_CYTOSOLIC_RIBOSOME	97	-0.3166761	-3.6027818	0	0.00E+00
GO_BLOOD_MICROPARTICLE	37	-0.4889873	-3.5712862	0	0.00E+00

Supplementary Table 10

Supplementary Table. 10: Gene set enrichment analysis (GSEA) identification of the up- and down- regulated KEGG pathways after early repeated ketamine anesthesia (top 10). Significant changes are marked in red.

Up regulated

NAME	SIZE	ES	NES	NOM p-val	FDR q-val
KEGG_TYPE_II_DIABETES_MELLITUS	31	0.34471616	2.3403354	0.00209205	0.03140269
KEGG_AXON_GUIDANCE	95	0.19418265	2.1839118	0.00197628	0.04430335
KEGG_NEUROACTIVE_LIGAND_RECEPTOR_INTERACTION	80	0.1902526	2.0042784	0.00775194	0.10355631
KEGG_MAPK_SIGNALING_PATHWAY	171	0.12936616	1.9839814	0.0021645	0.08530339
KEGG_BASAL_CELL_CARCINOMA	18	0.37637243	1.9078703	0.00773694	0.10643002
KEGG_ERBB_SIGNALING_PATHWAY	68	0.19968374	1.8984882	0.00578035	0.09261265
KEGG_CHEMOKINE_SIGNALING_PATHWAY	101	0.14946818	1.7269373	0.02788845	0.1892394
KEGG_NOTCH_SIGNALING_PATHWAY	33	0.25704092	1.7133173	0.03082852	0.17824526
KEGG_GLYCOSAMINOGLYCAN_BIOSYNTHESIS_HEPARAN_SULFATE	21	0.28783244	1.5620546	0.05719921	0.3316105
KEGG_CELL_ADHESION_MOLECULES_CAMS	50	0.18794179	1.5493605	0.055666	0.3168493

Down regulated

NAME	SIZE	ES	NES	NOM p-val	FDR q-val
KEGG_RIBOSOME	79	-0.3939749	-4.092171	0	0
KEGG_PARKINSONS_DISEASE	101	-0.2788788	-3.2177384	0	0
KEGG_OXIDATIVE_PHOSPHORYLATION	104	-0.261278	-3.1489658	0	0
KEGG_HUNTINGTONS_DISEASE	138	-0.2236266	-3.0296643	0	0
KEGG_CITRATE_CYCLE_TCA_CYCLE	28	-0.4632101	-2.905669	0	2.73E-04
KEGG_PROTEASOME	38	-0.4035107	-2.8830554	0	2.27E-04
KEGG_VALINE_LEUCINE_AND_ISOLEUCINE_DEGRADATION	35	-0.4058174	-2.8208015	0	3.70E-04
KEGG_GLUTATHIONE_METABOLISM	26	-0.4101099	-2.5194607	0	0.00231003
KEGG_BUTANOATE_METABOLISM	21	-0.4637483	-2.5188985	0	0.00205336
KEGG_SPLICEOSOME	108	-0.2052562	-2.489203	0	0.00196341

Supplementary Table 11

Supplementary Table. 11: Gene set enrichment analysis (GSEA) identification of the up- and down-regulated cell types after early repeated ketamine anesthesia. Significant changes are marked in red.

Up regulated

NAME	SIZE	ES	NES	NOM p-val	FDR q-val
**S1_PYRNEURONS_ZEISEL**	**132**	**0.46433485**	**6.25041**	**0**	**0**
**NEURONS_CAHOY**	**1216**	**0.12935367**	**4.853767**	**0**	**0**
**CA1_PYRNEURONS_ZEISEL**	**273**	**0.12822126**	**2.434659**	**0**	**0.00210648**
**GDT_NKT_T4_MOD56_JOJIC**	**25**	**0.39785957**	**2.340757**	**0**	**0.00316351**
TCELL_MOD18_JOJIC	41	0.19358362	1.4577919	0.09179688	0.22328442
ENDOTHELIAL_ZEISEL	149	0.10238693	1.4139128	0.09657948	0.2196796
ABTCELL_MOD57_JOJIC	30	0.21077402	1.3723031	0.11776859	0.21937726
MICROGLIA_ALBRIGHT	370	0.056979	1.254951	0.21165049	0.30756843
BCELL_MOD33_JOJIC	52	0.1387481	1.185923	0.23863636	0.34671465
INTERNEURONS_ZEISEL	193	0.05944887	0.97519255	0.46168584	0.60588807

Down regulated

NAME	SIZE	ES	NES	NOM p-val	FDR q-val
**ASTROCYTES_CAHOY**	**1346**	**-0.0680018**	**-2.673346**	**0**	**5.56E-04**
**EPENDYMAL_ZEISEL**	**118**	**-0.1709573**	**-2.1323743**	**0.00203252**	**0.00667181**
OLIGODENDROCYTES_ZEISEL	276	-0.0891083	-1.6628462	0.03529412	0.05675643
OLIGODENDROCYTES_CAHOY	1250	-0.0410535	-1.5511676	0.04528302	0.07187642
MURAL_ZEISEL	45	-0.1609735	-1.2989994	0.15678777	0.16048439

Supplementary notes
(Supplementary Note 1-15)

Supplementary note 1

The results of statistical analysis for Figure 1

Fig1_A.LOR_duration_Male Data analysis using R

Boohwi Hong

Package install

Data import

Data structure

```
str(d1)
```

```
## 'data.frame':    60 obs. of  3 variables:
##  $ subject : int  1 2 3 4 5 6 7 8 9 10 ...
##  $ inject  : chr  "1st_inject" "1st_inject" "1st_inject" "1st_inject" ...
##  $ duration: int  520 273 416 0 0 191 412 223 207 526 ...
```

Explorative data analysis with graphics

```
## # A tibble: 5 x 11
##   inject     variable     n   min   max median   iqr  mean    sd    se    ci
##   <chr>      <chr>    <dbl> <dbl> <dbl>  <dbl> <dbl> <dbl> <dbl> <dbl> <dbl>
## 1 1st_inject duration    12     0   526    215 413   231.  203.   58.7 129.
## 2 2nd_inject duration    12     0   360      0  69.5  79.2 144.   41.7  91.7
## 3 3rd_inject duration    12     0   370      0   0    30.8 107.   30.8  67.9
## 4 4th_inject duration    12     0   190      0   0    15.8  54.8  15.8  34.8
## 5 5th_inject duration    12     0     0      0   0     0     0     0     0
```

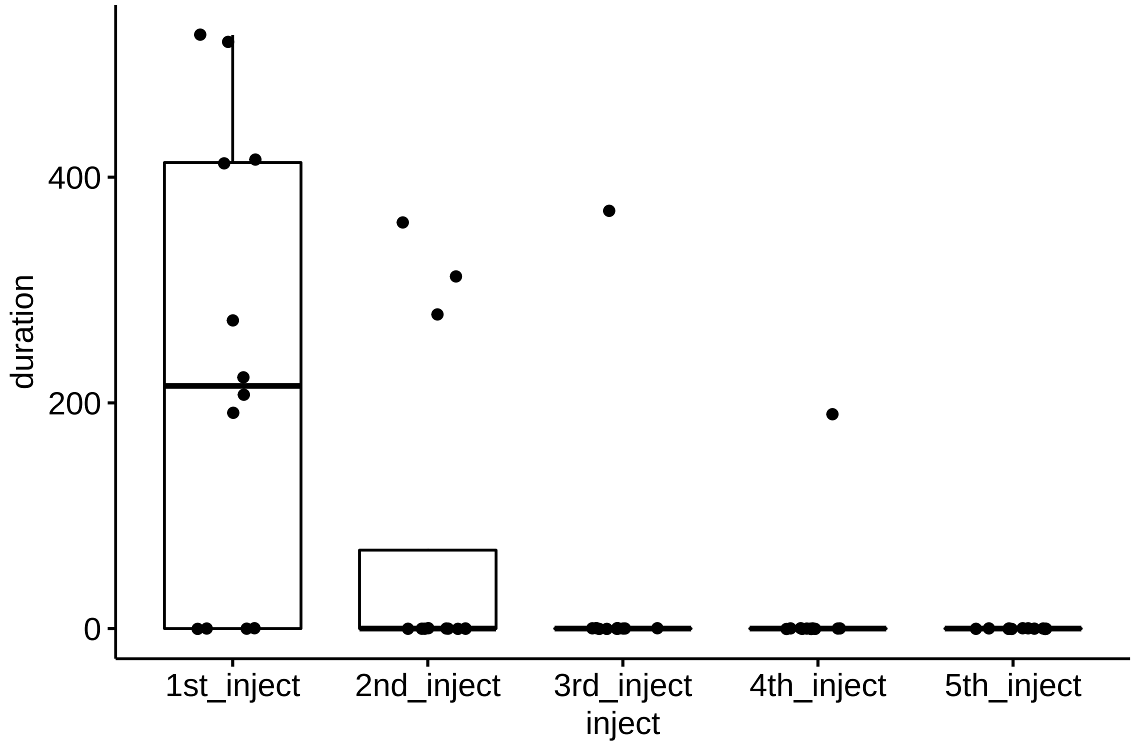

Model fit

```
## # A tibble: 1 x 6
##   .y.          n statistic    df        p method
## * <chr>    <int>     <dbl> <dbl>    <dbl> <chr>
## 1 duration    12      18.7     4 0.000909 Friedman test
```

Effect size

```
## # A tibble: 1 x 5
##   .y.          n effsize method    magnitude
## * <chr>    <int>   <dbl> <chr>     <ord>
## 1 duration    12   0.389 Kendall W moderate
```

Multiple pairwise-comparisons

```
## # A tibble: 10 x 9
##    .y.      group1     group2        n1    n2 statistic     p p.adj p.adj.signif
##  * <chr>    <chr>      <chr>      <int> <int>     <dbl> <dbl> <dbl> <chr>
##  1 duration 1st_inject 2nd_inject    12    12        38 0.076 0.756 ns
##  2 duration 1st_inject 3rd_inject    12    12        40 0.044 0.44  ns
##  3 duration 1st_inject 4th_inject    12    12        36 0.014 0.143 ns
##  4 duration 1st_inject 5th_inject    12    12        36 0.014 0.143 ns
##  5 duration 2nd_inject 3rd_inject    12    12         6 0.855 1     ns
```

```
##  6 duration 2nd_inject 4th_inject    12    12         9 0.201 1     ns
##  7 duration 2nd_inject 5th_inject    12    12         6 0.181 1     ns
##  8 duration 3rd_inject 4th_inject    12    12         2 1     1     ns
##  9 duration 3rd_inject 5th_inject    12    12         1 1     1     ns
## 10 duration 4th_inject 5th_inject    12    12         1 1     1     ns
```

Interpretation of result

```
## 1. The duration was statistically significantly different at the different time points
##  p = 0.001
```

Fig1_B.Weight_Male Data analysis using R

Boohwi Hong

Package install

Data import

Data structure

```
str(d1)
```

```
## 'data.frame':    135 obs. of  4 variables:
##  $ subject: int  1 2 3 4 5 6 7 8 9 10 ...
##  $ group  : chr  "cont" "cont" "cont" "cont" ...
##  $ inject : chr  "1st_inject" "1st_inject" "1st_inject" "1st_inject" ...
##  $ weight : num  8.5 8.2 9.3 8 7.5 8.3 7.6 8.1 8.3 8.6 ...
```

Explorative data analysis with graphics

```
##     group     inject  n Mean Conf.level Percentile.lower Percentile.upper
## 1  anesth 1st_inject 12 8.31       0.95             8.07             8.54
## 2  anesth 2nd_inject 12 8.36       0.95             8.10             8.60
## 3  anesth 3rd_inject 12 8.38       0.95             8.02             8.72
## 4  anesth 4th_inject 12 8.73       0.95             8.25             9.17
## 5  anesth 5th_inject 12 8.90       0.95             8.43             9.34
## 6    cont 1st_inject 15 8.16       0.95             7.94             8.39
## 7    cont 2nd_inject 15 8.25       0.95             8.01             8.50
## 8    cont 3rd_inject 15 8.48       0.95             8.27             8.69
## 9    cont 4th_inject 15 8.69       0.95             8.49             8.90
## 10   cont 5th_inject 15 8.84       0.95             8.59             9.11
```

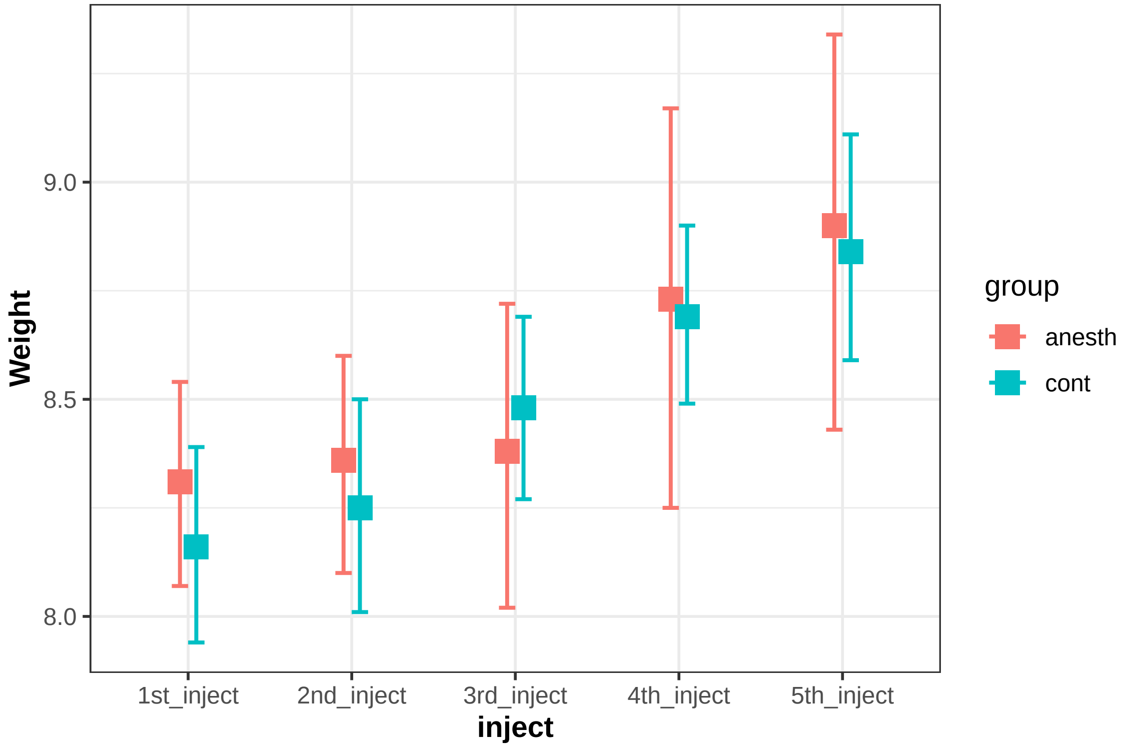

Model fit

```
##
## Error: subject
##       Df Sum Sq Mean Sq
## group  1  1.726   1.726
##
## Error: subject:inject
##        Df Sum Sq Mean Sq
## inject  4  4.278    1.07
##
## Error: Within
##               Df Sum Sq Mean Sq F value   Pr(>F)
## group          1  7.895   7.895  32.804 7.69e-08 ***
## inject         4  4.508   1.127   4.683  0.00151 **
## group:inject   4  1.831   0.458   1.901  0.11460
## Residuals    120 28.882   0.241
## ---
## Signif. codes:  0 '***' 0.001 '**' 0.01 '*' 0.05 '.' 0.1 ' ' 1
```

Interpretation of result

```
## 1. Difference exist between groups
##  p = 0.000

## 2. Difference exist between measurement points
##  p = 0.002

## 3. Significant interaction do not exist between groups and measurement points
##  p = 0.115
```

Fig1_E.OFT_distance_Male Data analysis using R

Boohwi Hong

Package install

Data import

Data structure

```
str(d1)
```

```
## 'data.frame':    23 obs. of  3 variables:
##  $ subject : int  1 2 3 4 5 6 7 8 9 10 ...
##  $ group   : chr  "cont_saline" "cont_saline" "cont_saline" "cont_saline" ...
##  $ distance: num  100.2 123 106 78.8 128.9 ...
```

Explorative data analysis with graphics

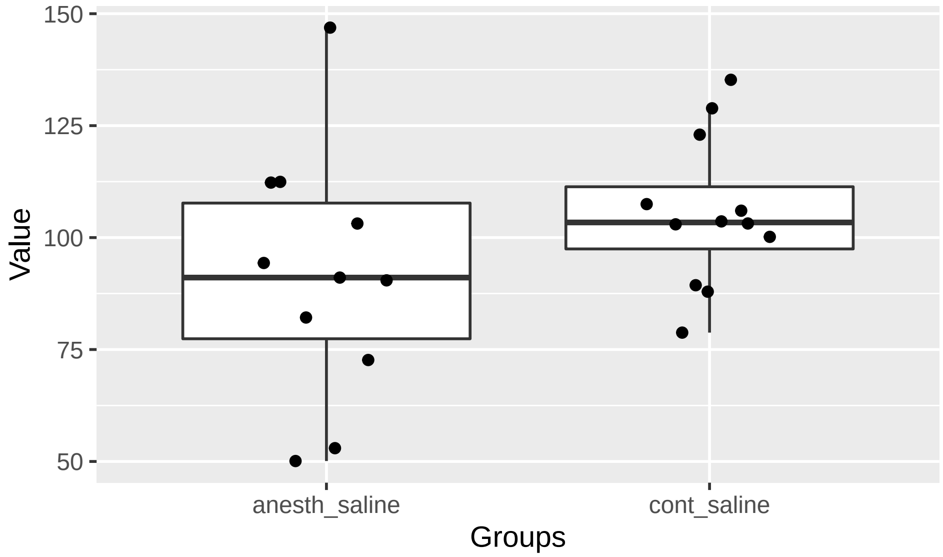

Easystat function developed by S. Park (available at https://rpubs.com/goodlebang)

Statistical Result

```
easystat(d1)
```

```
## 1. Normality assumption test by Shapiro_Wilk test is
##  p = 0.666
##  Normality assumption was not rejected
## 2. Equal variance test by Bartlett test is
##  p = 0.112
##  Equal variance assumption was not rejected
## 3. The result of anova is
##  p = 0.1577
##  A statistically significant difference do not exist between groups
```

Fig1_F.OFT_distance_Male Data analysis using R

Boohwi Hong

Package install

Data import

Data structure

```
str(d1)
```

```
## 'data.frame':    48 obs. of  3 variables:
##  $ subject : int  1 2 3 4 5 6 7 8 9 10 ...
##  $ group   : chr  "cont_1st" "cont_1st" "cont_1st" "cont_1st" ...
##  $ distance: num  121 141 219 126 160 ...
```

Explorative data analysis with graphics

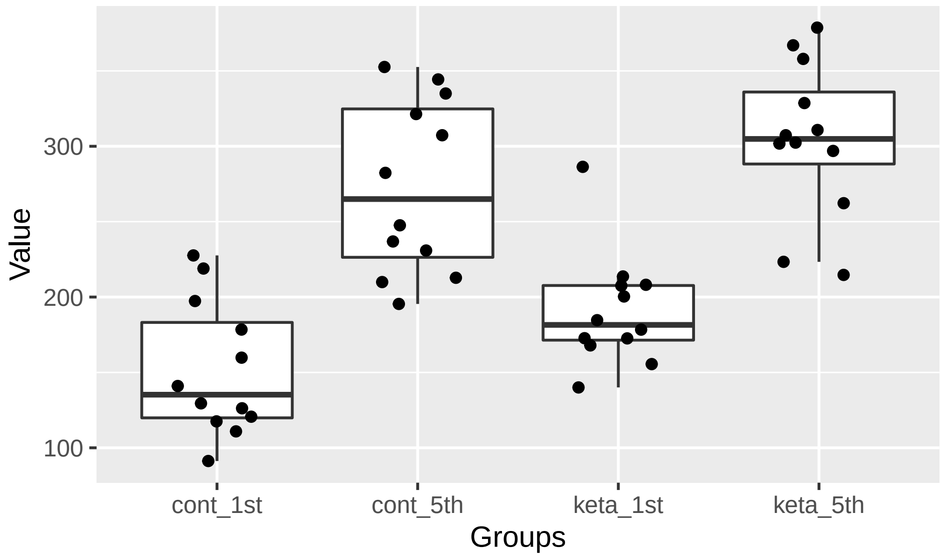

Easystat function developed by S. Park (available at https://rpubs.com/goodlebang)

Statistical Result

```
easystat(d1)
```

```
## 1. Normality assumption test by Shapiro_Wilk test is
##  p = 0.43
##  Normality assumption was not rejected
## 2. Equal variance test by Bartlett test is
##  p = 0.556
##  Equal variance assumption was not rejected
## 3. The result of anova is
##  p = 0.000
##  A statistically significant difference exist between groups
```

```
##   Tukey multiple comparisons of means
##     95% family-wise confidence level
##
## Fit: aov(formula = d1[, 3] ~ d1[, 2], data = d1)
##
## $`d1[, 2]`
##                         diff        lwr       upr     p adj
## cont_5th-cont_1st 121.45438   68.71147 174.19729 0.0000012
## keta_1st-cont_1st  39.06347  -13.67944  91.80638 0.2119723
## keta_5th-cont_1st 152.73480   99.99189 205.47771 0.0000000
## keta_1st-cont_5th -82.39092 -135.13383 -29.64801 0.0007838
## keta_5th-cont_5th  31.28042  -21.46249  84.02333 0.3983189
## keta_5th-keta_1st 113.67133   60.92842 166.41424 0.0000045
```

Fig1_G.OFT_distance_Male Data analysis using R

Boohwi Hong

Package install

Data import

Data structure

```
str(d1)
```

```
## 'data.frame':    120 obs. of  4 variables:
##  $ subject : int  13 14 15 16 17 18 19 20 21 22 ...
##  $ group   : chr  "cont_keta" "cont_keta" "cont_keta" "cont_keta" ...
##  $ trial   : int  1 1 1 1 1 1 1 1 1 1 ...
##  $ distance: num  121 141 219 126 160 ...
```

Explorative data analysis with graphics

```
##          group trial  n Mean Conf.level Percentile.lower Percentile.upper
## 1  anesth_keta     1 12  191       0.95              172              213
## 2  anesth_keta     2 12  222       0.95              205              239
## 3  anesth_keta     3 12  295       0.95              267              323
## 4  anesth_keta     4 12  287       0.95              261              314
## 5  anesth_keta     5 12  304       0.95              276              331
## 6    cont_keta     1 12  152       0.95              128              176
## 7    cont_keta     2 12  188       0.95              156              219
## 8    cont_keta     3 12  229       0.95              195              270
## 9    cont_keta     4 12  266       0.95              233              303
## 10   cont_keta     5 12  273       0.95              242              304
```

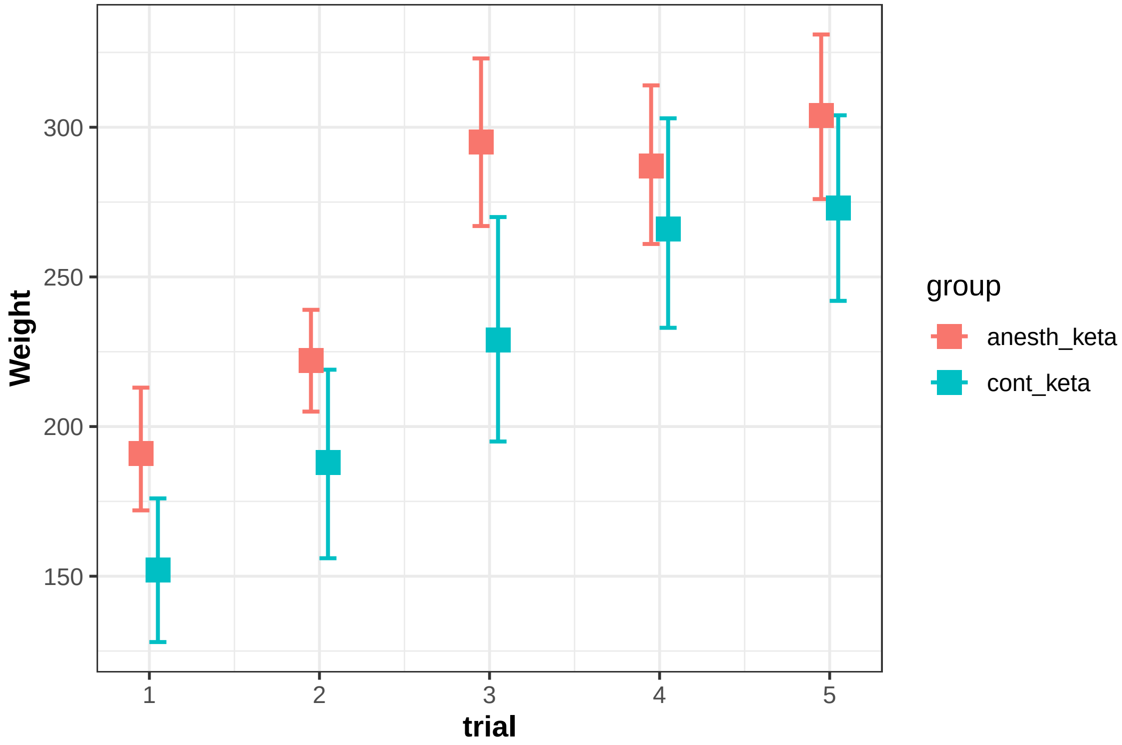

Model fit

```
##
## Error: subject
##       Df Sum Sq Mean Sq
## group  1  31388   31388
##
## Error: subject:trial
##       Df Sum Sq Mean Sq
## trial  1 190087  190087
##
## Error: Within
##              Df Sum Sq Mean Sq F value  Pr(>F)
## group         1  19660   19660   6.873 0.00995 **
## trial         1  36058   36058  12.605 0.00056 ***
## group:trial   1    868     868   0.303 0.58292
## Residuals   114 326100    2861
## ---
## Signif. codes:  0 '***' 0.001 '**' 0.01 '*' 0.05 '.' 0.1 ' ' 1
```

Interpretation of result

```
## 1. Difference exist between groups
##  p = 0.010

## 2. Difference exist between measurement points
##  p = 0.001

## 3. Significant interaction do not exist between groups and measurement points
##  p = 0.583
```

Supplementary Note 2

The results of statistical analysis for Figure 2

Fig2_B.keta-CPP_Anesth_Male Data analysis using R

Boohwi Hong

Statistical Result

```
## Present data is ** Fig2_B.keta-CPP_Anesth_Male.csv **
##
## **  Data structure  **
## 'data.frame':    54 obs. of  3 variables:
##  $ subject : int  1 2 3 4 5 6 7 8 9 10 ...
##  $ group   : chr  "pre" "pre" "pre" "pre" ...
##  $ duration: num  289 295 294 191 274 ...
##
## ** Explorative data analysis with graphics**
```

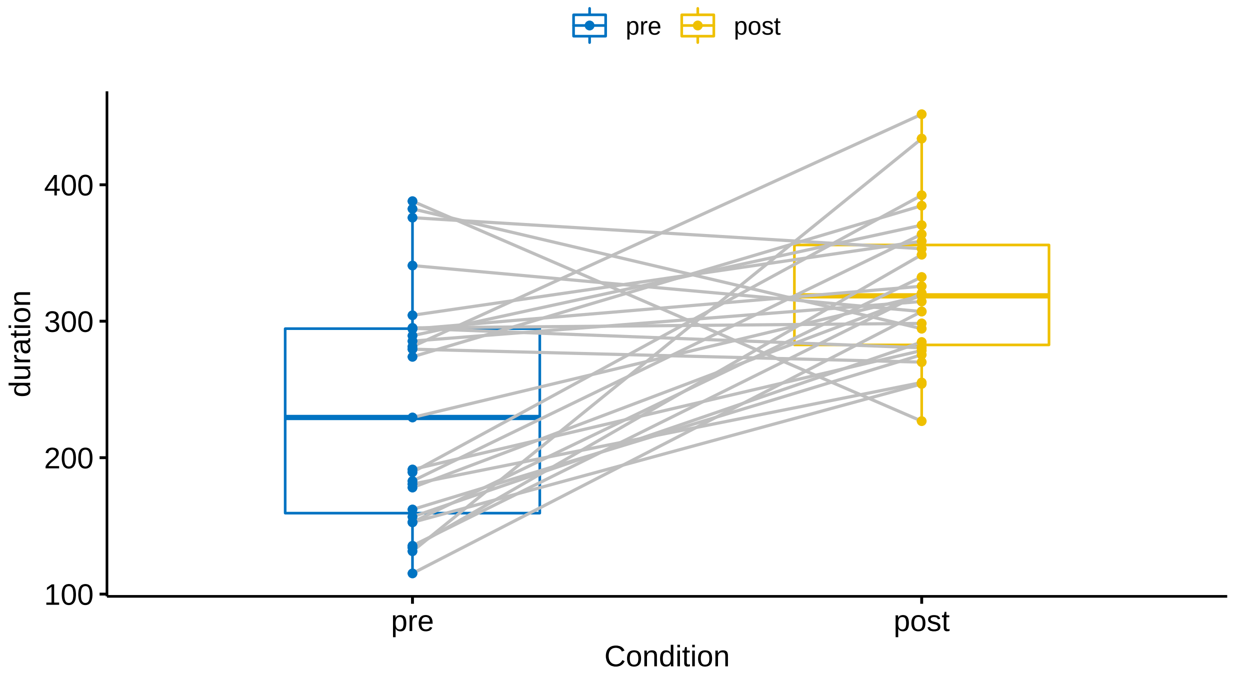


```
## 1. Normality assumption test by Shapiro_Wilk test is
##  p = 0.114
##  Normality assumption was not rejected
## 2. The result of paired t-test is
##  p = 0.000
##  A statistically significant difference exist between groups
```

Fig2_B.keta-CPP_Control_MaleData analysis using R

Boohwi Hong

Statistical Result

```
## Present data is ** Fig2_B.keta-CPP_Control_Male.csv **
##
## **  Data structure  **
## 'data.frame':    52 obs. of  3 variables:
##  $ subject : int  1 2 3 4 5 6 7 8 9 10 ...
##  $ group   : chr  "pre" "pre" "pre" "pre" ...
##  $ duration: num  237 230 264 195 267 ...
##
## ** Explorative data analysis with graphics**
```

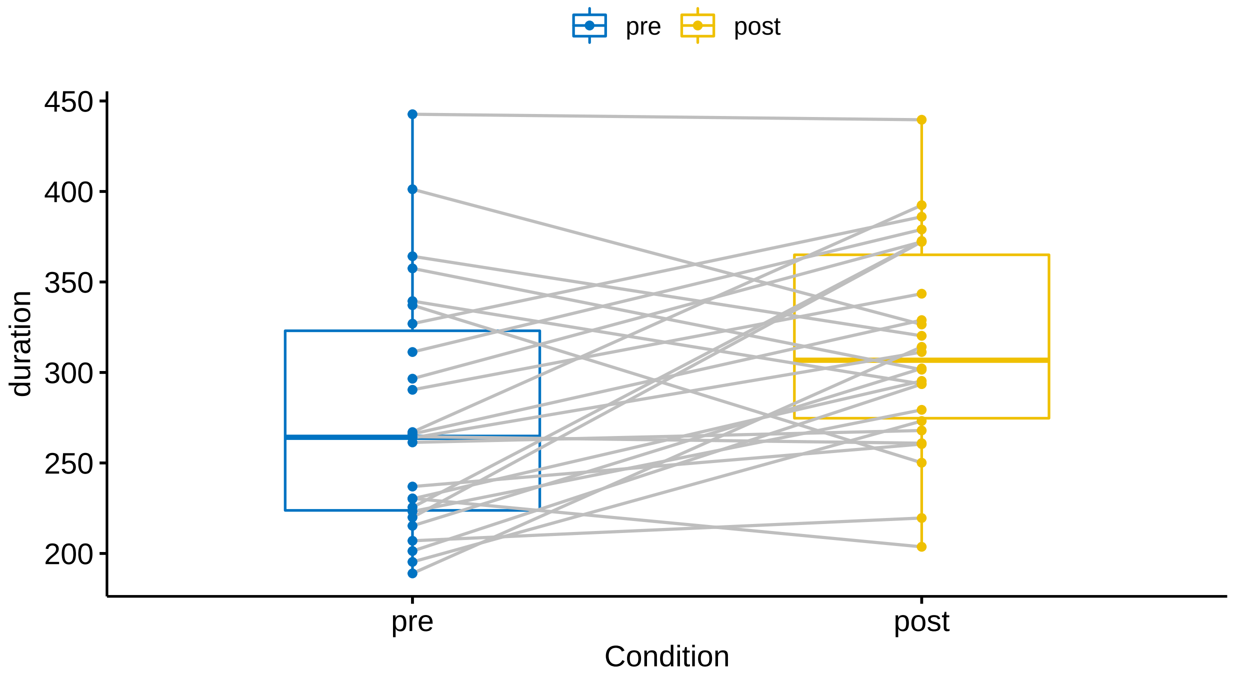


```
## 1. Normality assumption test by Shapiro_Wilk test is
##  p = 0.135
##  Normality assumption was not rejected
## 2. The result of paired t-test is
##  p = 0.007
##  A statistically significant difference exist between groups
```

Fig2_C.keta-CPP_CPPscore_MaleData analysis using R

Boohwi Hong

```
## Present data is ** Fig2_C.keta-CPP_CPPscore_Male.csv **
##
## **  Data structure  **
## 'data.frame':    53 obs. of  3 variables:
##  $ subject   : int  1 2 3 4 5 6 7 8 9 10 ...
##  $ group     : chr  "control" "control" "control" "control" ...
##  $ difference: num  23.5 -26.8 47.4 77.8 125.4 ...
##
## ** Explorative data analysis with graphics**
```

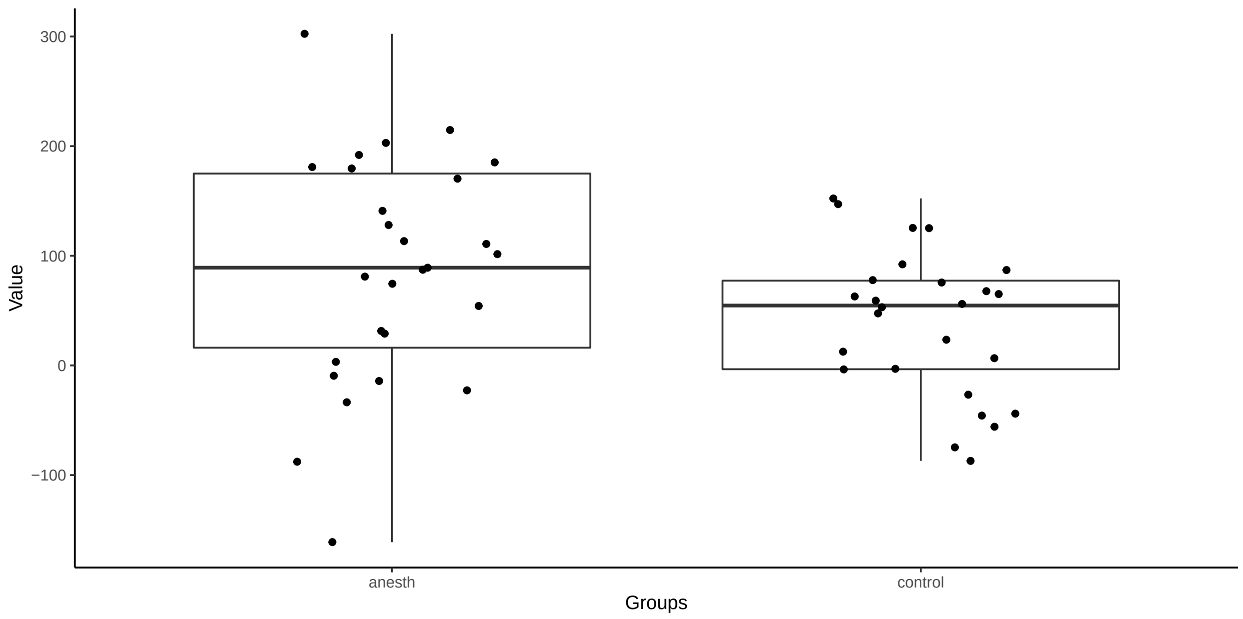


```
## 1. Normality assumption test by Shapiro_Wilk test is
##  p = 0.672
##  Normality assumption was not rejected
## 2. Equal variance test by Bartlett test is
##  p = 0.032
##  Equal variance assumption was rejected
## 3. The result of Welch ANOVA is
##  p = 0.049
##  A statistically significant difference exist between groups
##
##   Tukey multiple comparisons of means
##     95% family-wise confidence level
##
```

```
## Fit: aov(formula = d1[, 3] ~ d1[, 2])
##
## $`d1[, 2]`
##                    diff       lwr         upr     p adj
## control-anesth -48.47184 -96.86342 -0.08025694 0.0496353
```

Fig2_D.nicotine-CPP_Anesth_MaleData analysis using R

Boohwi Hong

Statistical Result

```
## Present data is ** Fig2_D.nicotine-CPP_Anesth_Male.csv **
##
## **  Data structure  **
## 'data.frame':    32 obs. of  3 variables:
##  $ subject : int  1 2 3 4 5 6 7 8 9 10 ...
##  $ group   : chr  "pre" "pre" "pre" "pre" ...
##  $ duration: num  287 285 249 340 318 ...
##
## ** Explorative data analysis with graphics**
```

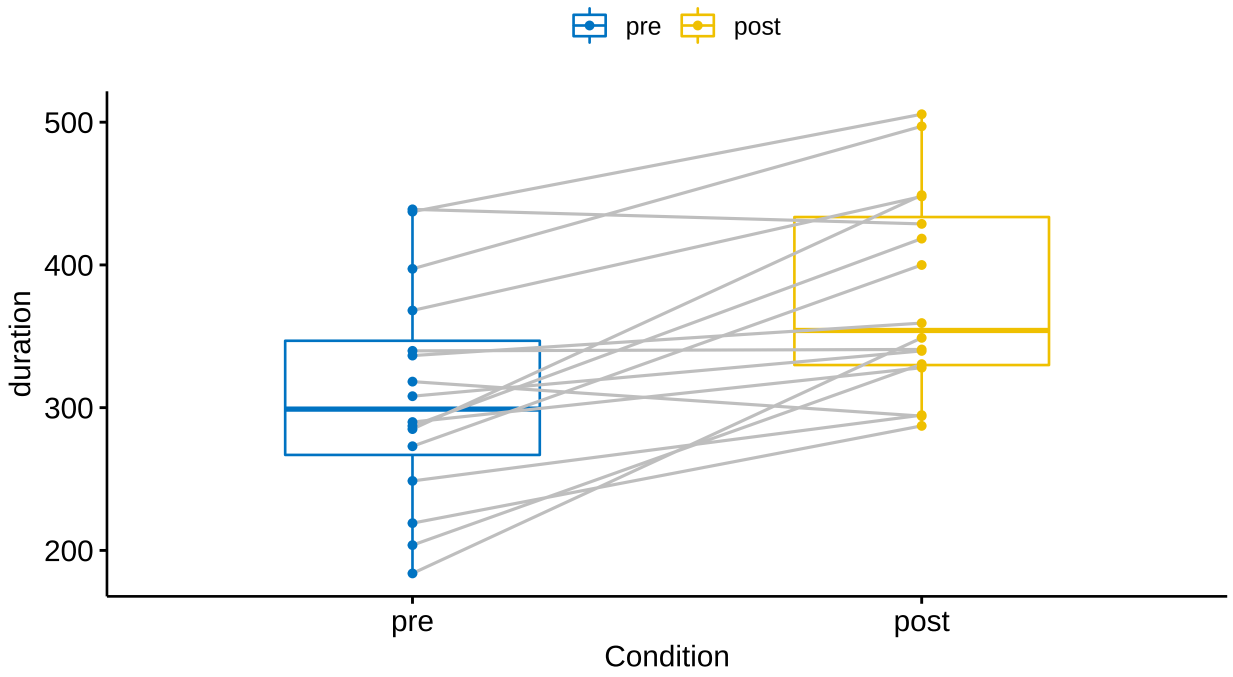


```
## 1. Normality assumption test by Shapiro_Wilk test is
##  p = 0.251
##  Normality assumption was not rejected
## 2. The result of paired t-test is
##  p = 0.000
##  A statistically significant difference exist between groups
```

Fig2_D.nicotine-CPP_Control_MaleData analysis using R

Boohwi Hong

Statistical Result

```
## Present data is ** Fig2_D.nicotine-CPP_Control_Male.csv **
##
## **  Data structure  **
## 'data.frame':    32 obs. of  3 variables:
##  $ subject : int  1 2 3 4 5 6 7 8 9 10 ...
##  $ group   : chr  "pre" "pre" "pre" "pre" ...
##  $ duration: num  352 292 398 317 267 ...
##
## ** Explorative data analysis with graphics**
```

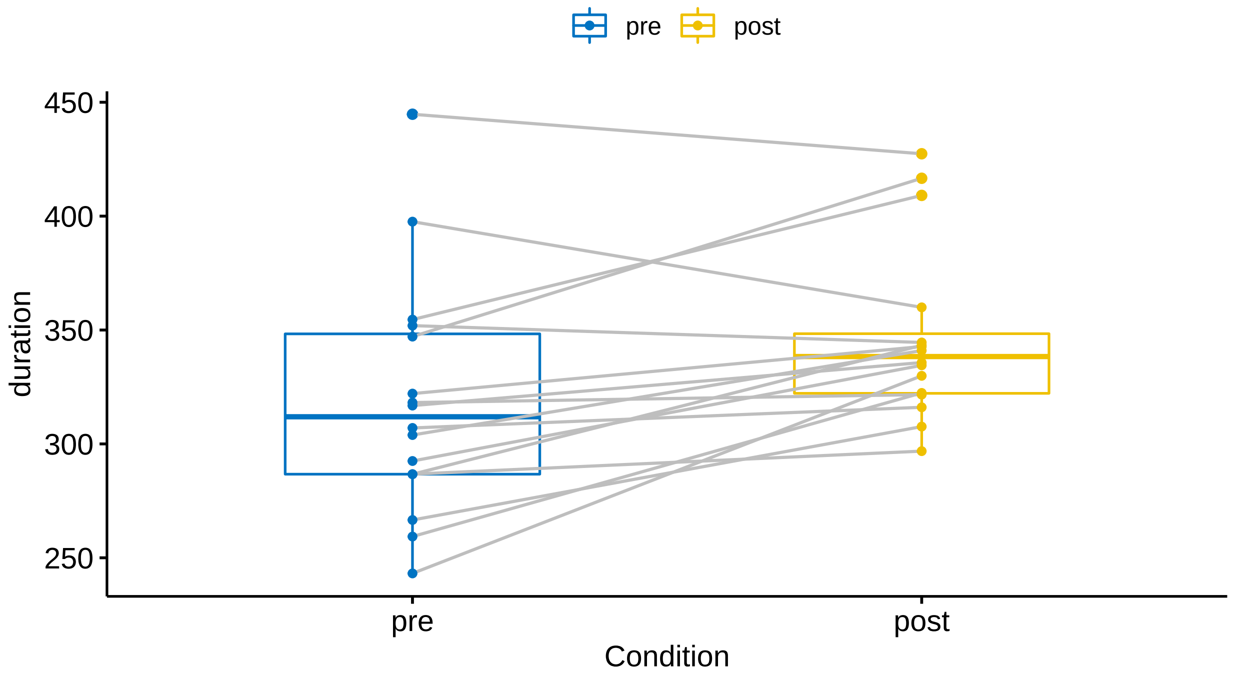


```
## 1. Normality assumption test by Shapiro_Wilk test is
##  p = 0.032
##  Normality assumption was rejected
## 2. The result of Wilcoxon test is
##  p = 0.056
##  A statistically significant difference do not exist between groups
```

Fig2_E.nicotine-CPP_CPPscore_MaleData analysis using R

Boohwi Hong

```
## Present data is ** Fig2_E.nicotine-CPP_CPPscore_Male.csv **
##
## **  Data structure  **
## 'data.frame':    32 obs. of  3 variables:
##  $ subject   : int  1 2 3 4 5 6 7 8 9 10 ...
##  $ group     : chr  "control" "control" "control" "control" ...
##  $ difference: num  -7.39 42.01 -37.62 18.89 41.01 ...
##
## ** Explorative data analysis with graphics**
```

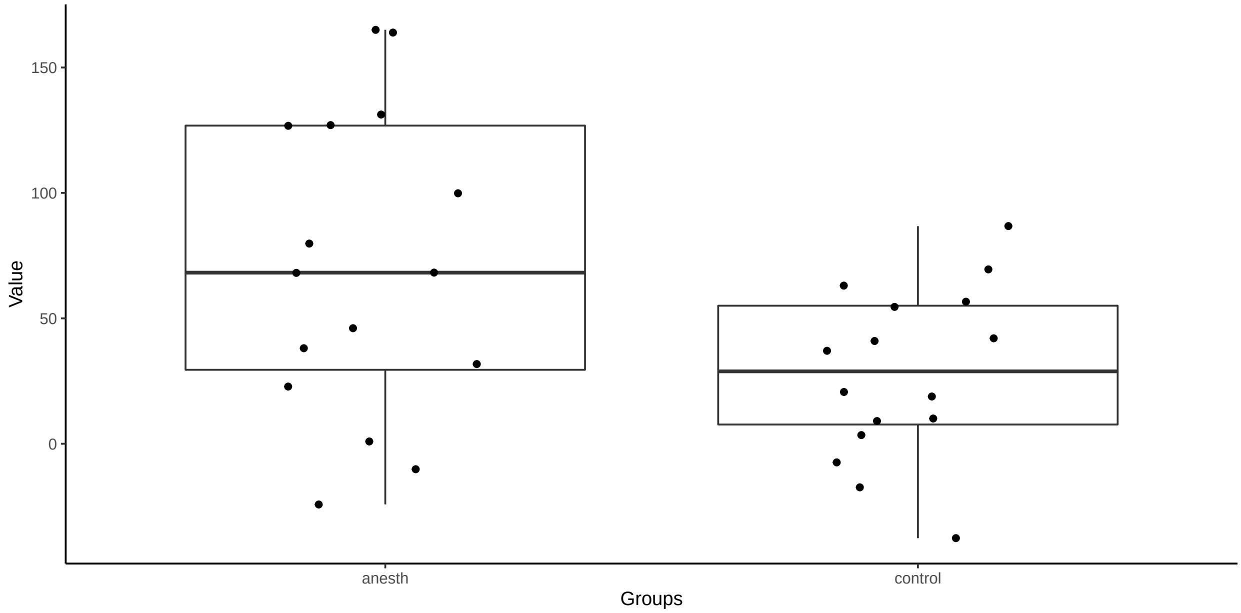


```
## 1. Normality assumption test by Shapiro_Wilk test is
##  p = 0.937
##  Normality assumption was not rejected
## 2. Equal variance test by Bartlett test is
##  p = 0.034
##  Equal variance assumption was rejected
## 3. The result of Welch ANOVA is
##  p = 0.021
##  A statistically significant difference exist between groups
##
##   Tukey multiple comparisons of means
##     95% family-wise confidence level
##
```

```
## Fit: aov(formula = d1[, 3] ~ d1[, 2])
##
## $`d1[, 2]`
##                     diff       lwr       upr     p adj
## control-anesth -42.81144 -78.13698 -7.485899 0.0191947
```

Supplementary Note 3

The results of statistical
analysis for Figure 3

Fig3_B.keta-CPP_anesth_male(Adult)Data analysis using R

Boohwi Hong

Statistical Result

```
## Present data is ** Fig3_B.keta-CPP_anesth_male(Adult)xlsx.csv ** ##
## **  Data structure  **
## 'data.frame':    50 obs. of  3 variables:
##$  ubject: int 1 2 3 4 5 6 7 8 9 10 ...
##  $ group     : chr  "pre" "pre" "pre" "pre" ...
##  $ duration  : num  396 242 353 339 290 ...
##
## ** Explorative data analysis with graphics**
```

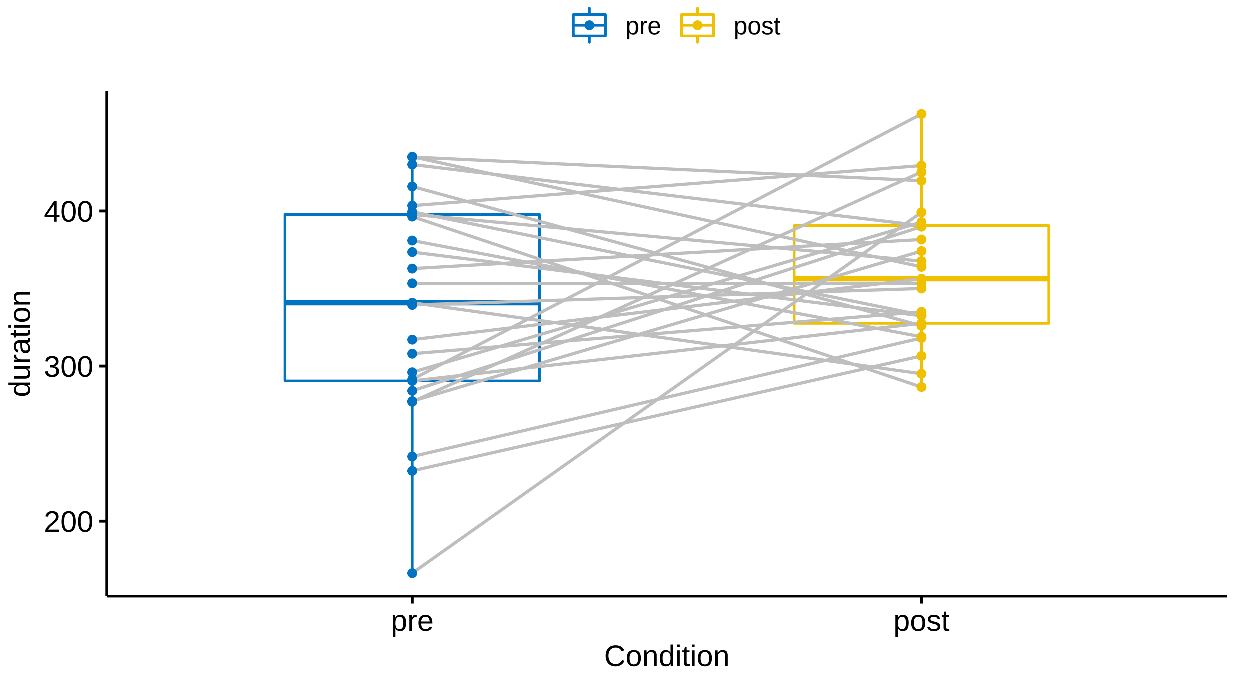


```
## 1. Normality assumption test by Shapiro_Wilk test is
##  p = 0.357
##  Normality assumption was not rejected
## 2. The result of paired t-test is
##  p = 0.183
##  A statistically significant difference do not exist between groups
```

Fig3_B.keta-CPP_control_male(Adult)Data analysis using R

Boohwi Hong

Statistical Result

```
## Present data is ** Fig3_B.keta-CPP_control_male(Adult)xlsx.csv **
##
## **  Data structure  **
## 'data.frame':    52 obs. of  3 variables:
##$  ubject: int 1 2 3 4 5 6 7 8 9 10 ...
##  $ group     : chr  "pre" "pre" "pre" "pre" ...
##  $ duration  : num  263 294 330 276 351 ...
##
## ** Explorative data analysis with graphics**
```

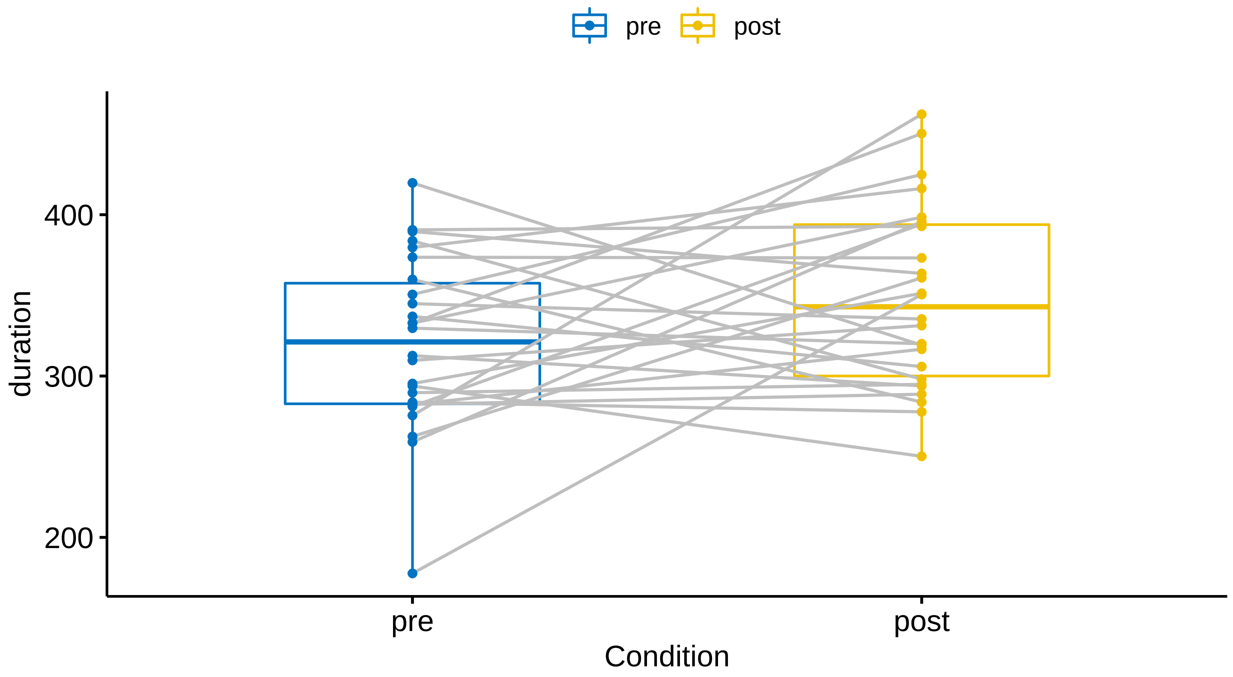


```
## 1. Normality assumption test by Shapiro_Wilk test is
##  p = 0.572
##  Normality assumption was not rejected
## 2. The result of paired t-test is
##  p = 0.074
##  A statistically significant difference do not exist between groups
```

Fig3_C. keta-CPP_CPPscore_male(Adult)Data analysis using R

Boohwi Hong

```
## Present data is ** Fig3_C. keta-CPP_CPPscore_male(Adult)xlsx.csv ** ##
## **  Data structure  **
## 'data.frame':    51 obs. of  3 variables:

##  $  ubject: int  1 2 3 4 5 6 7 8 9 10 ...
##  $ group     : chr  "control" "control" "control" "control" ...
##  $ difference: num  98.33 -43.58 -9.66 186.68 74.31 ...
##
## ** Explorative data analysis with graphics**
```

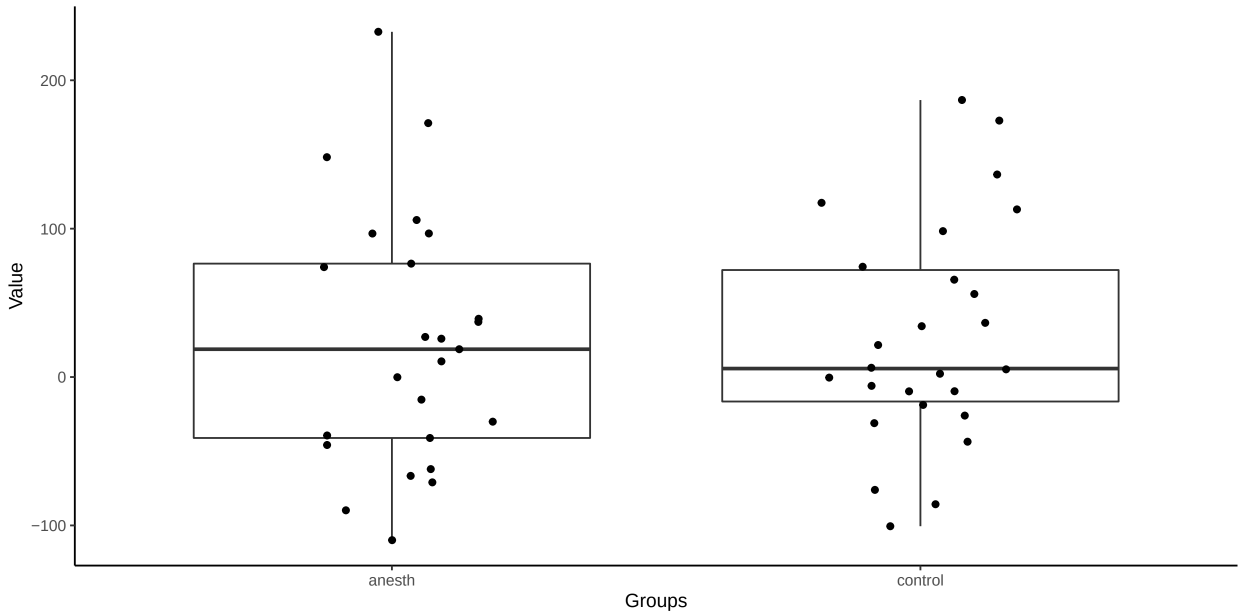


```
## 1. Normality assumption test by Shapiro_Wilk test is
##  p = 0.223
##  Normality assumption was not rejected
## 2. Equal variance test by Bartlett test is
##  p = 0.530
##  Equal variance assumption was not rejected
## 3. The result of anova is
##  p = 0.858
##  A statistically significant difference do not exist between groups
##
```

Fig3_D.nico-CPP_anesth_Male(Adult)Data analysis using R

Boohwi Hong

Statistical Result

```
## Present data is ** Fig3_D.nico-CPP_anesth_Male(Adult).csv ** ##
## **  Data structure  **
## 'data.frame':    34 obs. of  3 variables:
## $  ubjectint 1 2 3 4 5 6 7 8 9 10 ...
##  $ group     : chr  "pre" "pre" "pre" "pre" ...
##  $ duration  : num  332 387 339 289 315 ...
##
## ** Explorative data analysis with graphics**
```

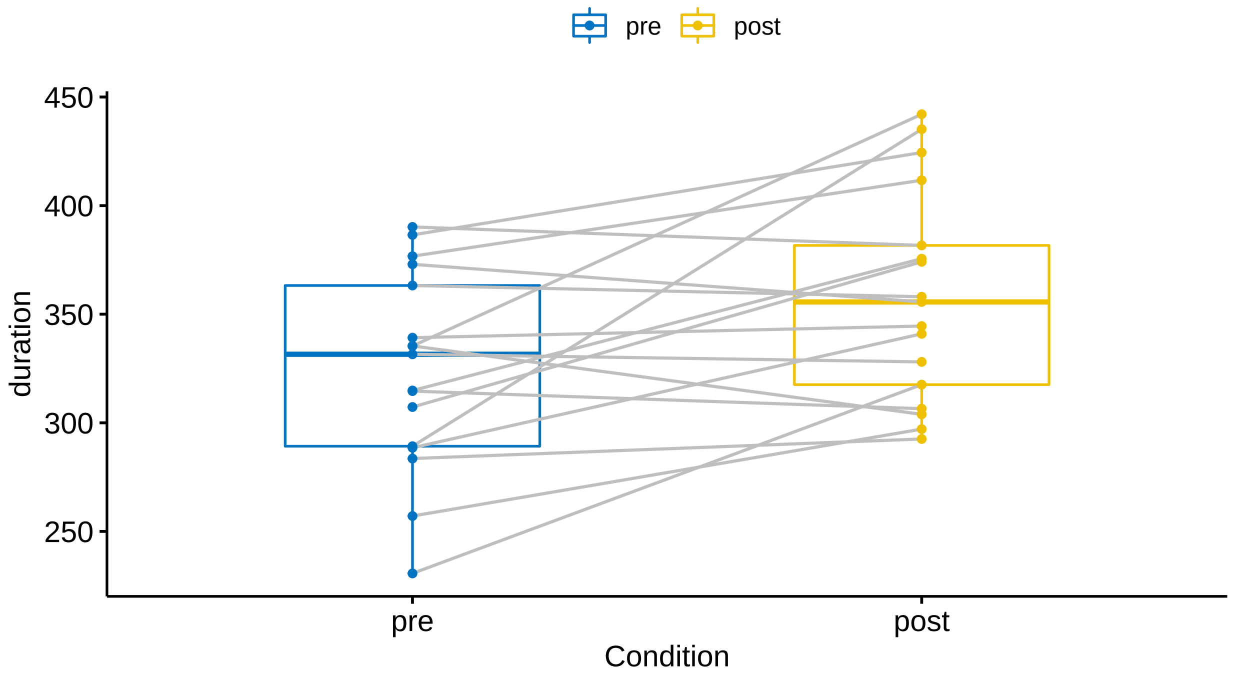


```
## 1. Normality assumption test by Shapiro_Wilk test is
##  p = 0.608
##  Normality assumption was not rejected
## 2. The result of paired t-test is
##  p = 0.011
##  A statistically significant difference exist between groups
```

Fig3_D.nico-CPP_control_Male(Adult)Data analysis using R

Boohwi Hong

Statistical Result

```
## Present data is ** Fig3_D.nico-CPP_control_Male(Adult).csv ** ##
## **  Data structure  **
## 'data.frame':    36 obs. of  3 variables:
##$  ubjectint 1 2 3 4 5 6 7 8 9 10 . . .
##  $ group     : chr  "pre" "pre" "pre" "pre" ...
##  $ duration  : num  207 389 224 352 226 ...
##
## ** Explorative data analysis with graphics**
```

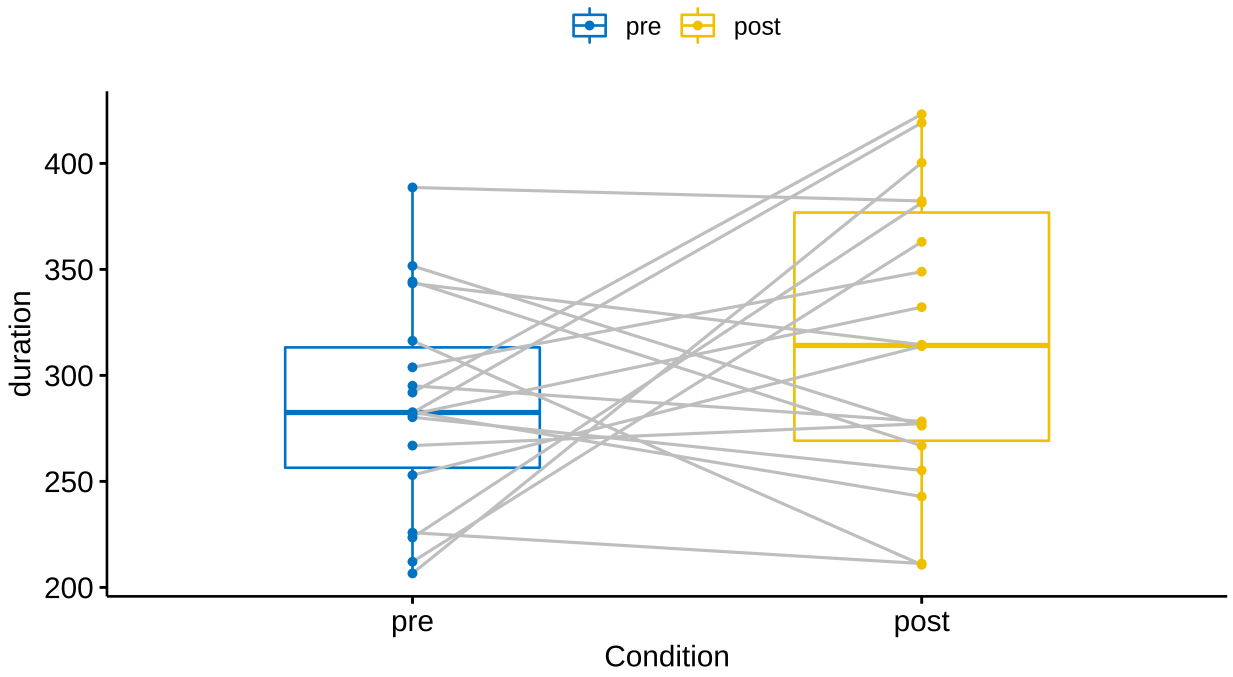


```
## 1. Normality assumption test by Shapiro_Wilk test is
##  p = 0.385
##  Normality assumption was not rejected
## 2. The result of paired t-test is
##  p = 0.172
##  A statistically significant difference do not exist between groups
```

Fig3_E.nico-CPP_CPPscore_Male(Adult)Data analysis using R

Boohwi Hong

```
## Present data is ** Fig3_E.nico-CPP_CPPscore_Male(Adult).csv ** ##
## **  Data structure  **

## 'data.frame':    35 obs. of  3 variables:
##  $  ubject: int  1 2 3 4 5 6 7 8 9 10 ...
##  $ group     : chr  "control" "control" "control" "control" ...
##  $ difference: num  193.72 -6.35 157.92 -75.37 -14.58 ...
##
## ** Explorative data analysis with graphics**
```

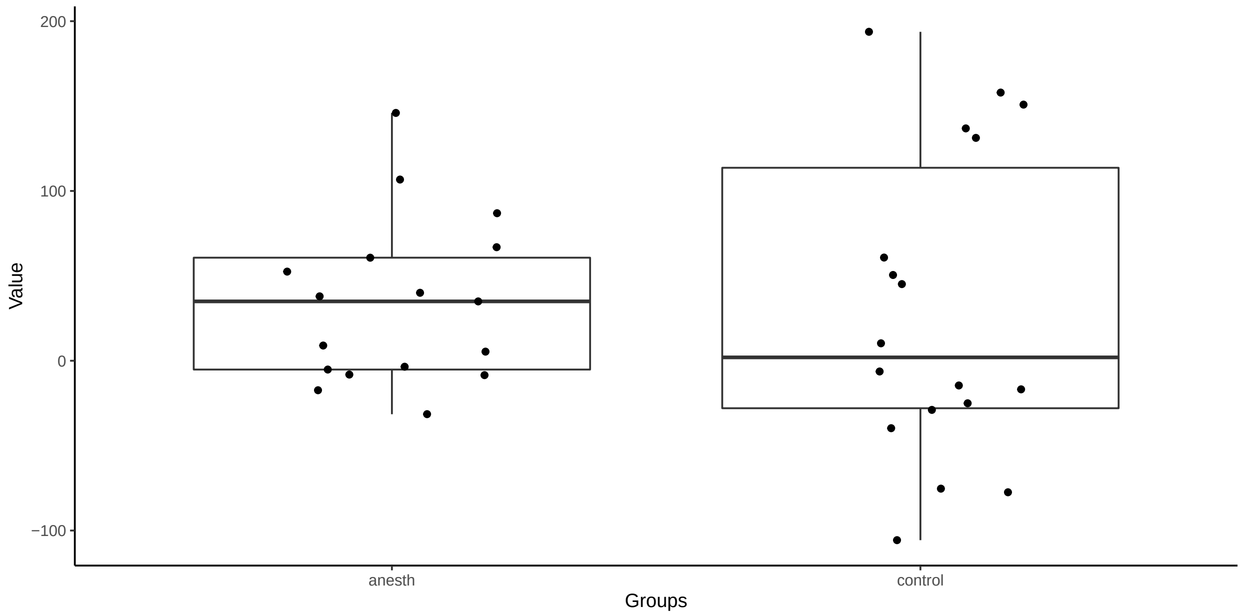


```
## 1. Normality assumption test by Shapiro_Wilk test is
##  p = 0.199
##  Normality assumption was not rejected
## 2. Equal variance test by Bartlett test is
##  p = 0.016
##  Equal variance assumption was rejected
## 3. The result of Welch ANOVA is
##  p = 0.894
##  A statistically significant difference do not exist between groups
##
```

Supplementary Note 4

The results of statistical analysis for Figure 4

Data analysis using R

Boohwi Hong

```
## Present data is ** Fig4_C.action_potential_20_Male.csv **
##
## **  Data structure  **
## 'data.frame':    13 obs. of  4 variables:
##  $  ubject      : int  1 2 3 4 5 6 7 8 9 10 ...
##  $ group           : chr  "control" "control" "control" "control" ...
##  $ input.current   : int  20 20 20 20 20 20 20 20 20 20 ...
##  $ action.potential: num  0 0 0 3 0 0.4 0 0 0 0.8 ...
##
## ** Explorative data analysis with graphics**
```

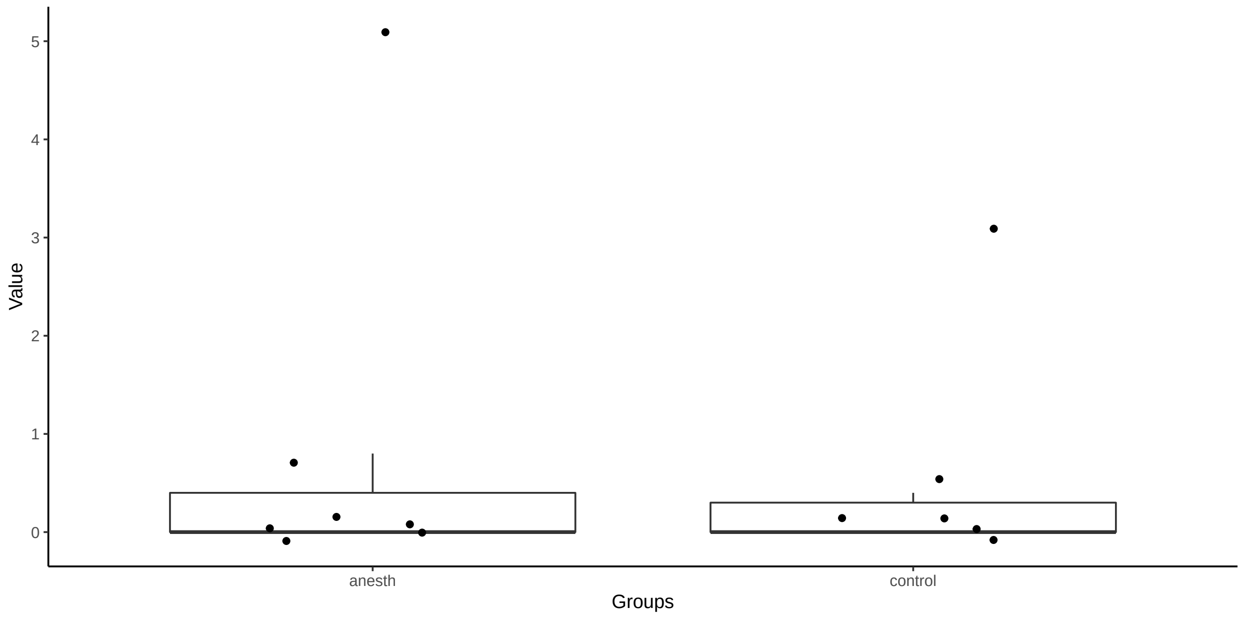


```
## 1. Normality assumption test by Shapiro_Wilk test is
##  p = 0.000
##  Normality assumption was rejected
## 2. The result of Kruskall_Wallis test:
##  p = 1.000
##  A statistically significant difference do not exist between groups
##
```

Data analysis using R

Boohwi Hong

```
## Present data is ** Fig4_C.action_potential_30_Male.csv **
##
## **  Data structure  **
## 'data.frame':    13 obs. of  4 variables:
##  $  ubject      : int  1 2 3 4 5 6 7 8 9 10 ...
##  $ group           : chr  "control" "control" "control" "control" ...
##  $ input.current   : int  30 30 30 30 30 30 30 30 30 30 ...
##  $ action.potential: num  0 0 0 6 1.67 ...
##
## ** Explorative data analysis with graphics**
```

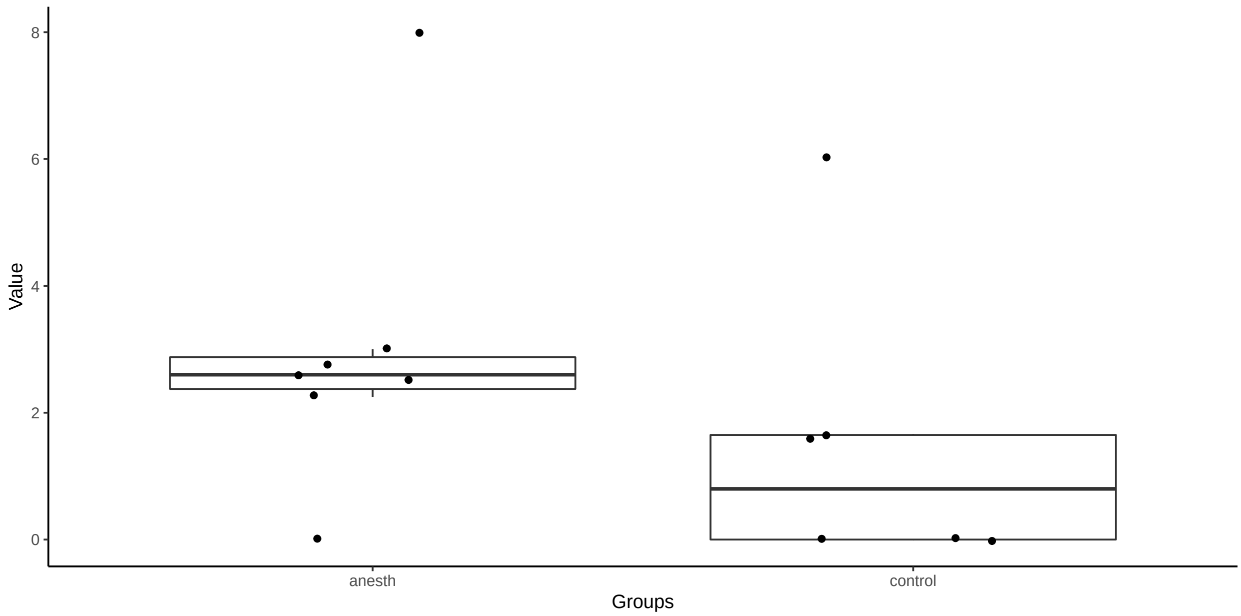


```
## 1. Normality assumption test by Shapiro_Wilk test is
##  p = 0.007
##  Normality assumption was rejected
## 2. The result of Kruskall_Wallis test:
##  p = 0.096
##  A statistically significant difference do not exist between groups
##
```

Data analysis using R

Boohwi Hong

```
## Present data is ** Fig4_C.action_potential_40_Male.csv **
##
## **  Data structure  **
## 'data.frame':    13 obs. of  4 variables:
##  $  ubject      : int  1 2 3 4 5 6 7 8 9 10 ...
##  $ group           : chr  "control" "control" "control" "control" ...
##  $ input.current   : int  40 40 40 40 40 40 40 40 40 40 ...
##  $ action.potential: num  3.5 3.5 1 8.5 5.33 ...
##
## ** Explorative data analysis with graphics**
```

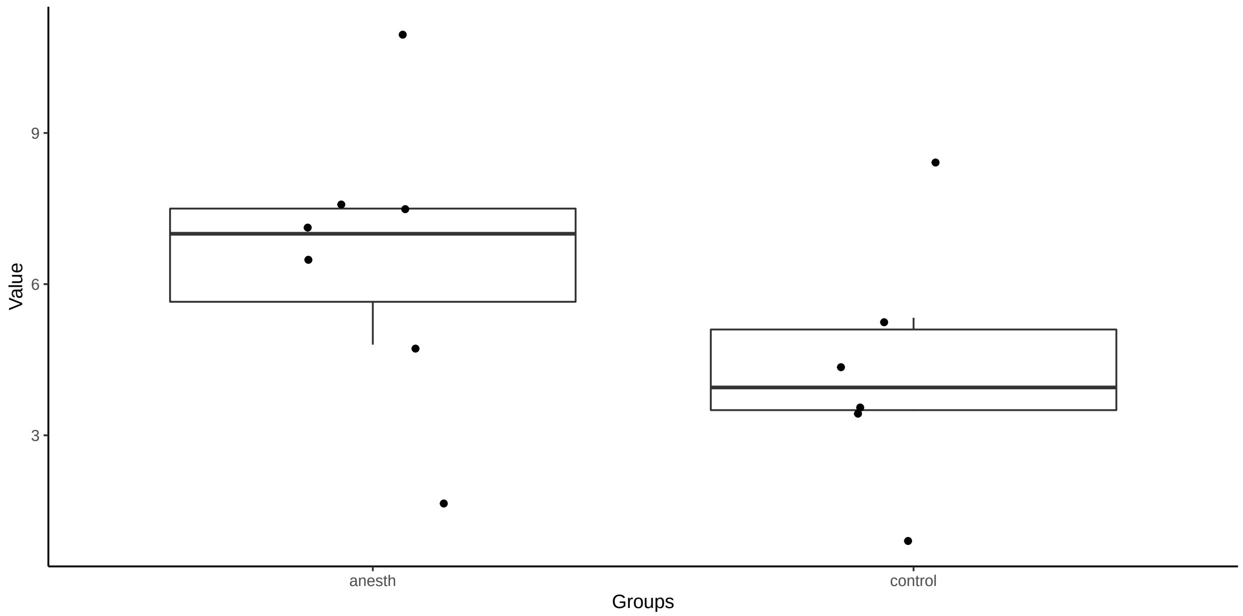


```
## 1. Normality assumption test by Shapiro_Wilk test is
##  p = 0.574
##  Normality assumption was not rejected
## 2. Equal variance test by Bartlett test is
##  p = 0.733
##  Equal variance assumption was not rejected
## 3. The result of anova is
##  p = 0.179
##  A statistically significant difference do not exist between groups
##
```

Data analysis using R

Boohwi Hong

```
## Present data is ** Fig4_C.action_potential_50_Male.csv **
##
## **  Data structure  **
## 'data.frame':    13 obs. of  4 variables:
##  $  ubject      : int  1 2 3 4 5 6 7 8 9 10 ...
##  $ group           : chr  "control" "control" "control" "control" ...
##  $ input.current   : int  50 50 50 50 50 50 50 50 50 50 ...
##  $ action.potential: num  6.5 6.5 4.5 10 8.33 ...
##
## ** Explorative data analysis with graphics**
```

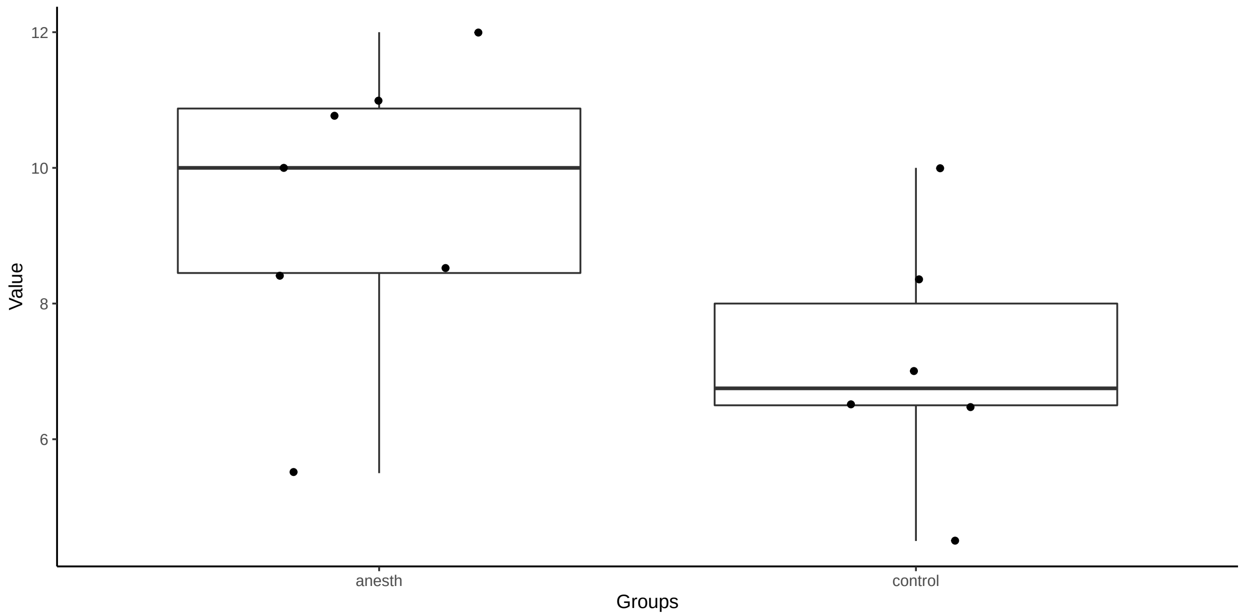


```
## 1. Normality assumption test by Shapiro_Wilk test is
##  p = 0.811
##  Normality assumption was not rejected
## 2. Equal variance test by Bartlett test is
##  p = 0.730
##  Equal variance assumption was not rejected
## 3. The result of anova is
##  p = 0.067
##  A statistically significant difference do not exist between groups
##
```

Data analysis using R

Boohwi Hong

```
## Present data is ** Fig4_C.action_potential_60_Male.csv **
##
## **  Data structure  **
## 'data.frame':    13 obs. of  4 variables:
##  $  ubject      : int  1 2 3 4 5 6 7 8 9 10 ...
##  $ group           : chr  "control" "control" "control" "control" ...
##  $ input.current   : int  60 60 60 60 60 60 60 60 60 60 ...
##  $ action.potential: num  8 8 7 11.5 10 9.2 9.5 12.5 8.5 10.6 ...
##
## ** Explorative data analysis with graphics**
```

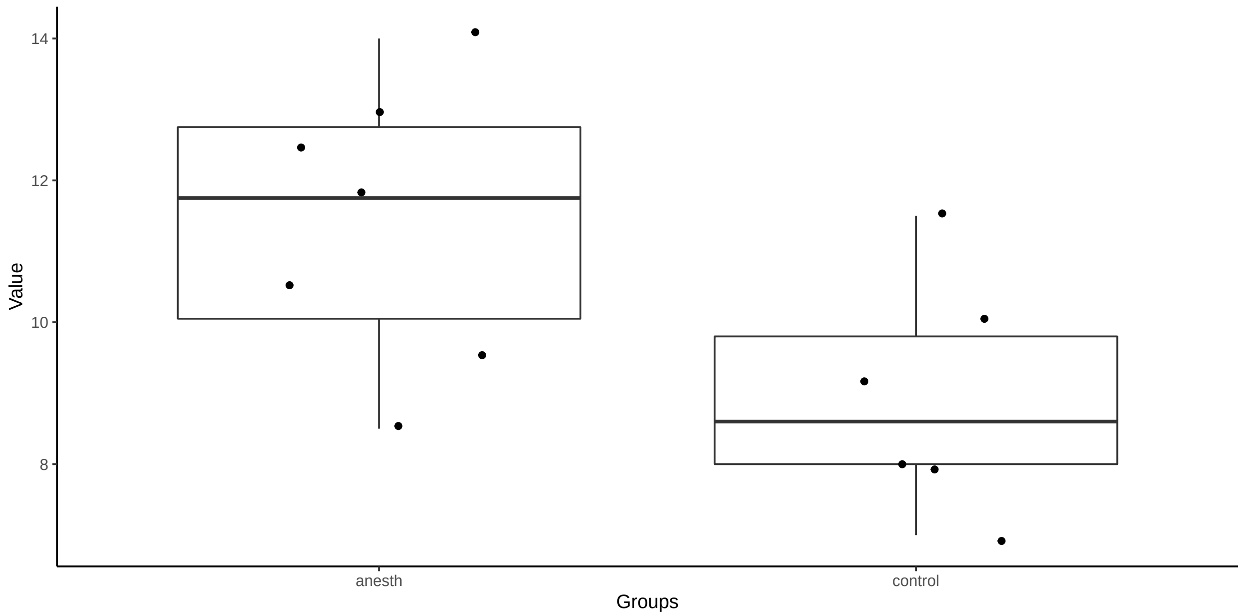


```
## 1. Normality assumption test by Shapiro_Wilk test is
##  p = 0.712
##  Normality assumption was not rejected
## 2. Equal variance test by Bartlett test is
##  p = 0.673
##  Equal variance assumption was not rejected
## 3. The result of anova is
##  p = 0.034
##  A statistically significant difference exist between groups
##
##   Tukey multiple comparisons of means
##     95% family-wise confidence level
```

```
##
## Fit: aov(formula = d1[, 4] ~ d1[, 2], data = d1)
##
## $`d1[, 2]`
##                     diff       lwr
## control-anesth -2.457143 -4.690366
##                       upr     p adj
## control-anesth -0.2239201 0.0339063
```

Data analysis using R

Boohwi Hong

```
## Present data is ** Fig4_C.action_potential_70_Male.csv **
##
## **  Data structure  **
## 'data.frame':    13 obs. of  4 variables:
##  $  ubject      : int  1 2 3 4 5 6 7 8 9 10 ...
##  $ group           : chr  "control" "control" "control" "control" ...
##  $ input.current   : int  70 70 70 70 70 70 70 70 70 70 ...
##  $ action.potential: num  10.5 10.5 9 11.5 11.7 ...
##
## ** Explorative data analysis with graphics**
```

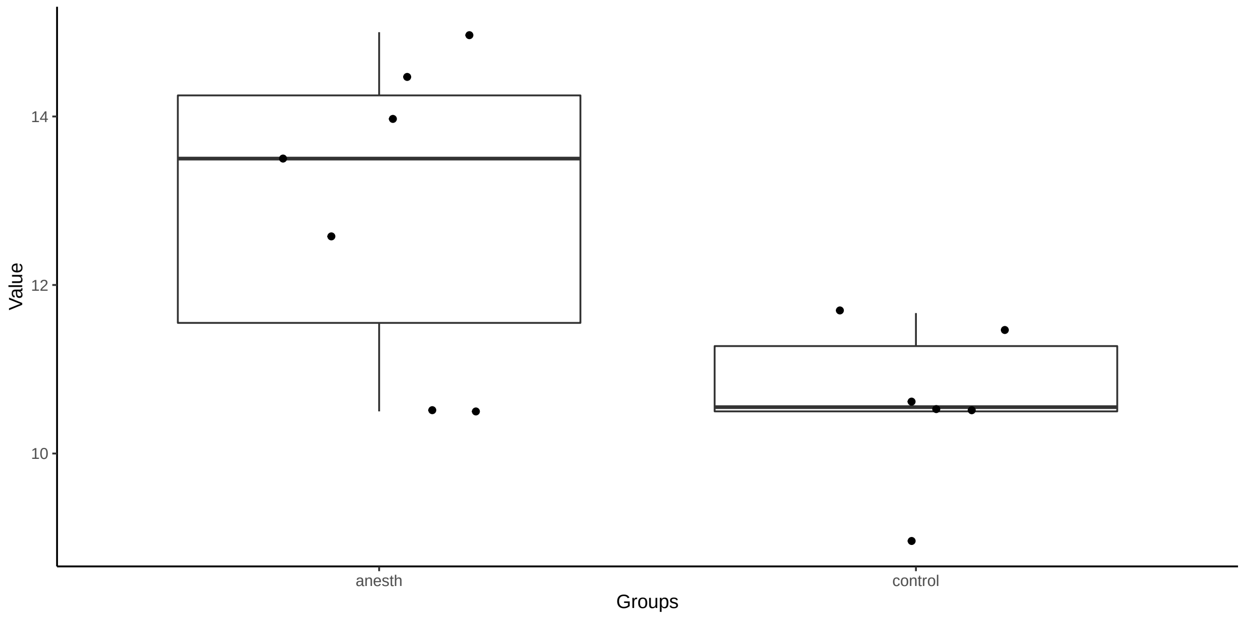


```
## 1. Normality assumption test by Shapiro_Wilk test is
##  p = 0.278
##  Normality assumption was not rejected
## 2. Equal variance test by Bartlett test is
##  p = 0.164
##  Equal variance assumption was not rejected
## 3. The result of anova is
##  p = 0.018
##  A statistically significant difference exist between groups
##
##   Tukey multiple comparisons of means
##     95% family-wise confidence level
```

```
##
## Fit: aov(formula = d1[, 4] ~ d1[, 2], data = d1)
##
## $`d1[, 2]`
##                     diff      lwr
## control-anesth -2.315079 -4.14852
##                       upr     p adj
## control-anesth -0.4816377 0.0179303
```

Data analysis using R

Boohwi Hong

```
## Present data is ** Fig4_C.action_potential_80_Male.csv **
##
## **  Data structure  **
## 'data.frame':    13 obs. of  4 variables:
##  $  ubject      : int  1 2 3 4 5 6 7 8 9 10 ...
##  $ group           : chr  "control" "control" "control" "control" ...
##  $ input.current   : int  80 80 80 80 80 80 80 80 80 80 ...
##  $ action.potential: num  11.5 11.5 10.5 11.5 12.7 ...
##
## ** Explorative data analysis with graphics**
```

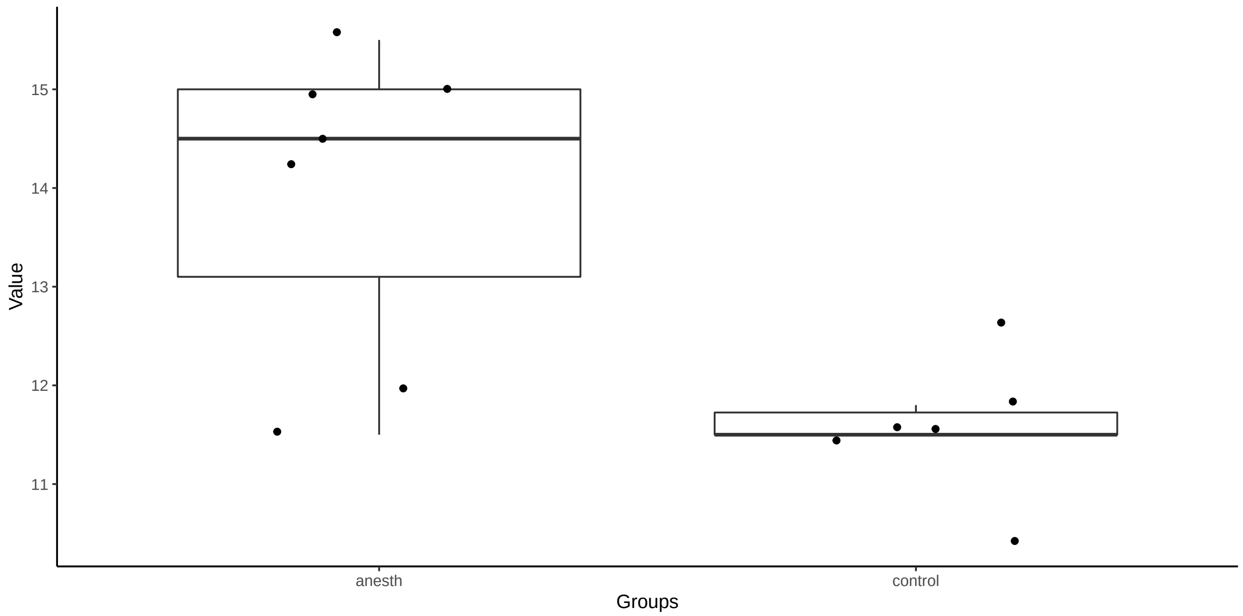


```
## 1. Normality assumption test by Shapiro_Wilk test is
##  p = 0.157
##  Normality assumption was not rejected
## 2. Equal variance test by Bartlett test is
##  p = 0.090
##  Equal variance assumption was not rejected
## 3. The result of anova is
##  p = 0.006
##  A statistically significant difference exist between groups
##
##   Tukey multiple comparisons of means
##     95% family-wise confidence level
```

```
##
## Fit: aov(formula = d1[, 4] ~ d1[, 2], data = d1)
##
## $`d1[, 2]`
##                     diff       lwr
## control-anesth -2.379365 -3.910207
##                       upr     p adj
## control-anesth -0.8485219 0.0057136
```

Data analysis using R

Boohwi Hong

```
## Present data is ** Fig4_C.action_potential_90_Male.csv **
##
## **  Data structure  **
## 'data.frame':    13 obs. of  4 variables:
##  $  ubject      : int  1 2 3 4 5 6 7 8 9 10 ...
##  $ group           : chr  "control" "control" "control" "control" ...
##  $ input.current   : int  90 90 90 90 90 90 90 90 90 90 ...
##  $ action.potential: num  12.5 12.5 11.5 10.5 13.7 ...
##
## ** Explorative data analysis with graphics**
```

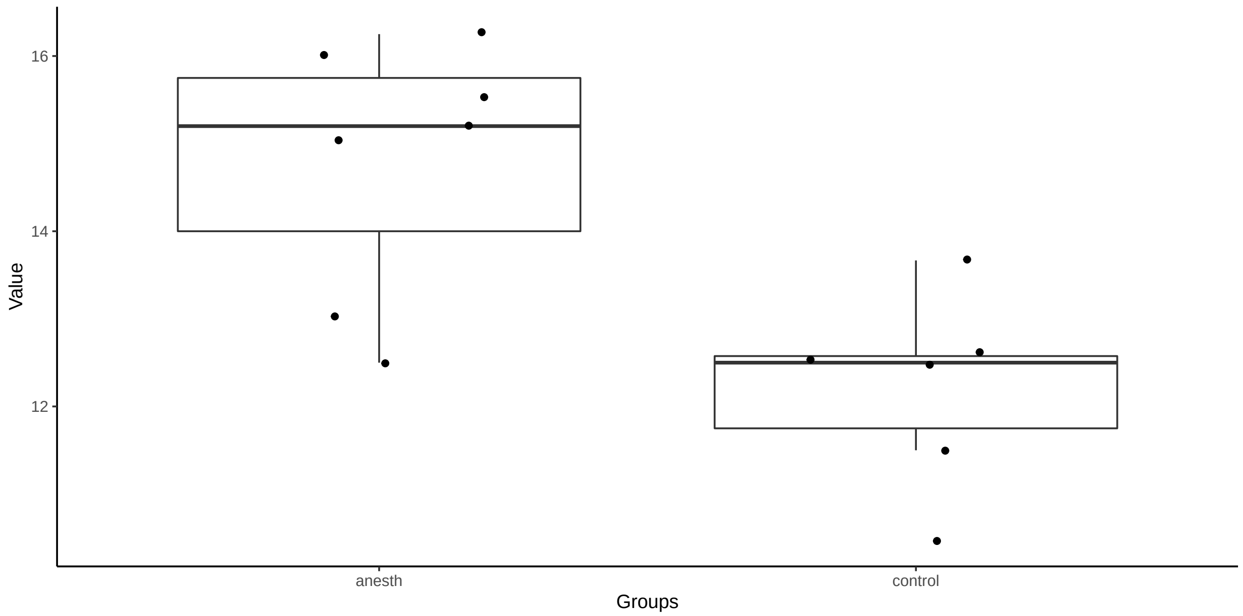


```
## 1. Normality assumption test by Shapiro_Wilk test is
##  p = 0.087
##  Normality assumption was not rejected
## 2. Equal variance test by Bartlett test is
##  p = 0.514
##  Equal variance assumption was not rejected
## 3. The result of anova is
##  p = 0.005
##  A statistically significant difference exist between groups
##
##   Tukey multiple comparisons of means
##     95% family-wise confidence level
```

```
##
## Fit: aov(formula = d1[, 4] ~ d1[, 2], data = d1)
##
## $`d1[, 2]`
##                    diff       lwr
## control-anesth -2.56746 -4.161027
##                       upr     p adj
## control-anesth -0.9738927 0.0045834
```

Data analysis using R

Boohwi Hong

```
## Present data is ** Fig4_C.action_potential_100_Male.csv **
##
## **  Data structure  **
## 'data.frame':    13 obs. of  4 variables:
##  $  ubject      : int  1 2 3 4 5 6 7 8 9 10 ...
##  $ group           : chr  "control" "control" "control" "control" ...
##  $ input.current   : int  100 100 100 100 100 100 100 100 100 100 ...
##  $ action.potential: num  13 13 13 11 14 13.4 12.5 17 14 16.2 ...
##
## ** Explorative data analysis with graphics**
```

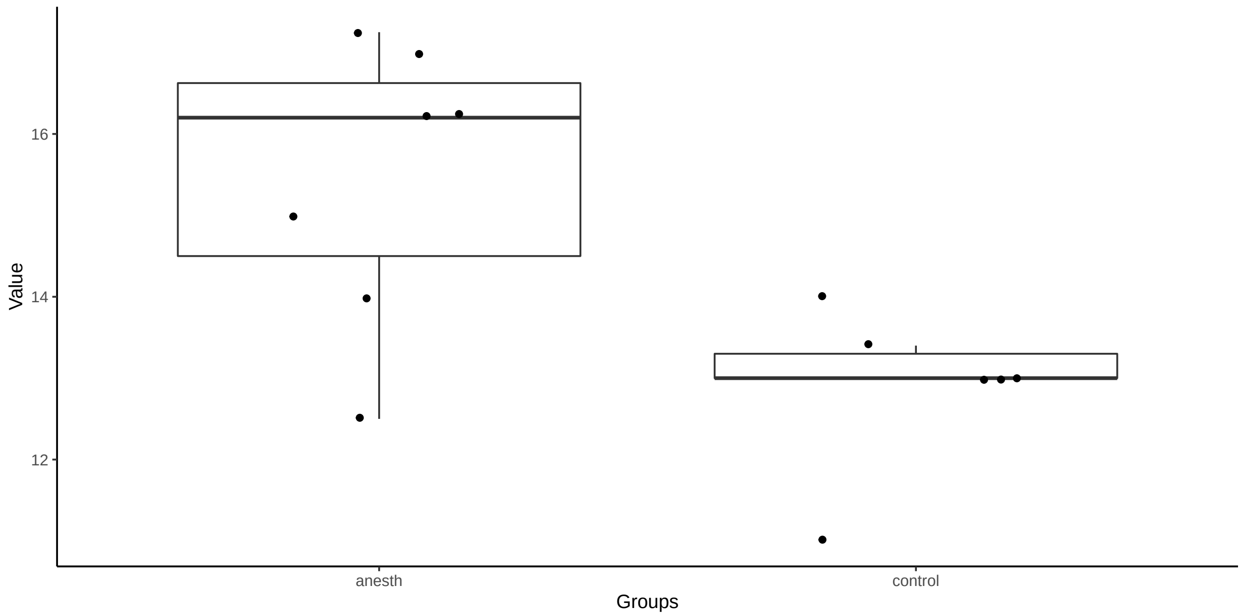


```
## 1. Normality assumption test by Shapiro_Wilk test is
##  p = 0.31
##  Normality assumption was not rejected
## 2. Equal variance test by Bartlett test is
##  p = 0.249
##  Equal variance assumption was not rejected
## 3. The result of anova is
##  p = 0.009
##  A statistically significant difference exist between groups
##
##   Tukey multiple comparisons of means
##     95% family-wise confidence level
```

```
##
## Fit: aov(formula = d1[, 4] ~ d1[, 2], data = d1)
##
## $`d1[, 2]`
##                     diff       lwr
## control-anesth -2.557143 -4.325946
##                     upr     p adj
## control-anesth -0.78834 0.0087309
```

Data analysis using R

Boohwi Hong

```
## Present data is ** Fig4_C.action_potential_110_Male.csv **
##
## **  Data structure  **
## 'data.frame':    13 obs. of  4 variables:
##  $  ubject      : int  1 2 3 4 5 6 7 8 9 10 ...
##  $ group           : chr  "control" "control" "control" "control" ...
##  $ input.current   : int  110 110 110 110 110 110 110 110 110 110 ...
##  $ action.potential: num  13.5 13.5 13.5 10 14 14 13.5 17 14 17.4 ...
##
## ** Explorative data analysis with graphics**
```

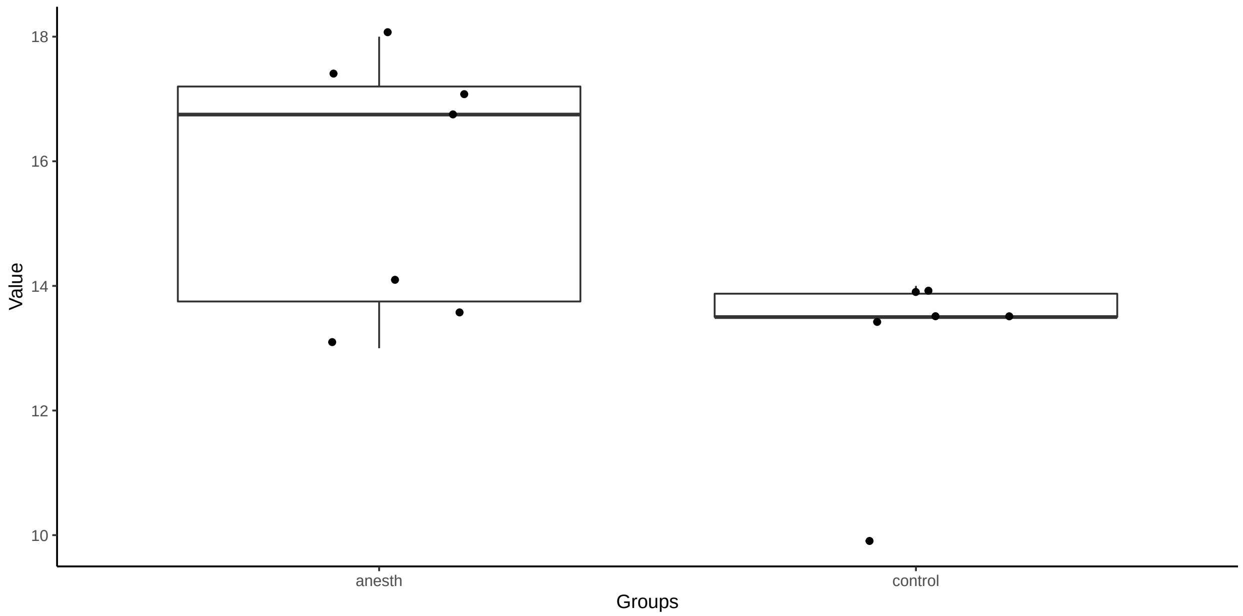


```
## 1. Normality assumption test by Shapiro_Wilk test is
##  p = 0.073
##  Normality assumption was not rejected
## 2. Equal variance test by Bartlett test is
##  p = 0.499
##  Equal variance assumption was not rejected
## 3. The result of anova is
##  p = 0.029
##  A statistically significant difference exist between groups
##
##   Tukey multiple comparisons of means
##     95% family-wise confidence level
```

```
##
## Fit: aov(formula = d1[, 4] ~ d1[, 2], data = d1)
##
## $`d1[, 2]`
##                     diff       lwr
## control-anesth -2.580952 -4.847641
##                       upr     p adj
## control-anesth -0.3142639 0.0291861
```

Data analysis using R

Boohwi Hong

```
## Present data is ** Fig4_D.sEPSC_amplitude_Male.csv **
##
## **  Data structure  **
## 'data.frame':    15 obs. of  3 variables:
##  $  ubject: int  1 2 3 4 5 6 7 8 9 10 ...
##  $ group     : chr  "control" "control" "control" "control" ...
##  $ amplitude : num  22.7 22.8 17.5 13.4 25.4 ...
##
## ** Explorative data analysis with graphics**
```

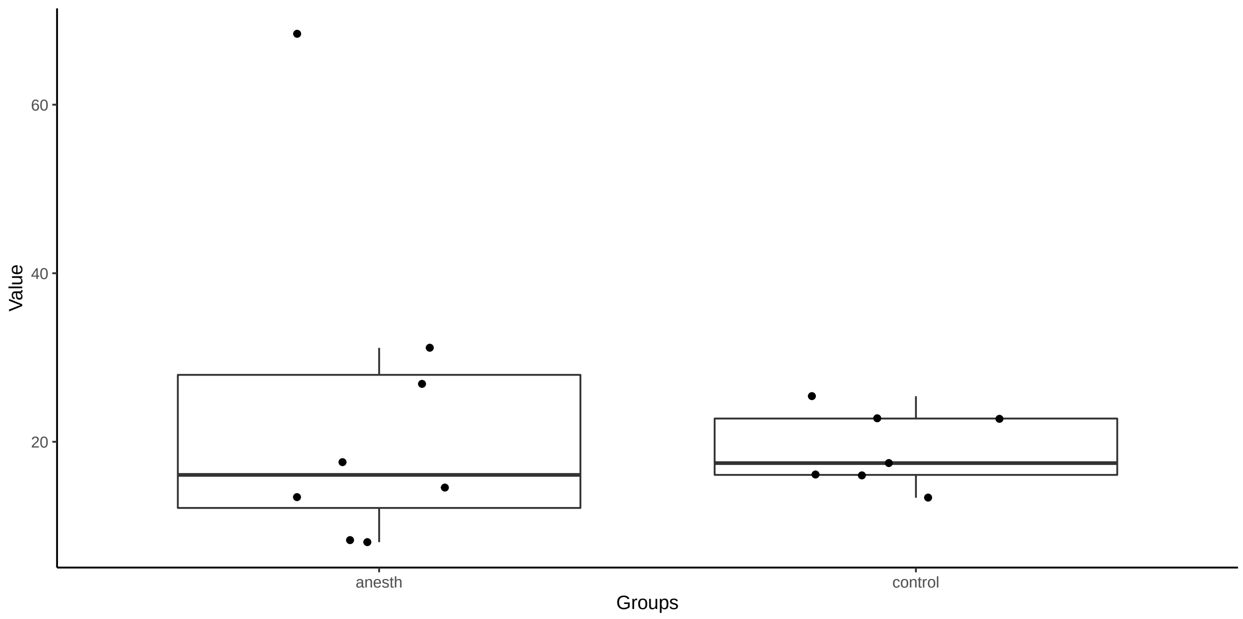


```
## 1. Normality assumption test by Shapiro_Wilk test is
##  p = 0.002
##  Normality assumption was rejected
## 2. The result of Kruskall_Wallis test:
##  p = 0.908
##  A statistically significant difference do not exist between groups
##
```

Data analysis using R

Boohwi Hong

```
## Present data is ** Fig4_D.sEPSC_freq_Male.csv **
##
## **  Data structure  **
## 'data.frame':    15 obs. of  3 variables:
##  $  ubject: int  1 2 3 4 5 6 7 8 9 10 ...
##  $ group     : chr  "control" "control" "control" "control" ...
##  $ freq      : num  0.0333 0.15 0.3833 0.2611 0.1708 ...
##
## ** Explorative data analysis with graphics**
```

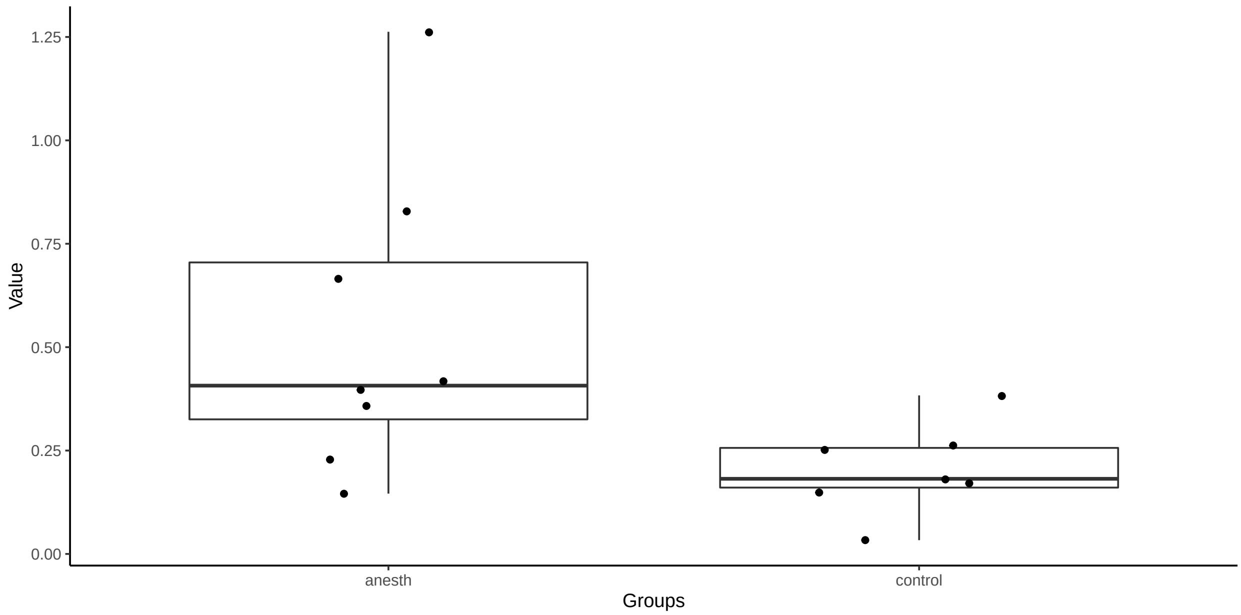


```
## 1. Normality assumption test by Shapiro_Wilk test is
##  p = 0.149
##  Normality assumption was not rejected
## 2. Equal variance test by Bartlett test is
##  p = 0.009
##  Equal variance assumption was rejected
## 3. The result of Welch ANOVA is
##  p = 0.039
##  A statistically significant difference exist between groups
##
##   Tukey multiple comparisons of means
##     95% family-wise confidence level
##
```

```
## Fit: aov(formula = d1[, 3] ~ d1[, 2])
##
## $`d1[, 2]`
##                      diff        lwr
## control-anesth -0.3327977 -0.6450026
##                        upr     p adj
## control-anesth -0.02059275 0.0384539
```

Data analysis using R

Boohwi Hong

```
## Present data is ** Fig4_E.sIPSC_amplitude_Male.csv **
##
## **  Data structure  **
## 'data.frame':    14 obs. of  3 variables:
##  $  ubject: int  1 2 3 4 5 6 7 8 9 10 ...
##  $ group     : chr  "control" "control" "control" "control" ...
##  $ amp       : num  67.1 47.1 54.3 37.2 34.1 ...
##
## ** Explorative data analysis with graphics**
```

```
## Warning: Removed 2 rows containing non-finite
## values (stat_boxplot).
```

```
## Warning: Removed 2 rows containing missing
## values (geom_point).
```

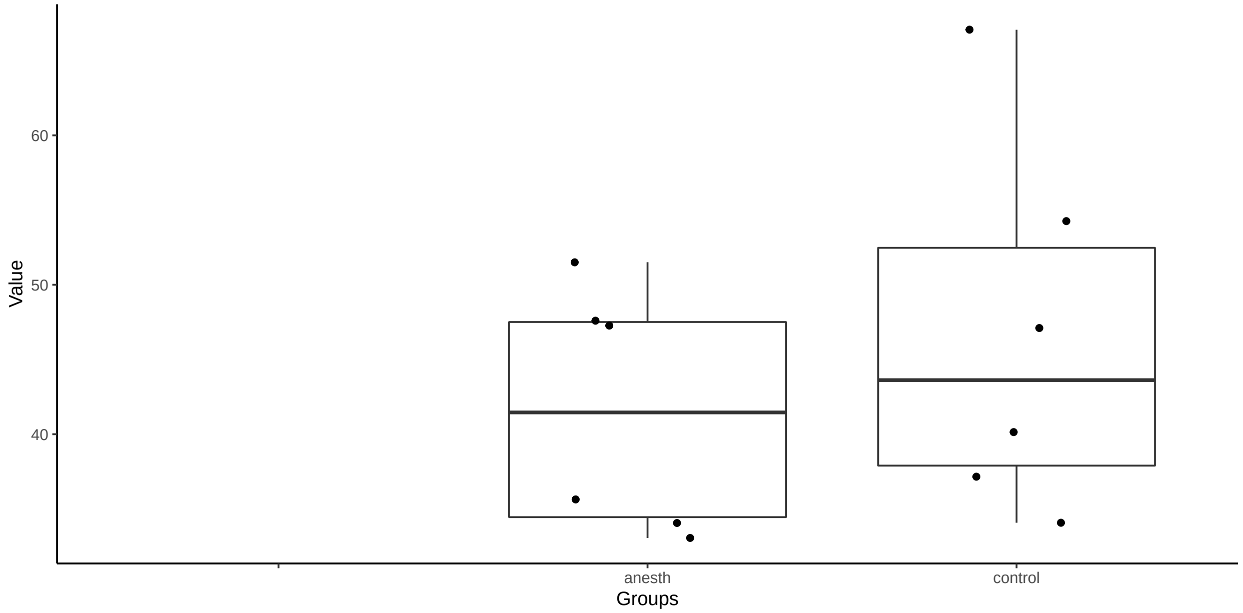


```
## 1. Normality assumption test by Shapiro_Wilk test is
##  p = 0.291
##  Normality assumption was not rejected
## 2. Equal variance test by Bartlett test is
##  p = 0.379
##  Equal variance assumption was not rejected
## 3. The result of anova is
```

```
##  p = 0.418
##  A statistically significant difference do not exist between groups
##
```

Data analysis using R

Boohwi Hong

```
## Present data is ** Fig4_E.sIPSC_freq_Male.csv **
##
## **  Data structure  **
## 'data.frame':    14 obs. of  3 variables:
##  $  ubject: int  1 2 3 4 5 6 7 8 9 10 ...
##  $ group     : chr  "control" "control" "control" "control" ...
##  $ freq      : num  9.45 8.43 11.12 10.45 12.64 ...
##
## ** Explorative data analysis with graphics**
```

```
## Warning: Removed 2 rows containing non-finite
## values (stat_boxplot).
```

```
## Warning: Removed 2 rows containing missing
## values (geom_point).
```

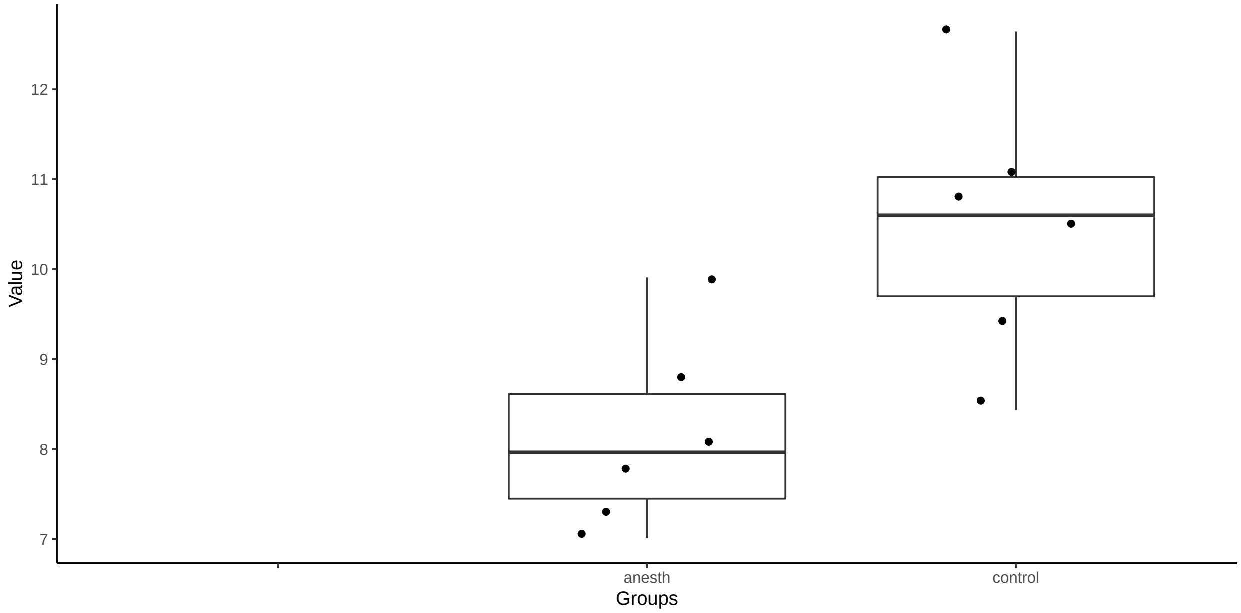


```
## 1. Normality assumption test by Shapiro_Wilk test is
##  p = 0.959
##  Normality assumption was not rejected
## 2. Equal variance test by Bartlett test is
##  p = 0.507
##  Equal variance assumption was not rejected
## 3. The result of anova is
```

```
##  p = 0.010
##  A statistically significant difference exist between groups
##
##   Tukey multiple comparisons of means
##     95% family-wise confidence level
##
## Fit: aov(formula = d1[, 3] ~ d1[, 2], data = d1)
##
## $`d1[, 2]`
##                    diff       lwr
## control-anesth 2.314447 0.6888304
##                     upr     p adj
## control-anesth 3.940064 0.0099491
```

Data analysis using R

Boohwi Hong

```
## Present data is ** Fig4_F.sAP_freq_Male.csv **
##
## **  Data structure  **
## 'data.frame':    14 obs. of  3 variables:
##  $  ubject: int  1 2 3 4 5 6 7 8 9 10 ...
##  $ group     : chr  "control" "control" "control" "control" ...
##  $ freq      : num  0 0.000833 0 0.000667 0.000333 ...
##
## ** Explorative data analysis with graphics**
```

```
## Warning: Removed 2 rows containing non-finite
## values (stat_boxplot).
```

```
## Warning: Removed 2 rows containing missing
## values (geom_point).
```

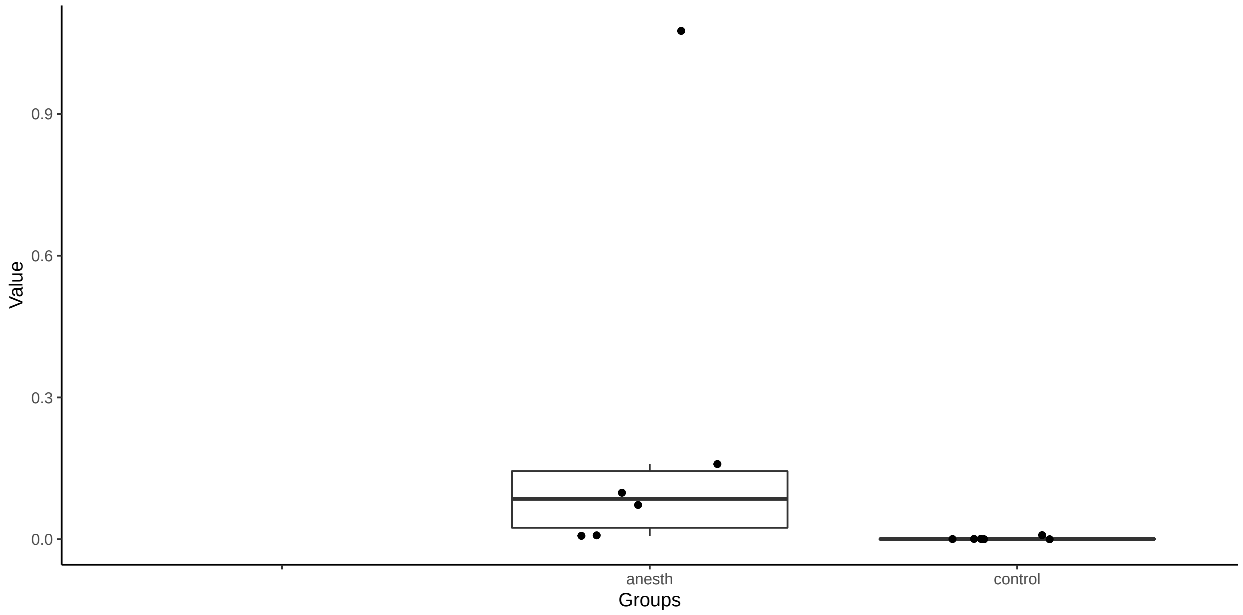


```
## 1. Normality assumption test by Shapiro_Wilk test is
##  p = 0.000
##  Normality assumption was rejected
## 2. The result of Kruskall_Wallis test:
##  p = 0.010
##  A statistically significant difference exist between groups
##
```

Supplementary Note 5

The results of statistical analysis for Figure 5

Fig5_A.kata-CPP_Anesthesia_Female Data analysis using R

Boohwi Hong

Statistical Result

```
## Present data is ** Fig5_A.kata-CPP_Anesthesia_Female.csv ** ##
## **  Data structure  **
## 'data.frame':    54 obs. of  3 variables:
##  $ subject : int  1 2 3 4 5 6 7 8 9 10 ...
##  $ group   : chr  "pre" "pre" "pre" "pre" ...
##  $ duration: num  370 249 279 236 259 ...
##
## ** Explorative data analysis with graphics**
```

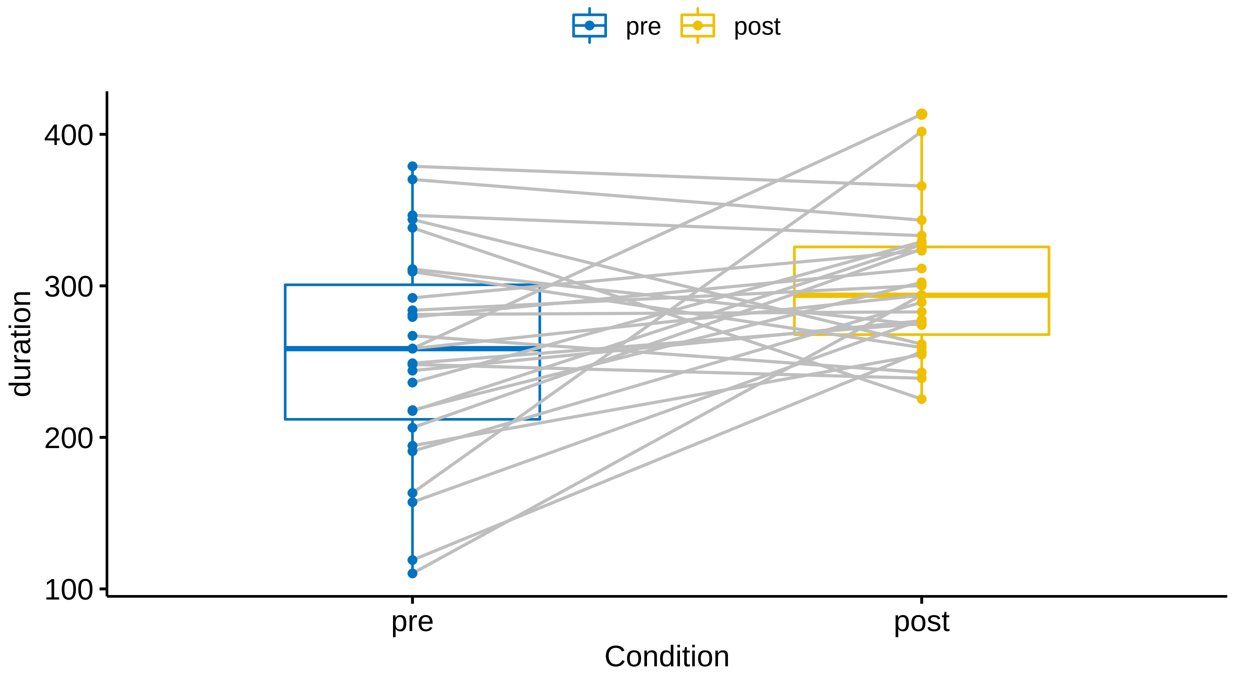


```
## 1. Normality assumption test by Shapiro_Wilk test is
##  p = 0.647
##  Normality assumption was not rejected
## 2. The result of paired t-test is
##  p = 0.010
##  A statistically significant difference exist between groups
```

Fig5_A.kata-CPP_Control_Female Data analysis using R

Boohwi Hong

Statistical Result

```
## Present data is ** Fig5_A.kata-CPP_Control_Female.csv ** ##
## **  Data structure  **
## 'data.frame':    50 obs. of  3 variables:
##$  ubject: int 1 2 3 4 5 6 7 8 9 10 ...
##  $ group     : chr  "pre" "pre" "pre" "pre" ...
##  $ duration  : num  215 334 133 200 232 ...
##
## ** Explorative data analysis with graphics**
```

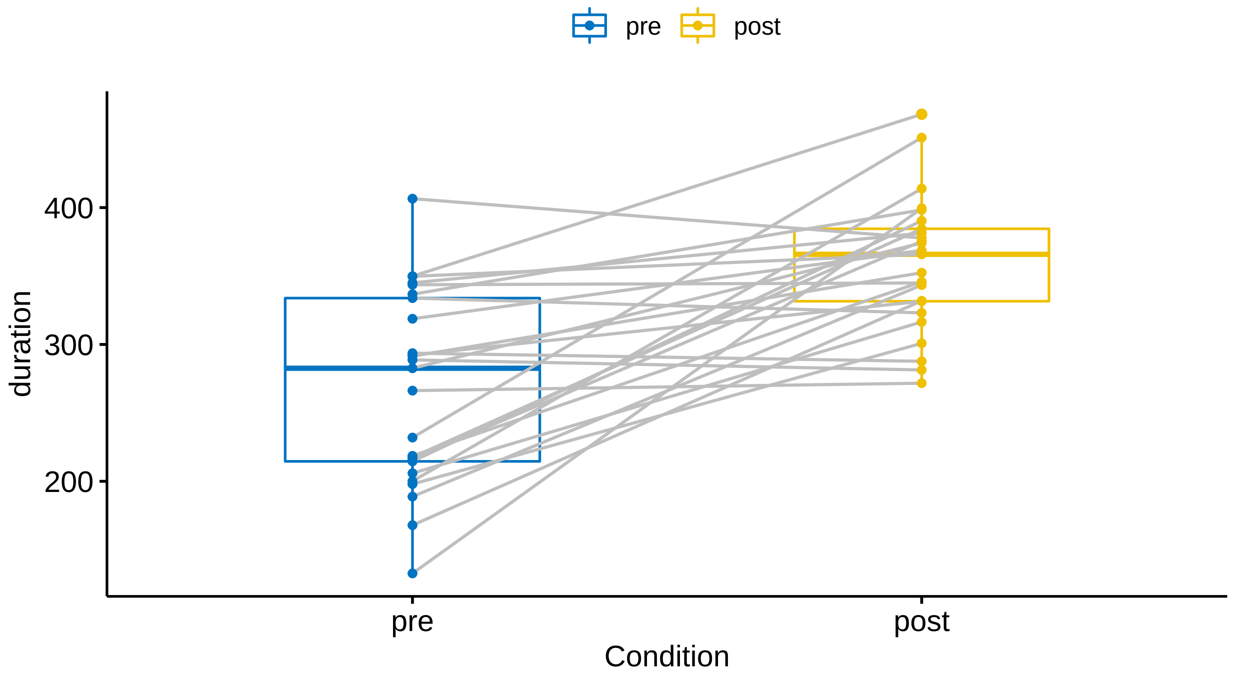


```
## 1. Normality assumption test by Shapiro_Wilk test is
##  p = 0.882
##  Normality assumption was not rejected
## 2. The result of paired t-test is
##  p = 0.000
##  A statistically significant difference exist between groups
```

Fig5_B.keta-CPP_CPPscore_Female Data analysis using R

Boohwi Hong

```
## Present data is ** Fig5_B.keta-CPP_CPPscore_Female.csv ** ##
## **  Data structure  **

## 'data.frame':    52 obs. of  3 variables:
##  $  ubject: int  1 2 3 4 5 6 7 8 9 10 ...
##  $ group     : chr  "control" "control" "control" "control" ...
##  $ difference: num  175.8 -10.8 266.8 213.7 219.2 ...
##
## ** Explorative data analysis with graphics**
```

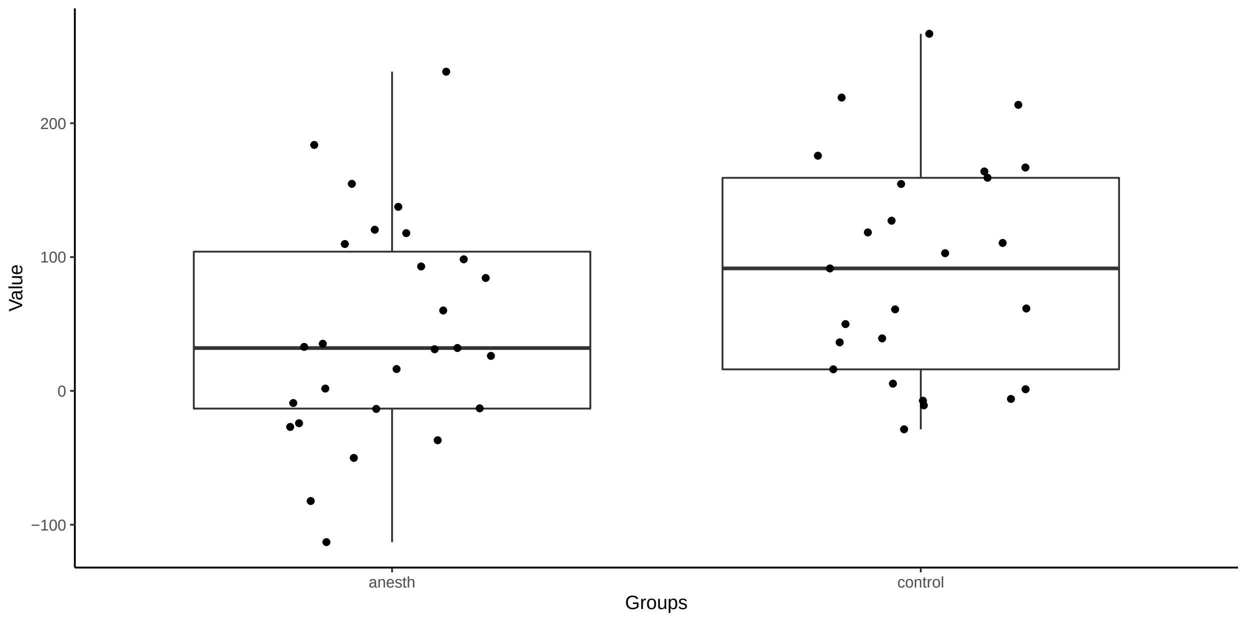


```
## 1. Normality assumption test by Shapiro_Wilk test is
##  p = 0.398
##  Normality assumption was not rejected
## 2. Equal variance test by Bartlett test is
##  p = 1.000
##  Equal variance assumption was not rejected
## 3. The result of anova is
##  p = 0.047
##  A statistically significant difference exist between groups
##
##   Tukey multiple comparisons of means
##     95% family-wise confidence level
##
```

```
## Fit: aov(formula = d1[, 3] ~ d1[, 2], data = d1)
##
## $`d1[, 2]`
##                   diff       lwr      upr     p adj
## control-anesth 46.9187 0.5823843 93.25502 0.0472947
```

Data analysis using R

Boohwi Hong

```
## Present data is ** Fig5_C.action_potential_20_Female.csv **
##
## **  Data structure  **
## 'data.frame':    12 obs. of  4 variables:
##  $  ubject      : int  1 2 3 4 5 6 7 8 9 10 ...
##  $ group           : chr  "control" "control" "control" "control" ...
##  $ input.current   : int  20 20 20 20 20 20 20 20 20 20 ...
##  $ action.potential: num  0 0 0 0 2.75 0 0 0 0 0 ...
##
## ** Explorative data analysis with graphics**
```

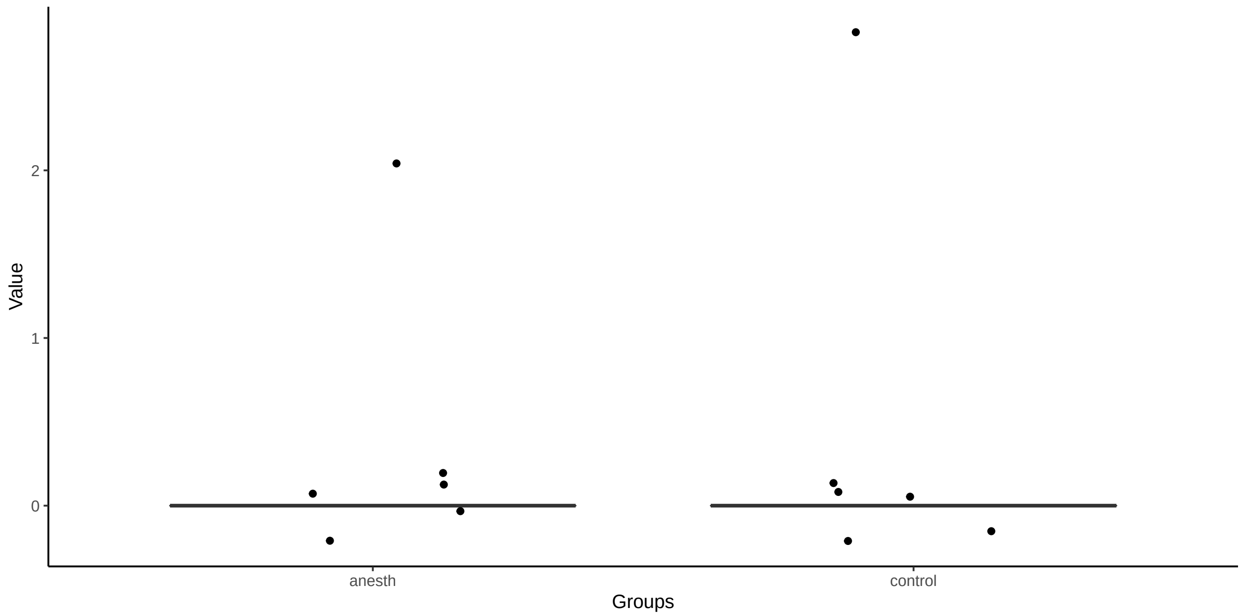


```
## 1. Normality assumption test by Shapiro_Wilk test is
##  p = 0.000
##  Normality assumption was rejected
## 2. The result of Kruskall_Wallis test:
##  p = 0.902
##  A statistically significant difference do not exist between groups
##
```

Data analysis using R

Boohwi Hong

```
## Present data is ** Fig5_C.action_potential_30_Female.csv **
##
## **  Data structure  **
## 'data.frame':    12 obs. of  4 variables:
##  $  ubject      : int  1 2 3 4 5 6 7 8 9 10 ...
##  $ group           : chr  "control" "control" "control" "control" ...
##  $ input.current   : int  30 30 30 30 30 30 30 30 30 30 ...
##  $ action.potential: num  0 0 0 2.2 7.5 2.6 0 1 0 2.4 ...
##
## ** Explorative data analysis with graphics**
```

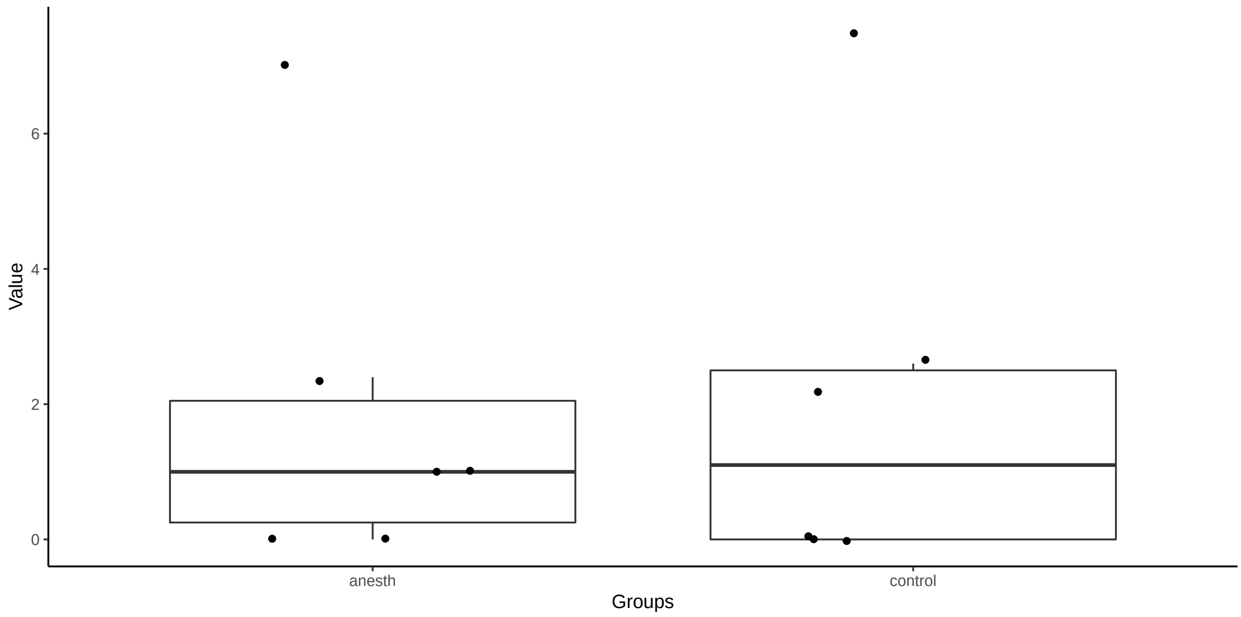


```
## 1. Normality assumption test by Shapiro_Wilk test is
##  p = 0.003
##  Normality assumption was rejected
## 2. The result of Kruskall_Wallis test:
##  p = 1.000
##  A statistically significant difference do not exist between groups
##
```

Data analysis using R

Boohwi Hong

```
## Present data is ** Fig5_C.action_potential_40_Female.csv **
##
## **  Data structure  **
## 'data.frame':    12 obs. of  4 variables:
##  $  ubject      : int  1 2 3 4 5 6 7 8 9 10 ...
##  $ group           : chr  "control" "control" "control" "control" ...
##  $ input.current   : int  40 40 40 40 40 40 40 40 40 40 ...
##  $ action.potential: num  4 3 5.5 5 10.5 6.4 4 4 1 5.8 ...
##
## ** Explorative data analysis with graphics**
```

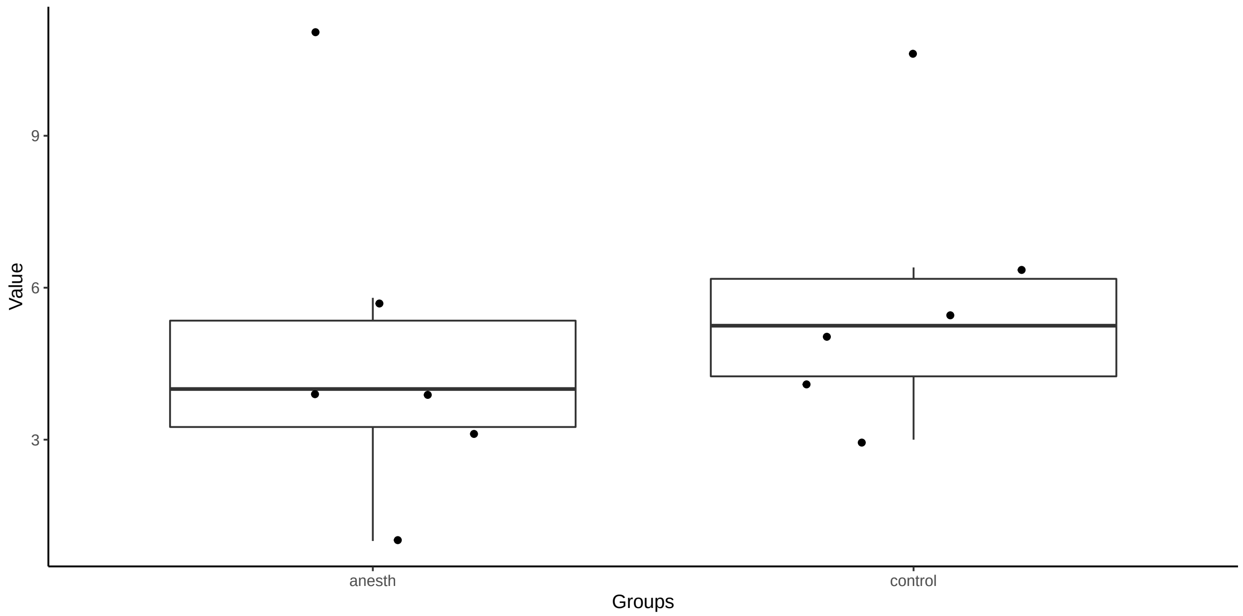


```
## 1. Normality assumption test by Shapiro_Wilk test is
##  p = 0.106
##  Normality assumption was not rejected
## 2. Equal variance test by Bartlett test is
##  p = 0.571
##  Equal variance assumption was not rejected
## 3. The result of anova is
##  p = 0.607
##  A statistically significant difference do not exist between groups
##
```

Data analysis using R

Boohwi Hong

```
## Present data is ** Fig5_C.action_potential_50_Female.csv **
##
## **  Data structure  **
## 'data.frame':    12 obs. of  16 variables:
##  $  ubject      : int  1 2 3 4 5 6 7 8 9 10 ...
##  $ group           : chr  "control" "control" "control" "control" ...
##  $ input.current   : int  50 50 50 50 50 50 50 50 50 50 ...
##  $ action.potential: num  8 7 9.5 9.4 13 10.2 7.5 8 5.5 8 ...
##  $ X               : logi  NA NA NA NA NA NA ...
##  $ X.1             : logi  NA NA NA NA NA NA ...
##  $ X.2             : logi  NA NA NA NA NA NA ...
##  $ X.3             : logi  NA NA NA NA NA NA ...
##  $ X.4             : logi  NA NA NA NA NA NA ...
##  $ X.5             : logi  NA NA NA NA NA NA ...
##  $ X.6             : logi  NA NA NA NA NA NA ...
##  $ X.7             : logi  NA NA NA NA NA NA ...
##  $ X.8             : logi  NA NA NA NA NA NA ...
##  $ X.9             : logi  NA NA NA NA NA NA ...
##  $ X.10            : logi  NA NA NA NA NA NA ...
##  $ X.11            : logi  NA NA NA NA NA NA ...
##
## ** Explorative data analysis with graphics**
```

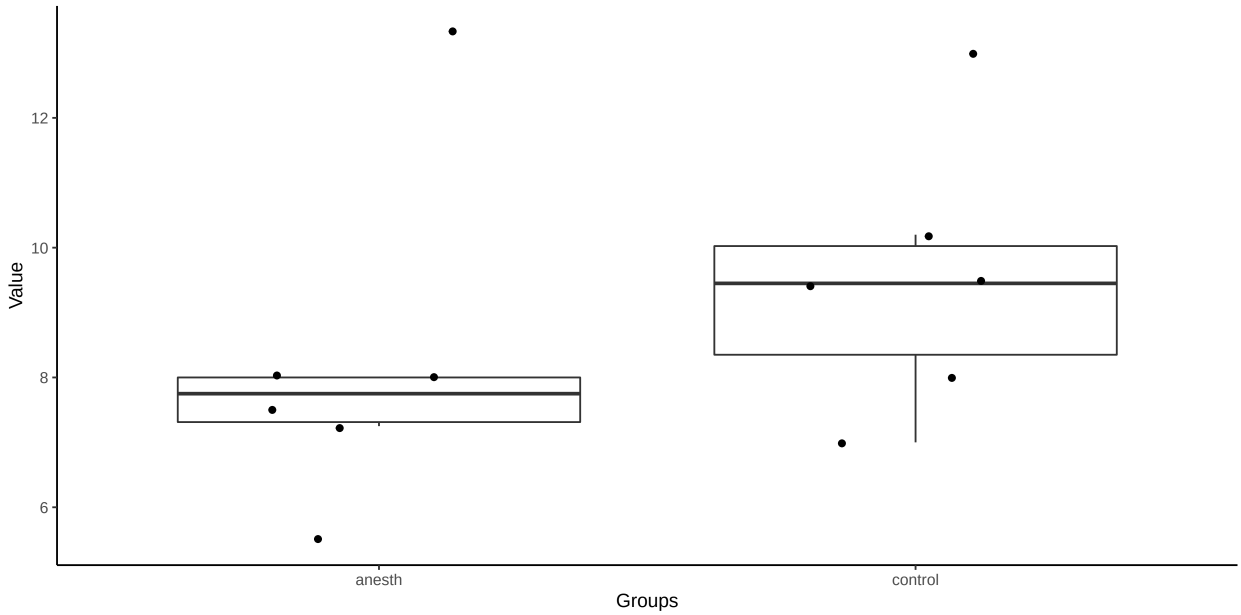


```
## 1. Normality assumption test by Shapiro_Wilk test is
```

```
##  p = 0.074
##  Normality assumption was not rejected
## 2. Equal variance test by Bartlett test is
##  p = 0.595
##  Equal variance assumption was not rejected
## 3. The result of anova is
##  p = 0.382
##  A statistically significant difference do not exist between groups
##
```

Data analysis using R

Boohwi Hong

```
## Present data is ** Fig5_C.action_potential_60_Female.csv **
##
## **  Data structure  **
## 'data.frame':    12 obs. of  4 variables:
##  $  ubject      : int  1 2 3 4 5 6 7 8 9 10 ...
##  $ group           : chr  "control" "control" "control" "control" ...
##  $ input.current   : int  60 60 60 60 60 60 60 60 60 60 ...
##  $ action.potential: num  10.5 10 12 12.4 13.8 ...
##
## ** Explorative data analysis with graphics**
```

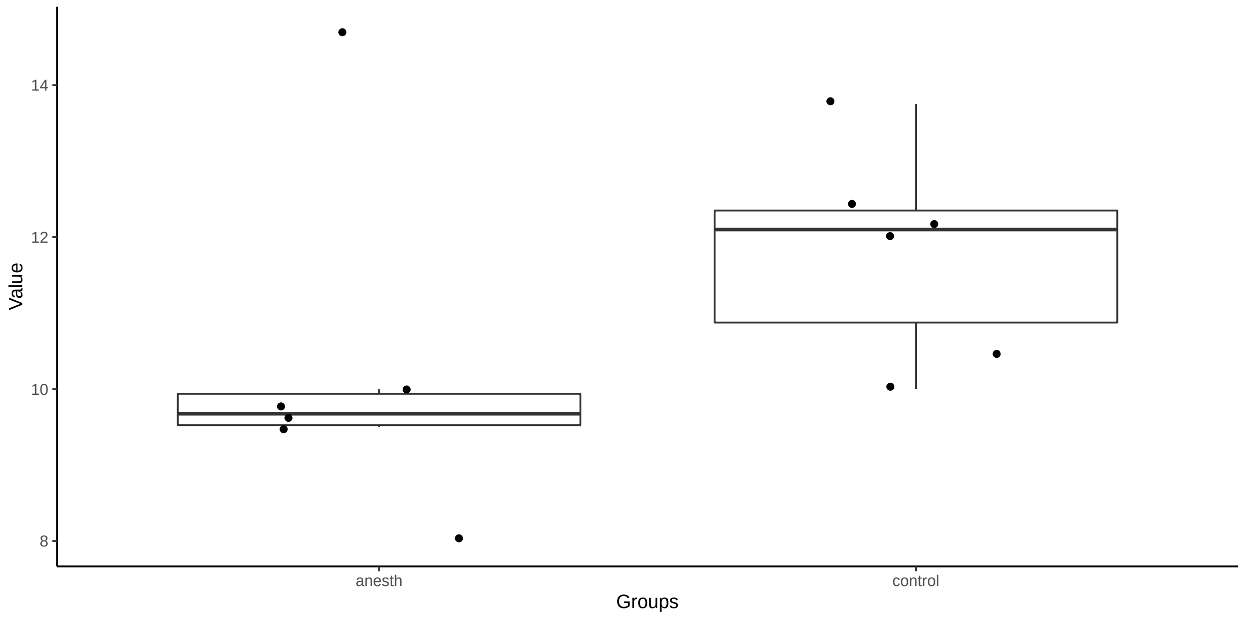


```
## 1. Normality assumption test by Shapiro_Wilk test is
##  p = 0.137
##  Normality assumption was not rejected
## 2. Equal variance test by Bartlett test is
##  p = 0.285
##  Equal variance assumption was not rejected
## 3. The result of anova is
##  p = 0.181
##  A statistically significant difference do not exist between groups
##
```

Data analysis using R

Boohwi Hong

```
## Present data is ** Fig5_C.action_potential_70_Female.csv **
##
## **  Data structure  **
## 'data.frame':    12 obs. of  4 variables:
##  $  ubject      : int  1 2 3 4 5 6 7 8 9 10 ...
##  $ group           : chr  "control" "control" "control" "control" ...
##  $ input.current   : int  70 70 70 70 70 70 70 70 70 70 ...
##  $ action.potential: num  12.5 12 14 14 15 13.6 11 12 9.5 11.4 ...
##
## ** Explorative data analysis with graphics**
```

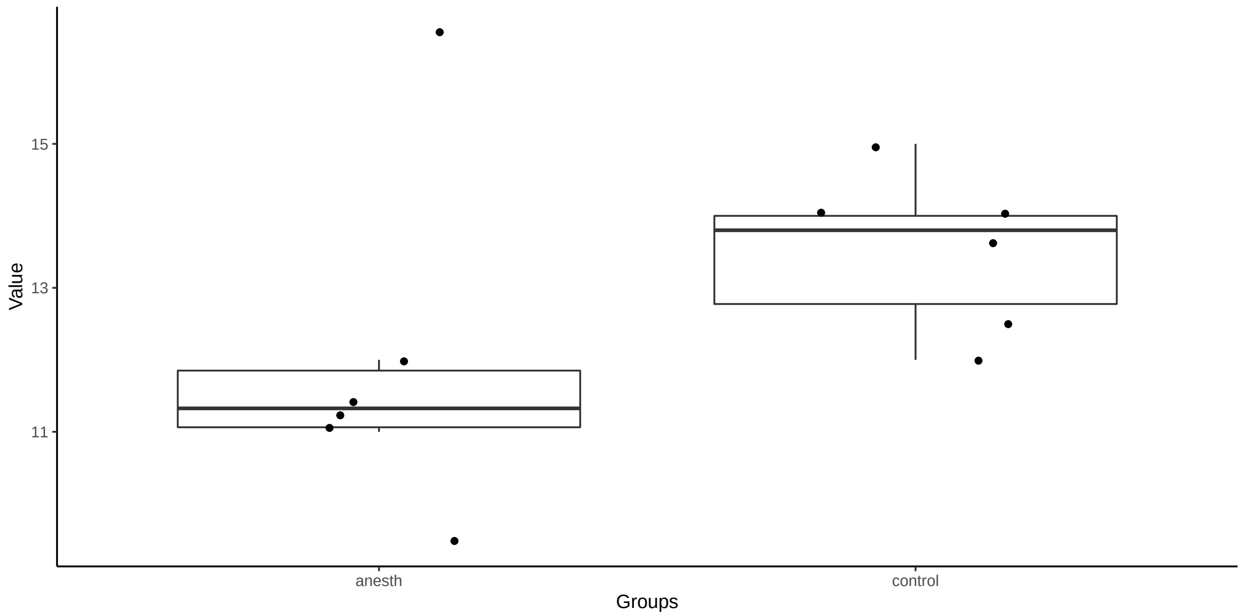


```
## 1. Normality assumption test by Shapiro_Wilk test is
##  p = 0.084
##  Normality assumption was not rejected
## 2. Equal variance test by Bartlett test is
##  p = 0.114
##  Equal variance assumption was not rejected
## 3. The result of anova is
##  p = 0.172
##  A statistically significant difference do not exist between groups
##
```

Data analysis using R

Boohwi Hong

```
## Present data is ** Fig5_C.action_potential_80_Female.csv **
##
## **  Data structure  **
## 'data.frame':    12 obs. of  4 variables:
##  $  ubject      : int  1 2 3 4 5 6 7 8 9 10 ...
##  $ group           : chr  "control" "control" "control" "control" ...
##  $ input.current   : int  80 80 80 80 80 80 80 80 80 80 ...
##  $ action.potential: num  13.5 13.5 15 15.8 15.2 ...
##
## ** Explorative data analysis with graphics**
```

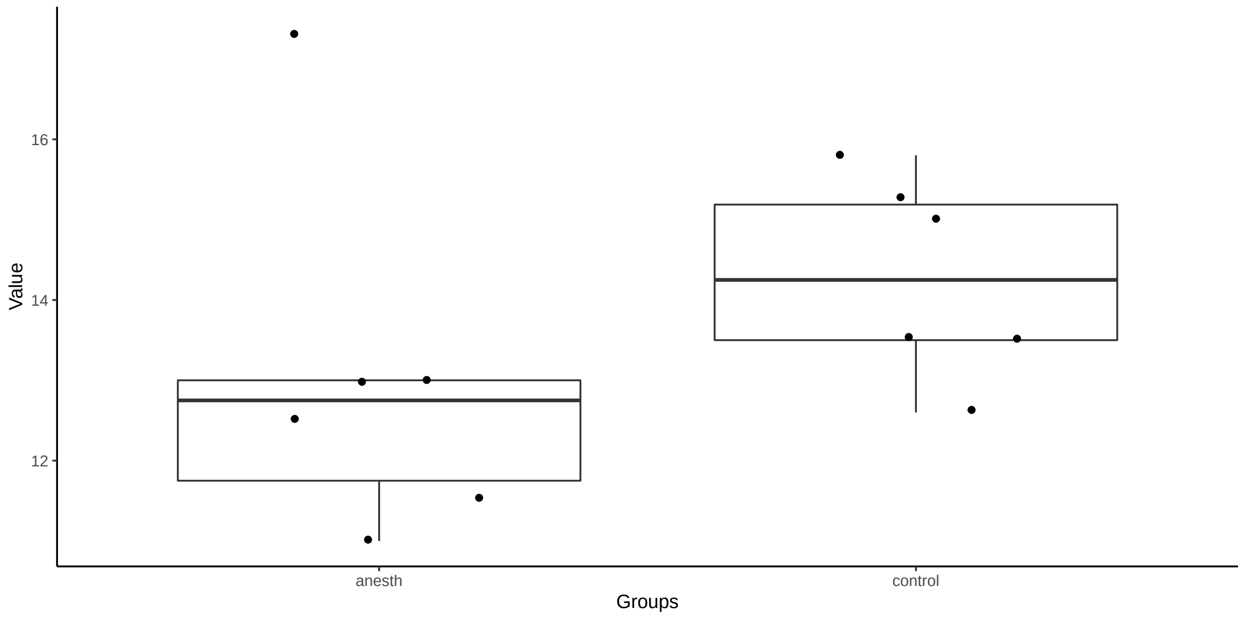


```
## 1. Normality assumption test by Shapiro_Wilk test is
##  p = 0.143
##  Normality assumption was not rejected
## 2. Equal variance test by Bartlett test is
##  p = 0.223
##  Equal variance assumption was not rejected
## 3. The result of anova is
##  p = 0.272
##  A statistically significant difference do not exist between groups
##
```

Data analysis using R

Boohwi Hong

```
## Present data is ** Fig5_C.action_potential_90_Female.csv **
##
## **  Data structure  **
## 'data.frame':    12 obs. of  4 variables:
##  $  ubject      : int  1 2 3 4 5 6 7 8 9 10 ...
##  $ group           : chr  "control" "control" "control" "control" ...
##  $ input.current   : int  90 90 90 90 90 90 90 90 90 90 ...
##  $ action.potential: num  14.5 14.5 16 17.2 16 15.6 12.5 14 11.5 14.2 ...
##
## ** Explorative data analysis with graphics**
```

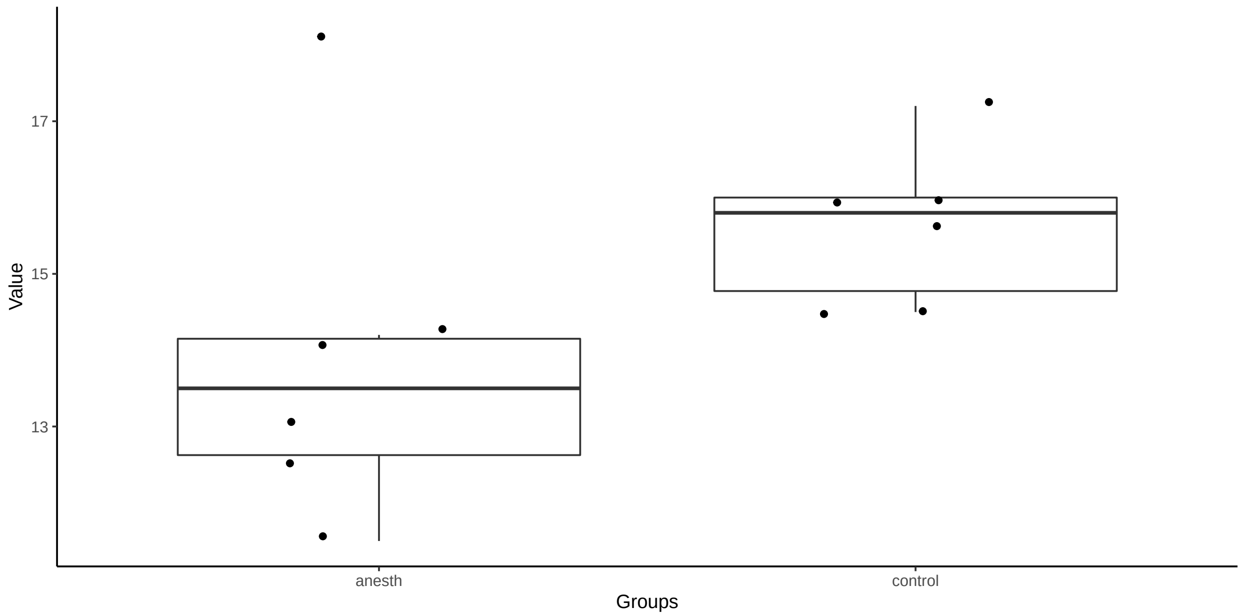


```
## 1. Normality assumption test by Shapiro_Wilk test is
##  p = 0.109
##  Normality assumption was not rejected
## 2. Equal variance test by Bartlett test is
##  p = 0.099
##  Equal variance assumption was not rejected
## 3. The result of anova is
##  p = 0.124
##  A statistically significant difference do not exist between groups
##
```

Data analysis using R

Boohwi Hong

```
## Present data is ** Fig5_C.action_potential_100_Female.csv **
##
## **  Data structure  **
## 'data.frame':    12 obs. of  4 variables:
##  $  ubject      : int  1 2 3 4 5 6 7 8 9 10 ...
##  $ group           : chr  "control" "control" "control" "control" ...
##  $ input.current   : int  100 100 100 100 100 100 100 100 100 100 ...
##  $ action.potential: num  15.5 15.5 17 18.6 16 16.4 13.5 14.5 12.5 15.2 ...
##
## ** Explorative data analysis with graphics**
```

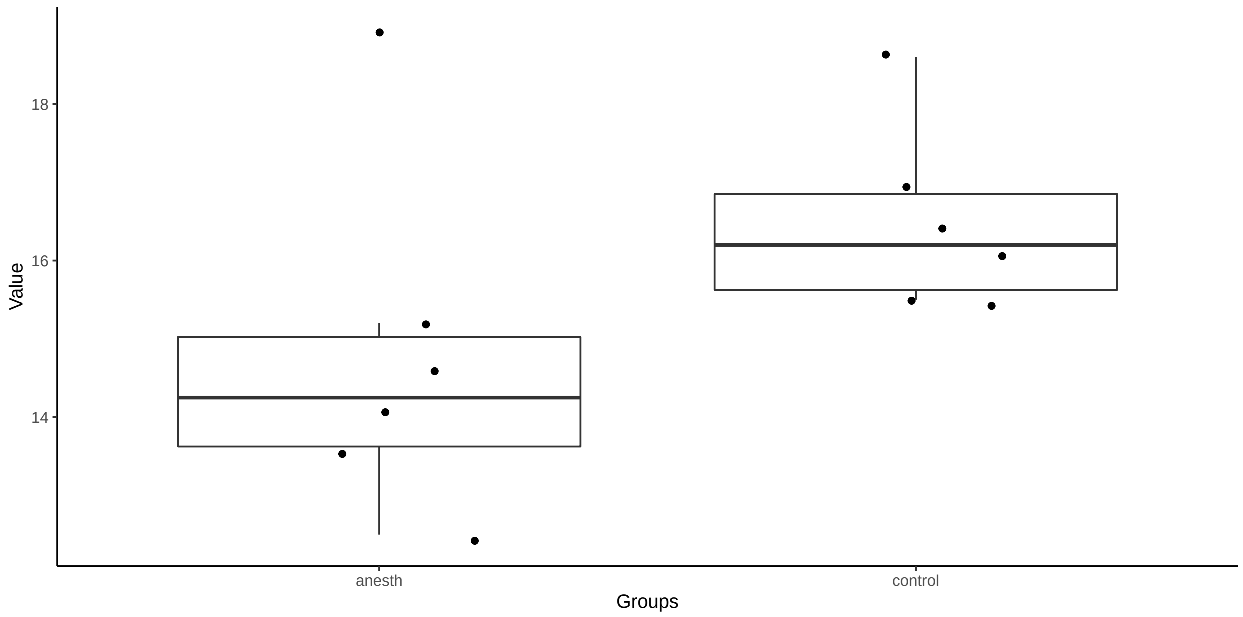


```
## 1. Normality assumption test by Shapiro_Wilk test is
##  p = 0.093
##  Normality assumption was not rejected
## 2. Equal variance test by Bartlett test is
##  p = 0.197
##  Equal variance assumption was not rejected
## 3. The result of anova is
##  p = 0.117
##  A statistically significant difference do not exist between groups
##
```

Data analysis using R

Boohwi Hong

```
## Present data is ** Fig5_C.action_potential_110_Female.csv **
##
## **  Data structure  **
## 'data.frame':    12 obs. of  4 variables:
##  $  ubject      : int  1 2 3 4 5 6 7 8 9 10 ...
##  $ group           : chr  "control" "control" "control" "control" ...
##  $ input.current   : int  110 110 110 110 110 110 110 110 110 110 ...
##  $ action.potential: num  16.5 16 17.5 19.6 15.5 16.6 14 15 13.5 15.8 ...
##
## ** Explorative data analysis with graphics**
```

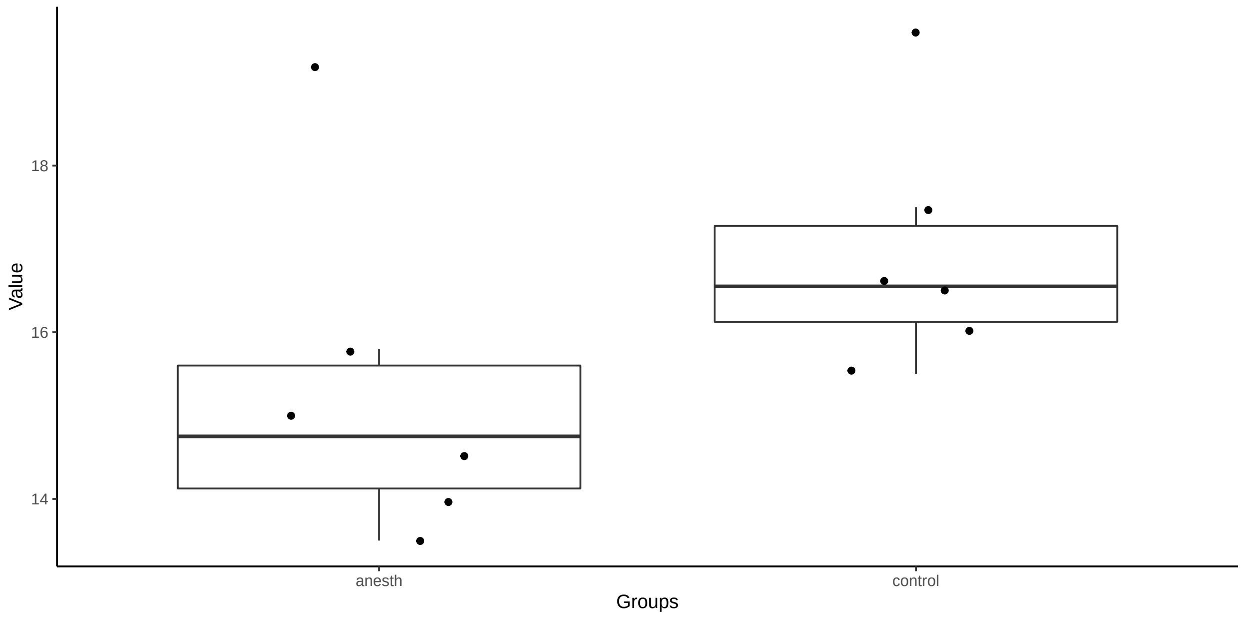


```
## 1. Normality assumption test by Shapiro_Wilk test is
##  p = 0.039
##  Normality assumption was rejected
## 2. The result of Kruskall_Wallis test:
##  p = 0.055
##  A statistically significant difference do not exist between groups
##
```

Data analysis using R

Boohwi Hong

```
## Present data is ** Fig5_D.sEPSC_amplitude_Female.csv **
##
## **  Data structure  **
## 'data.frame':    15 obs. of  4 variables:
##  $ subject  : int  1 2 3 4 5 6 7 8 9 10 ...
##  $ group    : chr  "control" "control" "control" "control" ...
##  $ amplitude: num  18.5 19.2 31.3 15.8 13.1 ...
##  $ X        : logi  NA NA NA NA NA NA ...
##
## ** Explorative data analysis with graphics**
```

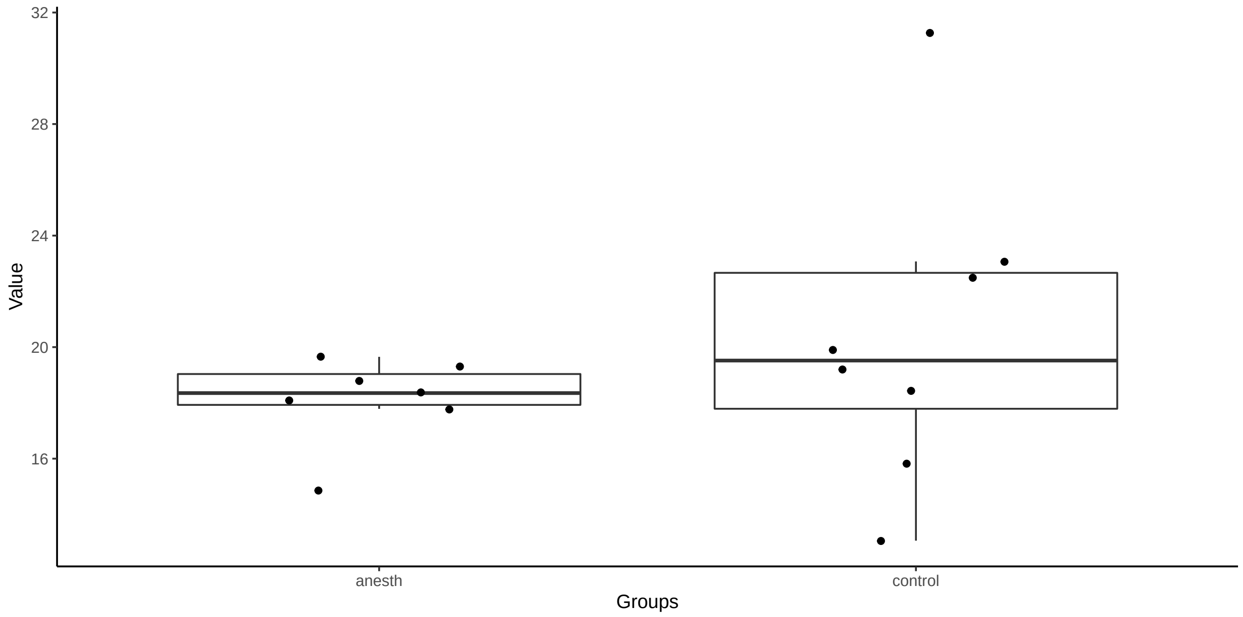


```
## 1. Normality assumption test by Shapiro_Wilk test is
##  p = 0.092
##  Normality assumption was not rejected
## 2. Equal variance test by Bartlett test is
##  p = 0.007
##  Equal variance assumption was rejected
## 3. The result of Welch ANOVA is
##  p = 0.290
##  A statistically significant difference do not exist between groups
##
```

Data analysis using R

Boohwi Hong

```
## Present data is ** Fig5_D.sEPSC_freq_Female.csv **
##
## **  Data structure  **
## 'data.frame':    15 obs. of  3 variables:
##  $ subject: int  1 2 3 4 5 6 7 8 9 10 ...
##  $ group  : chr  "control" "control" "control" "control" ...
##  $ freq   : num  0.175 0.329 0.267 0.515 0.25 ...
##
## ** Explorative data analysis with graphics**
```

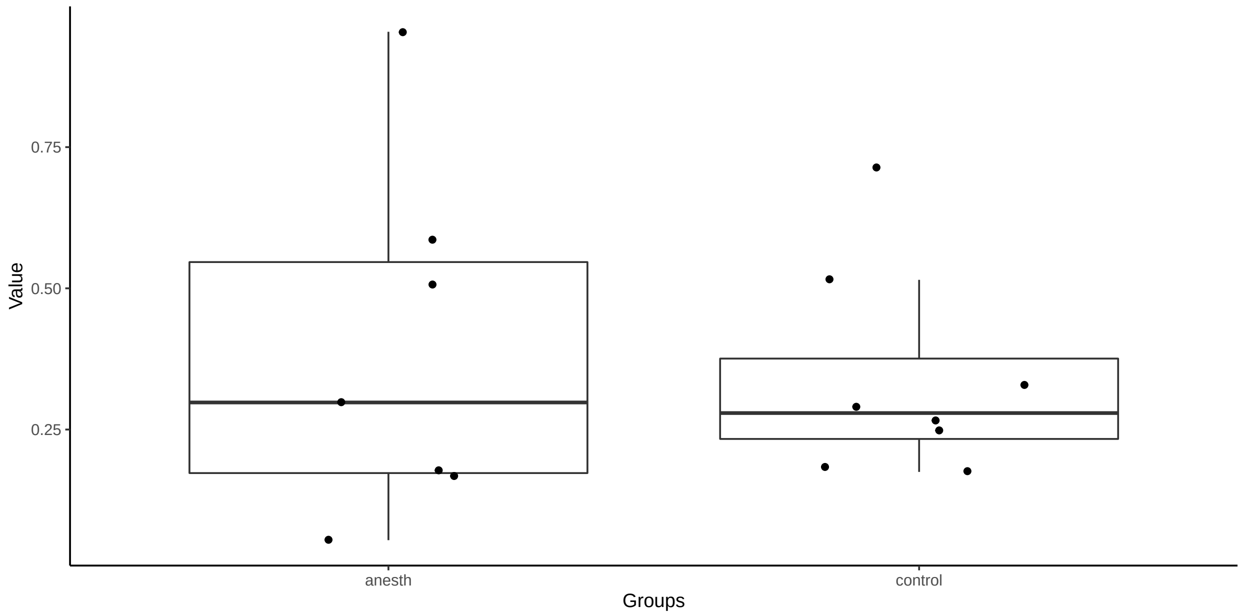


```
## 1. Normality assumption test by Shapiro_Wilk test is
##  p = 0.227
##  Normality assumption was not rejected
## 2. Equal variance test by Bartlett test is
##  p = 0.199
##  Equal variance assumption was not rejected
## 3. The result of anova is
##  p = 0.698
##  A statistically significant difference do not exist between groups
##
```

Data analysis using R

Boohwi Hong

```
## Present data is ** Fig5_E.sIPSC_amplitude_Female.csv **
##
## **  Data structure  **
## 'data.frame':    15 obs. of  4 variables:
##  $ subject  : int  1 2 3 4 5 6 7 8 9 10 ...
##  $ group    : chr  "control" "control" "control" "control" ...
##  $ amplitude: num  56 47 49.8 34.1 38.4 ...
##  $ X        : logi  NA NA NA NA NA NA ...
##
## ** Explorative data analysis with graphics**
```

```
## Warning: Removed 2 rows containing non-finite
## values (stat_boxplot).
```

```
## Warning: Removed 2 rows containing missing
## values (geom_point).
```

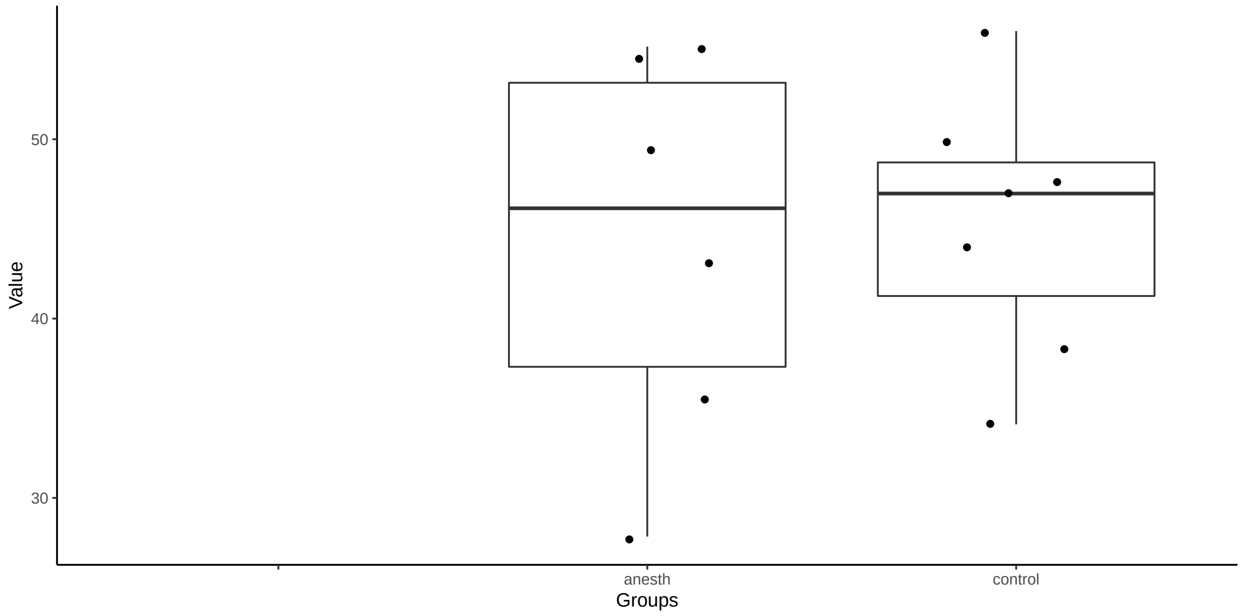


```
## 1. Normality assumption test by Shapiro_Wilk test is
##  p = 0.511
##  Normality assumption was not rejected
## 2. Equal variance test by Bartlett test is
##  p = 0.365
##  Equal variance assumption was not rejected
```

```
## 3. The result of anova is
##  p = 0.833
##  A statistically significant difference do not exist between groups
##
```

Data analysis using R

Boohwi Hong

```
## Present data is ** Fig5_E.sIPSC_freq_Female.csv **
##
## **  Data structure  **
## 'data.frame':    13 obs. of  4 variables:
##  $ subject: int  1 2 3 4 5 6 7 8 9 10 ...
##  $ group  : chr  "control" "control" "control" "control" ...
##  $ freq   : num  6.38 5.29 8.6 9.28 4.53 ...
##  $ X      : logi  NA NA NA NA NA NA ...
##
## ** Explorative data analysis with graphics**
```

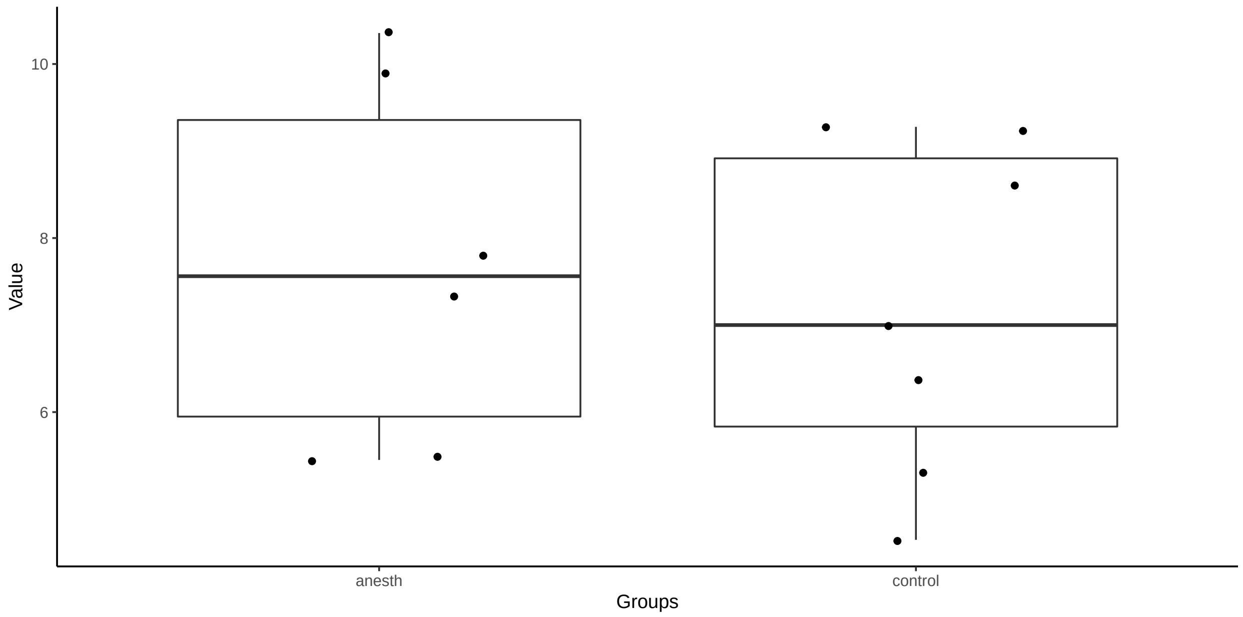


```
## 1. Normality assumption test by Shapiro_Wilk test is
##  p = 0.155
##  Normality assumption was not rejected
## 2. Equal variance test by Bartlett test is
##  p = 0.838
##  Equal variance assumption was not rejected
## 3. The result of anova is
##  p = 0.643
##  A statistically significant difference do not exist between groups
##
```

Data analysis using R

Boohwi Hong

```
## Present data is ** Fig5_F.sAP_freq_Female.csv **
##
## **  Data structure  **
## 'data.frame':    13 obs. of  4 variables:
##  $ subject: int  1 2 3 4 5 6 7 8 9 10 ...
##  $ group  : chr  "control" "control" "control" "control" ...
##  $ freq   : num  0 0 0 0 0 0.000333 0.000333 0 0 0.000833 ...
##  $ X      : logi  NA NA NA NA NA NA ...
##
## ** Explorative data analysis with graphics**
```

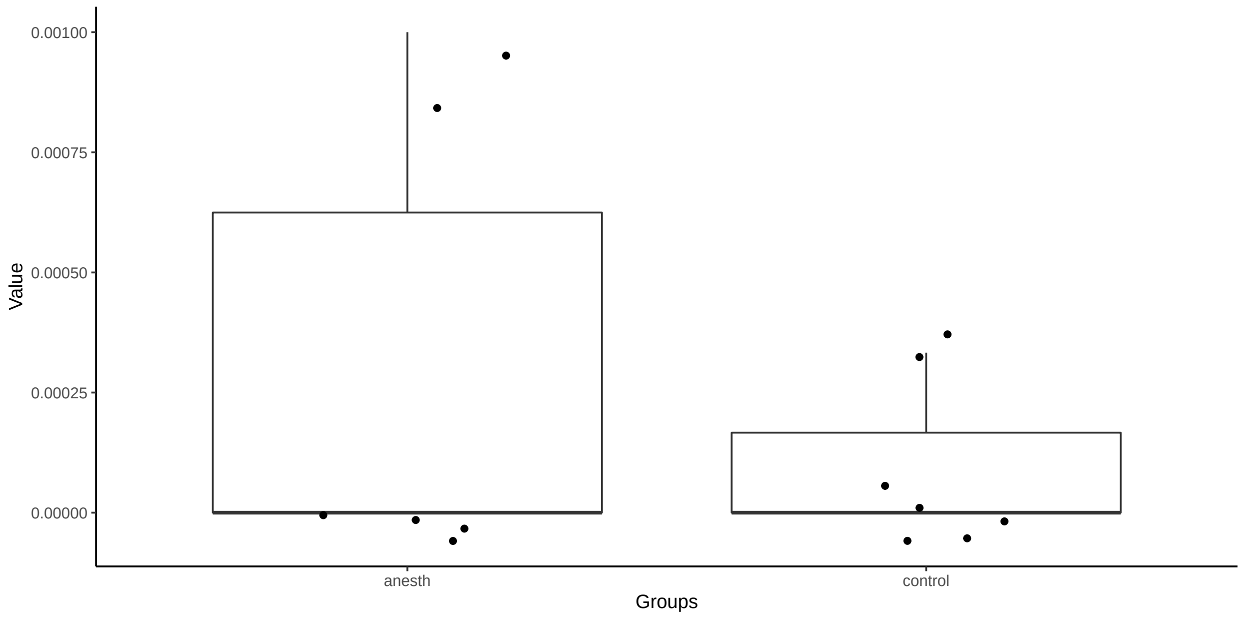


```
## 1. Normality assumption test by Shapiro_Wilk test is
##  p = 0.017
##  Normality assumption was rejected
## 2. The result of Kruskall_Wallis test:
##  p = 0.600
##  A statistically significant difference do not exist between groups
##
```

Supplementary Note 6

The results of statistical analysis
for Supplementary Figure 1

Suppl_Fig1A.CPP_Control_Male Data analysis using R

Boohwi Hong

Package install

Data import

Data structure

```
str(d1)
```

```
## 'data.frame':    34 obs. of  3 variables:
##  $ subject : int  1 2 3 4 5 6 7 8 9 10 ...
##  $ group   : chr  "pre" "pre" "pre" "pre" ...
##  $ duration: num  305 296 369 265 285 ...
```

Explorative data analysis with graphics

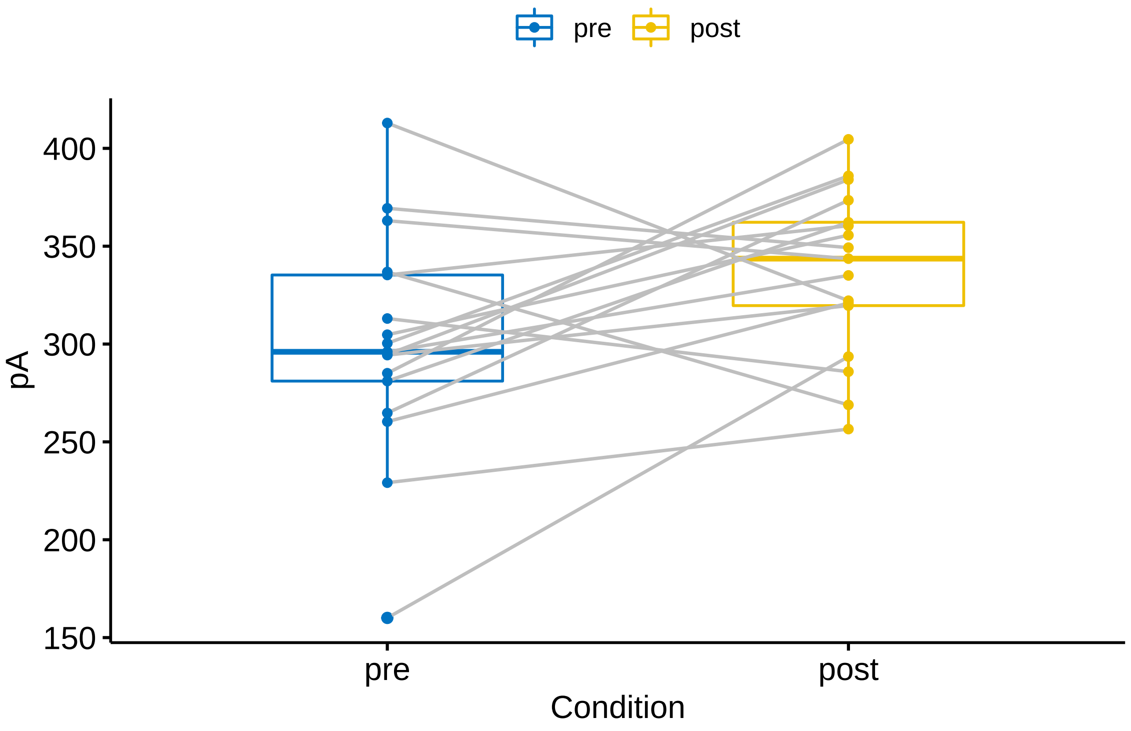

Easystat function for paired_data developed by S. Park & B. Hong (available at https://rpubs.com/koho0127)

Statistical Result

```
paired_easystat(d1)
```

```
## 1. Normality assumption test by Shapiro_Wilk test is
##  p = 0.791
##  Normality assumption was not rejected
## 2. The result of paired t-test is
##  p = 0.034
##  A statistically significant difference exist between groups
```

Suppl_Fig1A.CPP_Anesth_Male Data analysis using R

Boohwi Hong

Package install

Data import

Data structure

```
str(d1)
```

```
## 'data.frame':    34 obs. of  3 variables:
##  $ subject : int  1 2 3 4 5 6 7 8 9 10 ...
##  $ group   : chr  "pre" "pre" "pre" "pre" ...
##  $ duration: num  321 384 216 216 227 ...
```

Explorative data analysis with graphics

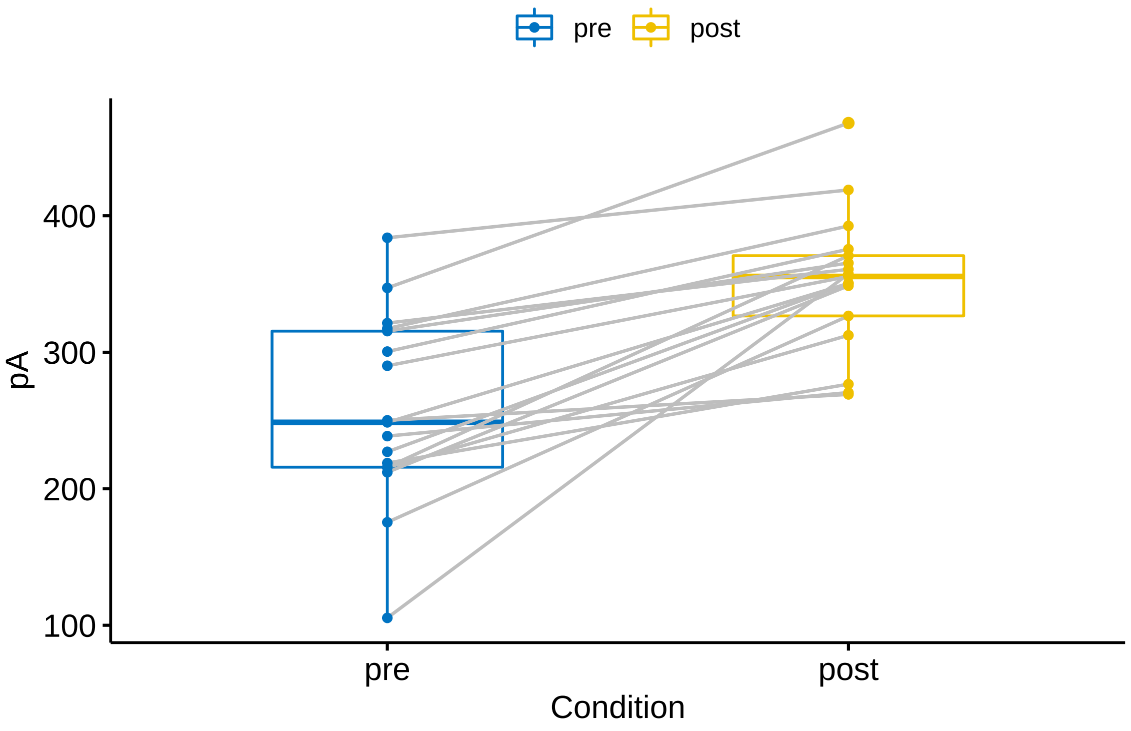

Easystat function for paired_data developed by S. Park & B. Hong (available at https://rpubs.com/koho0127)

Statistical Result

```
paired_easystat(d1)
```

```
## 1. Normality assumption test by Shapiro_Wilk test is
##  p = 0.928
##  Normality assumption was not rejected
## 2. The result of paired t-test is
##  p = 0.000
##  A statistically significant difference exist between groups
```

Suppl_Fig1B.CPP score_Male Data analysis using R

Boohwi Hong

Package install

Data import

Data structure

```
str(d1)
```

```
## 'data.frame':    34 obs. of  3 variables:
##  $ subject  : int  1 2 3 4 5 6 7 8 9 10 ...
##  $ group    : chr  "Cont" "Cont" "Cont" "Cont" ...
##  $ CPP.score: num  50.9 39 -20.1 108.7 119.5 ...
```

Explorative data analysis with graphics

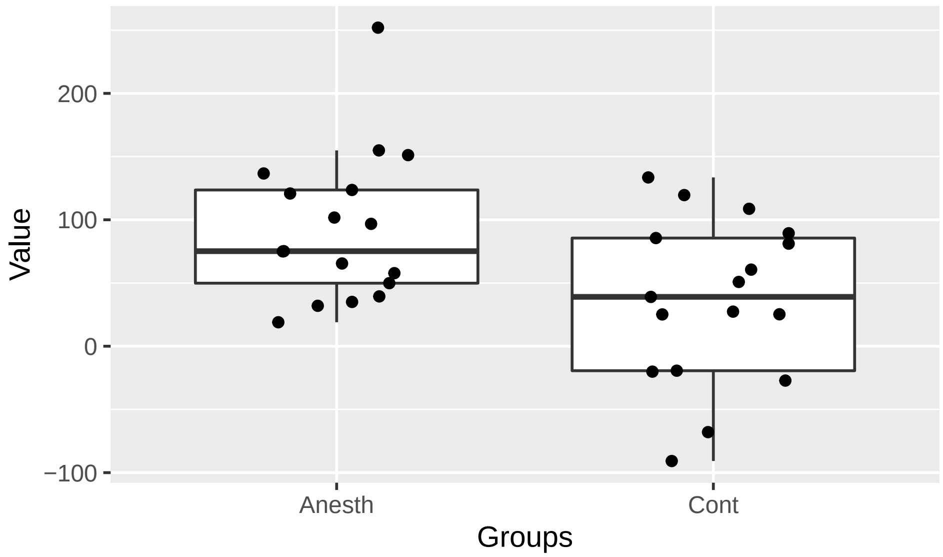

Easystat function developed by S. Park (available at https://rpubs.com/goodlebang)

Statistical Result

```
easystat(d1)
```

```
## 1. Normality assumption test by Shapiro_Wilk test is
##  p = 0.96
##  Normality assumption was not rejected
## 2. Equal variance test by Bartlett test is
##  p = 0.720
##  Equal variance assumption was not rejected
## 3. The result of anova is
##  p = 0.012
##  A statistically significant difference exist between groups
```

```
##   Tukey multiple comparisons of means
##     95% family-wise confidence level
##
## Fit: aov(formula = d1[, 3] ~ d1[, 2], data = d1)
##
## $`d1[, 2]`
##                  diff       lwr       upr     p adj
## Cont-Anesth -56.80029 -100.1968 -13.40377 0.0119312
```

Supplementary Note 7

The results of statistical analysis
for Supplementary Figure 2

Suppl_Fig2_B.keta-CPP_anesth_male(Adult)Data analysis using R

Boohwi Hong

Statistical Result

```
## Present data is ** Suppl_Fig2_B.keta-CPP_anesth_male(Adult).csv ** ##
## **  Data structure  **
## 'data.frame':    64 obs. of  3 variables:
## $  ubjectint 1 2 3 4 5 6 7 8 9 10 ...
##  $ group     : chr  "pre" "pre" "pre" "pre" ...
##  $ duration  : num  312 322 274 375 274 ...
##
## ** Explorative data analysis with graphics**
```

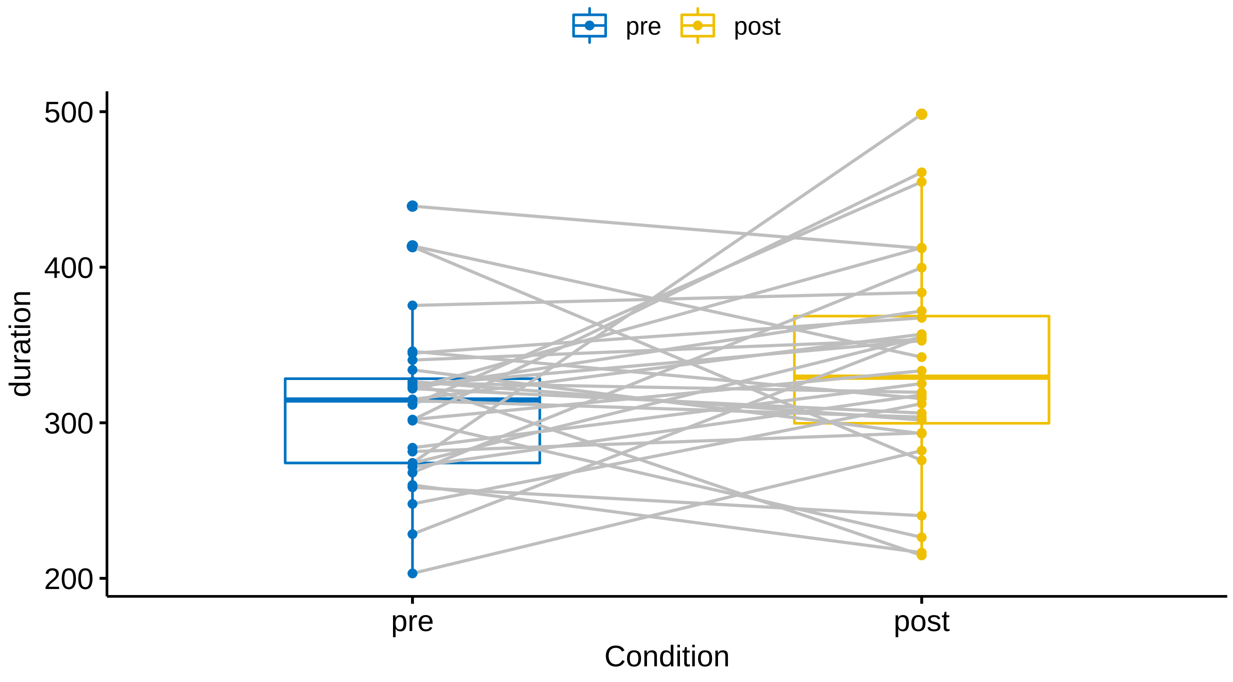


```
## 1. Normality assumption test by Shapiro_Wilk test is
##  p = 0.153
##  Normality assumption was not rejected
## 2. The result of paired t-test is
##  p = 0.091
##  A statistically significant difference do not exist between groups
```

Suppl_Fig2_B.keta-CPP_control_male(Adult)Data analysis using R

Boohwi Hong

Statistical Result

```
## Present data is ** Suppl_Fig2_B.keta-CPP_control_male(Adult).csv ** ##
## **  Data structure  **
## 'data.frame':    64 obs. of  3 variables:
## $  ubjectint 1 2 3 4 5 6 7 8 9 10 ...
##  $ group     : chr  "pre" "pre" "pre" "pre" ...
##  $ duration  : num  270 256 236 352 372 ...
##
## ** Explorative data analysis with graphics**
```

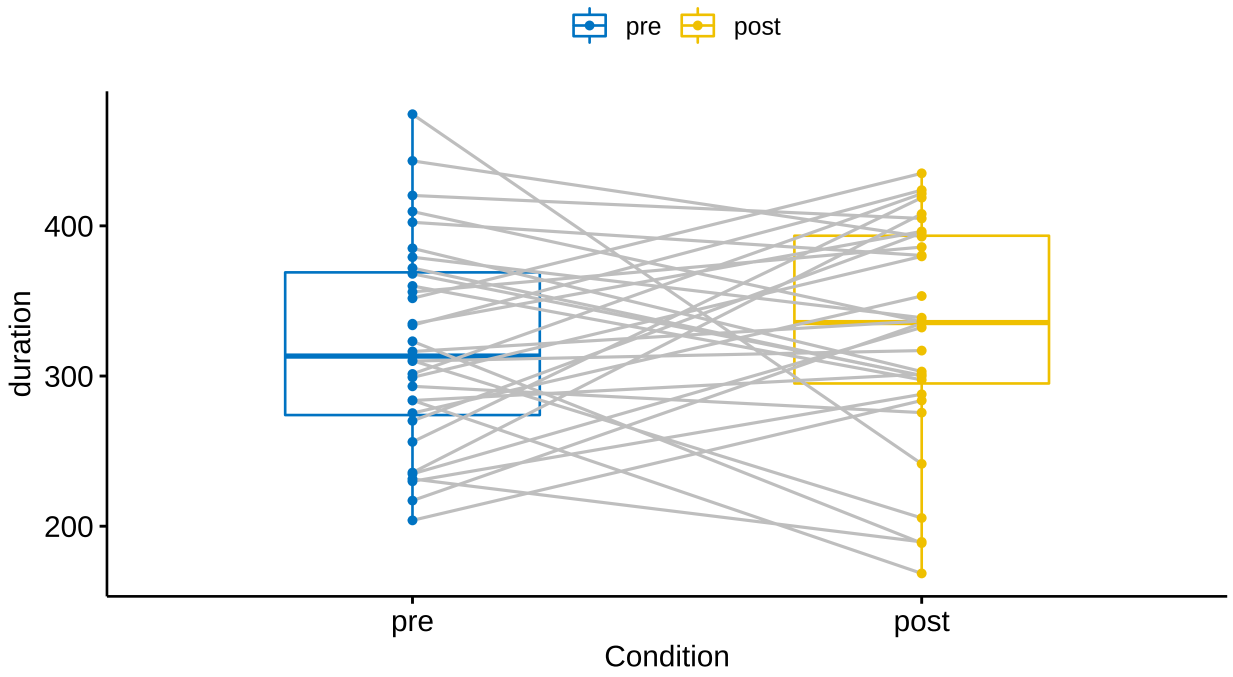


```
## 1. Normality assumption test by Shapiro_Wilk test is
##  p = 0.593
##  Normality assumption was not rejected
## 2. The result of paired t-test is
##  p = 0.620
##  A statistically significant difference do not exist between groups
```

Suppl_Fig2_C.keta-CPP_CPPscore_male(Adult)Data analysis using R

Boohwi Hong

```
## Present data is ** Suppl_Fig2_C.keta-CPP_CPPscore_male(Adult).csv ** ##
## **  Data structure  **
## 'data.frame':    64 obs. of  3 variables:

##  $  ubject: int  1 2 3 4 5 6 7 8 9 10 ...
##  $ group     : chr  "control" "control" "control" "control" ...
##  $ difference: num  125.1 162.6 172.2 83.2 -71.9 ...
##
## ** Explorative data analysis with graphics**
```

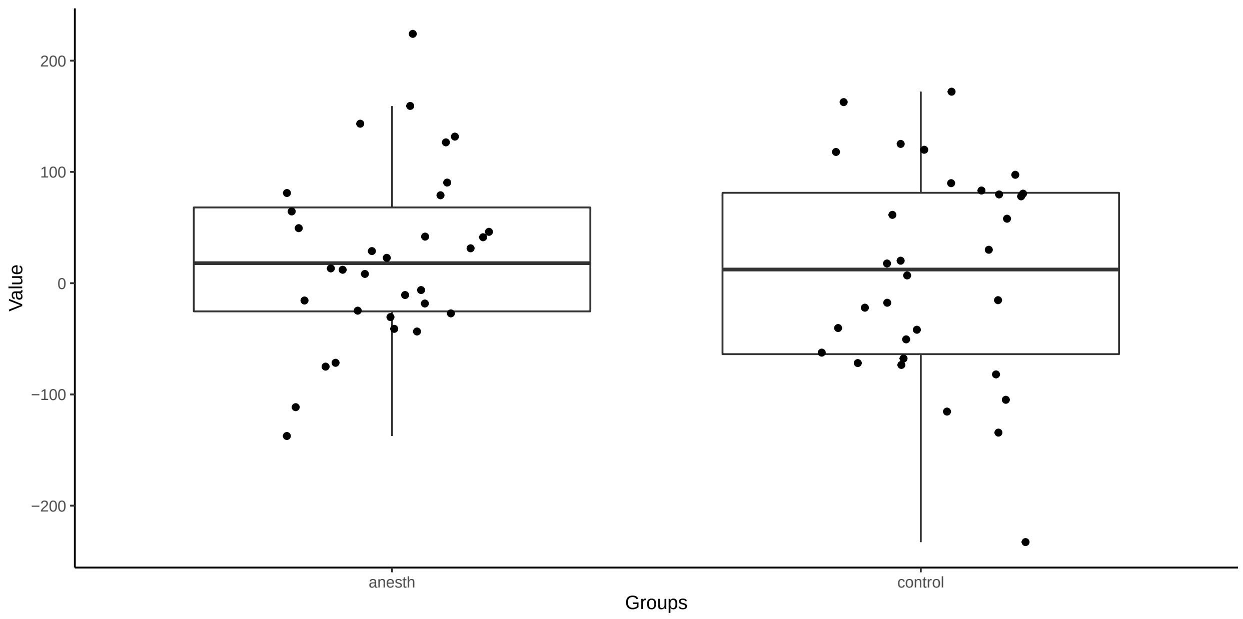


```
## 1. Normality assumption test by Shapiro_Wilk test is
##  p = 0.986
##  Normality assumption was not rejected
## 2. Equal variance test by Bartlett test is
##  p = 0.321
##  Equal variance assumption was not rejected
## 3. The result of anova is
##  p = 0.465
##  A statistically significant difference do not exist between groups
##
```

Supplementary Note 8

The results of statistical analysis for Supplementary Figure 3

Suppl_Fig3_A.NAcc_DA_MaleData analysis using R

Boohwi Hong

```
## Present data is ** Suppl_Fig3_A.NAcc_DA_Male.csv ** ##
## **  Data structure  **

## 'data.frame':    18 obs. of  3 variables:
##  $  ubject: int  1 2 3 4 5 6 7 8 9 10 ...
##  $ group    : chr  "control" "control" "control" "control" ...
##  $ value    : num  21.8 26.9 13.4 13.9 21.7 ...
##
## ** Explorative data analysis with graphics**
```

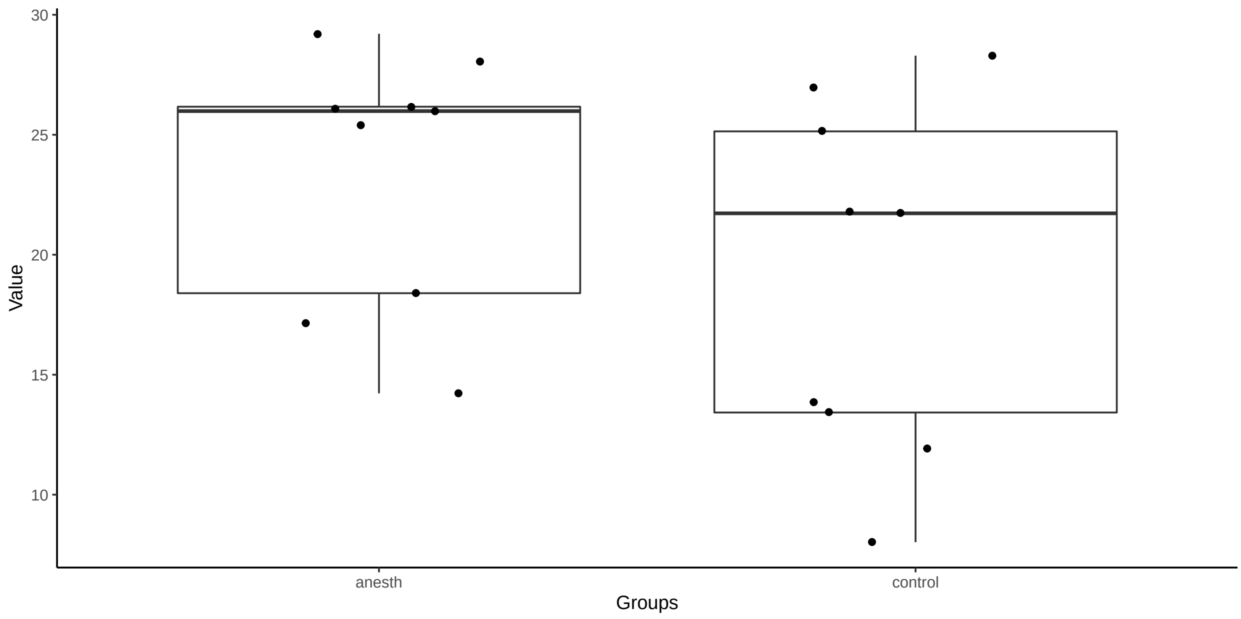


```
## 1. Normality assumption test by Shapiro_Wilk test is
##  p = 0.1
##  Normality assumption was not rejected
## 2. Equal variance test by Bartlett test is
##  p = 0.391
##  Equal variance assumption was not rejected
## 3. The result of anova is
##  p = 0.166
##  A statistically significant difference do not exist between groups
##
```

Suppl_Fig3_A.VTA_DA_MaleData analysis using R

Boohwi Hong

```
## Present data is ** Suppl_Fig3_A.VTA_DA_Male.csv **
##
## **  Data structure  **
## 'data.frame':    16 obs. of  3 variables:
##  $  ubject: int  1 2 3 4 5 6 7 8 9 10 ...
##  $ group     : chr  "control" "control" "control" "control" ...
##  $ value     : num  8.24 1.19 6.39 1.83 1.82 ...
##
## ** Explorative data analysis with graphics**
```

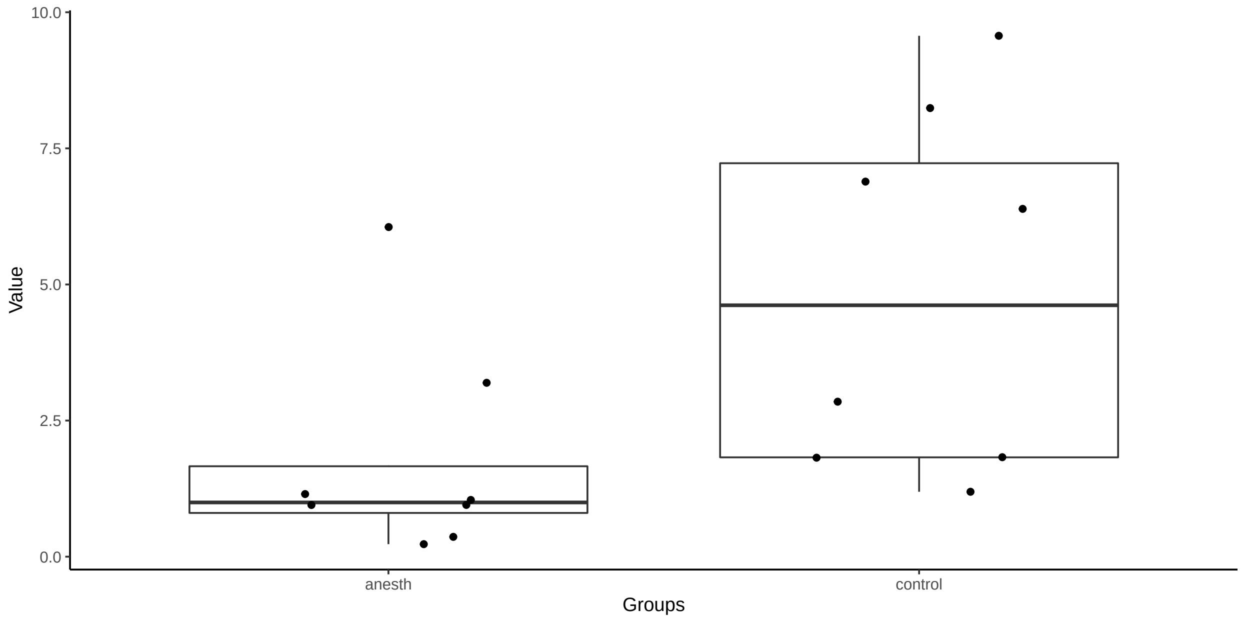


```
## 1. Normality assumption test by Shapiro_Wilk test is
##  p = 0.25
##  Normality assumption was not rejected
## 2. Equal variance test by Bartlett test is
##  p = 0.195
##  Equal variance assumption was not rejected
## 3. The result of anova is
##  p = 0.038
##  A statistically significant difference exist between groups
##
##   Tukey multiple comparisons of means
##     95% family-wise confidence level
##
```

```
## Fit: aov(formula = d1[, 3] ~ d1[, 2], data = d1)
##
## $`d1[, 2]`
##                    diff       lwr      upr    p adj
## control-anesth 3.104411 0.1958687 6.012953 0.038121
```

Suppl_Fig3_A.Striatum_DA_MaleData analysis using R

Boohwi Hong

```
## Present data is ** Suppl_Fig3_A.Striatum_DA_Male.csv ** ##
## **  Data structure  **

## 'data.frame':    18 obs. of  3 variables:
##  $  ubject: int  1 2 3 4 5 6 7 8 9 10 ...
##  $ group     : chr  "control" "control" "control" "control" ...
##  $ value     : num  45 64.6 42 41.7 55.1 ...
##
## ** Explorative data analysis with graphics**
```

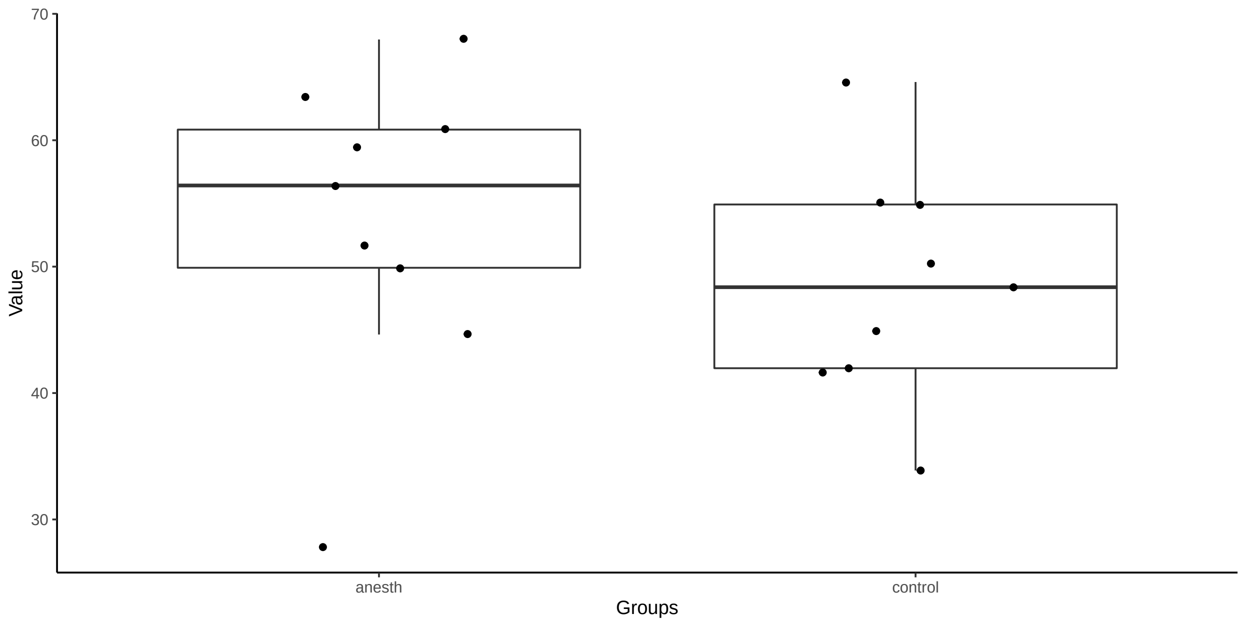


```
## 1. Normality assumption test by Shapiro_Wilk test is
##  p = 0.658
##  Normality assumption was not rejected
## 2. Equal variance test by Bartlett test is
##  p = 0.445
##  Equal variance assumption was not rejected
## 3. The result of anova is
##  p = 0.321
##  A statistically significant difference do not exist between groups
##
```

Suppl_Fig3_B.NAcc_DOPAC_MaleData analysis using R

Boohwi Hong

```
## Present data is ** Suppl_Fig3_B.NAcc_DOPAC_Male.csv ** ##
## **  Data structure  **
## 'data.frame':    18 obs. of  3 variables:

##  $  ubject: int  1 2 3 4 5 6 7 8 9 10 ...
##  $ group     : chr  "control" "control" "control" "control" ...
##  $ value     : num  4.28 5.25 5.36 8.26 6.53 ...
##
## ** Explorative data analysis with graphics**
```

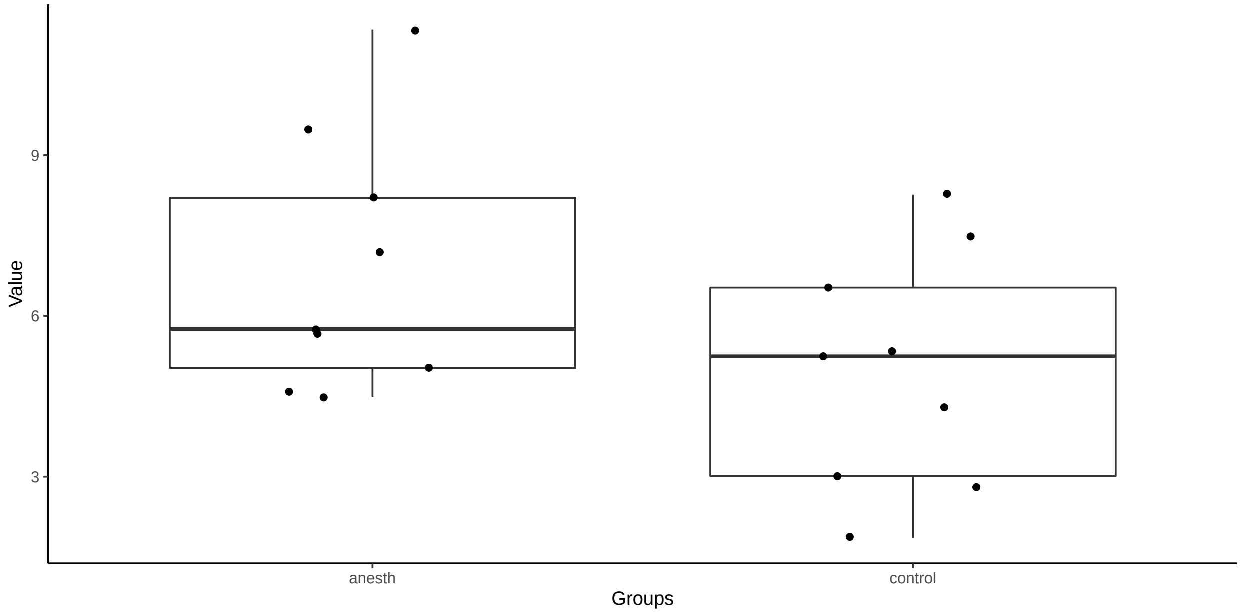


```
## 1. Normality assumption test by Shapiro_Wilk test is
##  p = 0.328
##  Normality assumption was not rejected
## 2. Equal variance test by Bartlett test is
##  p = 0.814
##  Equal variance assumption was not rejected
## 3. The result of anova is
##  p = 0.101
##  A statistically significant difference do not exist between groups
##
```

Suppl_Fig3_B.VTA_DOPAC_MaleData analysis using R

Boohwi Hong

```
## Present data is ** Suppl_Fig3_B.VTA_DOPAC_Male.csv ** ##
## **  Data structure  **

## 'data.frame':    17 obs. of  3 variables:
##  $  ubject: int  1 2 3 4 5 6 7 8 9 10 ...
##  $ group    : chr  "control" "control" "control" "control" ...
##  $ value    : num  1.875 0.155 1.255 0.62 0.524 ...
##
## ** Explorative data analysis with graphics**
```

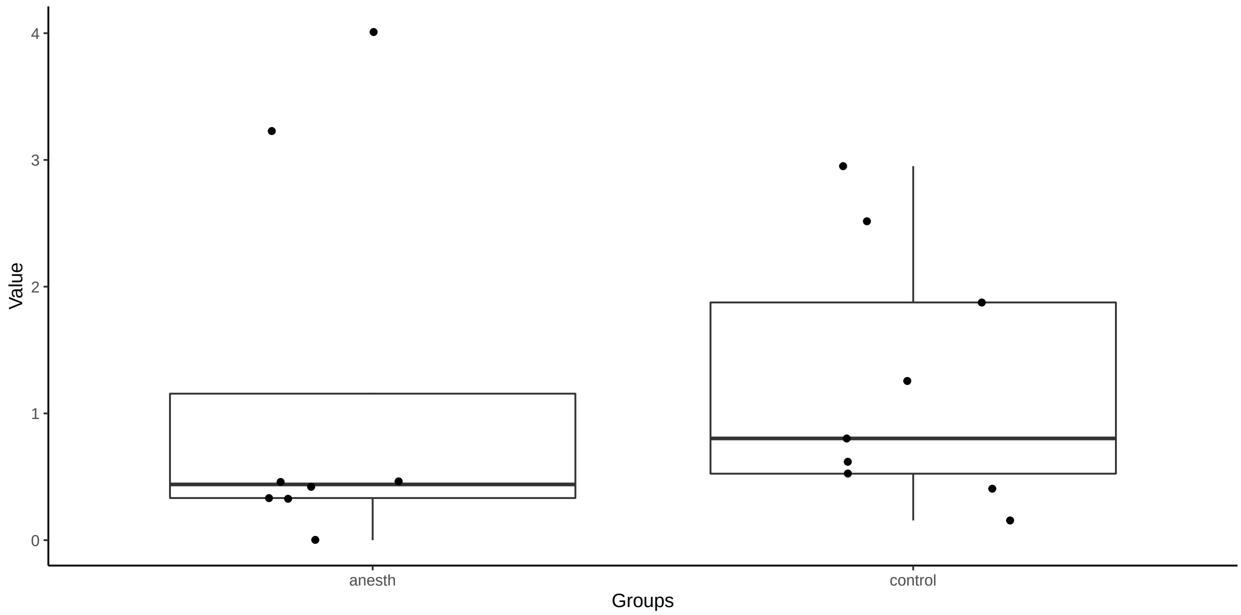


```
## 1. Normality assumption test by Shapiro_Wilk test is
##  p = 0.002
##  Normality assumption was rejected
## 2. The result of Kruskall_Wallis test:
##  p = 0.336
##  A statistically significant difference do not exist between groups
##
```

Suppl_Fig3_B.striatum_DOPAC_MaleData analysis using R

Boohwi Hong

```
## Present data is ** Suppl_Fig3_B.striatum_DOPAC_Male.csv ** ##
## **  Data structure  **

## 'data.frame':    18 obs. of  3 variables:
##  $  ubject: int  1 2 3 4 5 6 7 8 9 10 ...
##  $ group     : chr  "control" "control" "control" "control" ...
##  $ value     : num  10.54 11.2 11.05 8.99 10.36 ...
##
## ** Explorative data analysis with graphics**
```

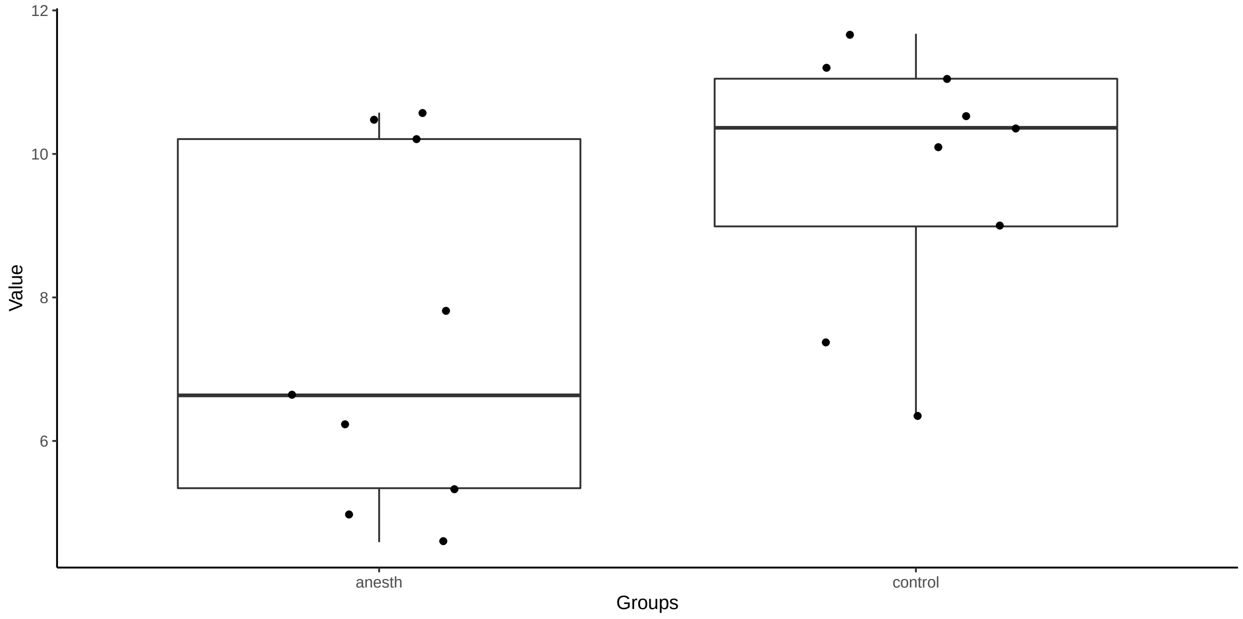


```
## 1. Normality assumption test by Shapiro_Wilk test is
##  p = 0.422
##  Normality assumption was not rejected
## 2. Equal variance test by Bartlett test is
##  p = 0.418
##  Equal variance assumption was not rejected
## 3. The result of anova is
##  p = 0.037
##  A statistically significant difference exist between groups
##
##   Tukey multiple comparisons of means
##     95% family-wise confidence level
##
```

```
## Fit: aov(formula = d1[, 3] ~ d1[, 2], data = d1)
##
## $`d1[, 2]`
##                    diff    lwr     upr    p adj
## control-anesth 2.311935 0.1618 4.46207 0.036698
```

Supplementary Note 9

The results of statistical analysis
for Supplementary Figure 4

Data analysis using R

Boohwi Hong

```
## Present data is ** Suppl_Fig4.A_RMP_Male.csv **
##
## **  Data structure  **
## 'data.frame':    13 obs. of  3 variables:
##  $  ubject: int  1 2 3 4 5 6 7 8 9 10 ...
##  $ group     : chr  "control" "control" "control" "control" ...
##  $ RMP       : num  -65 -65 -67 -69.5 -62.7 ...
##
## ** Explorative data analysis with graphics**
```

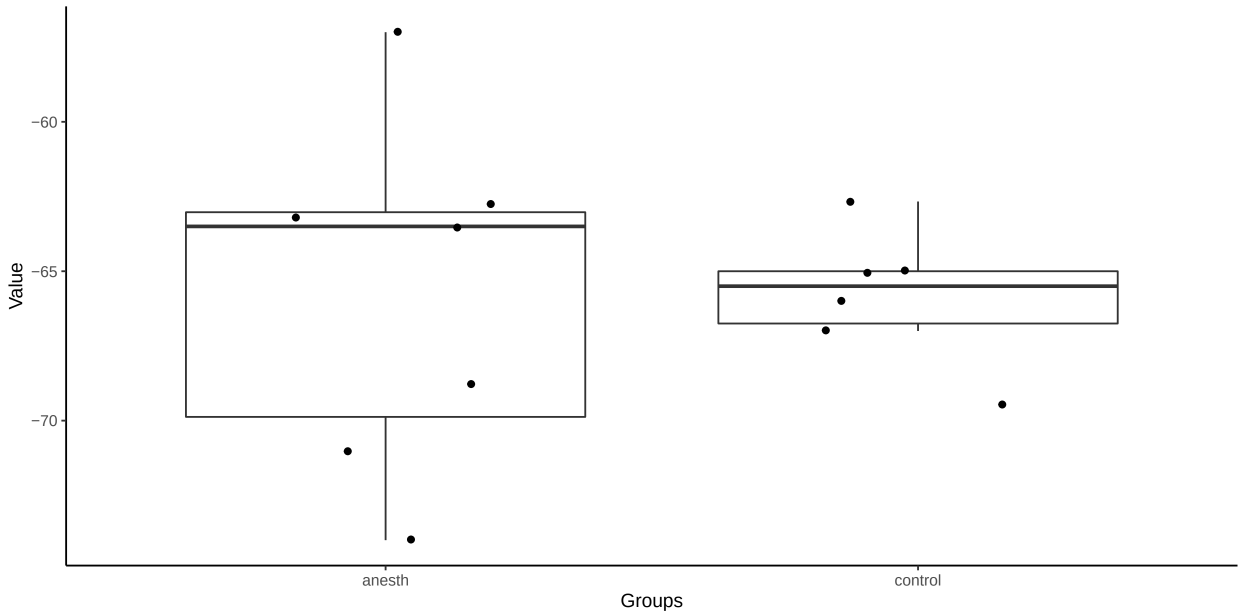


```
## 1. Normality assumption test by Shapiro_Wilk test is
##  p = 0.921
##  Normality assumption was not rejected
## 2. Equal variance test by Bartlett test is
##  p = 0.057
##  Equal variance assumption was not rejected
## 3. The result of anova is
##  p = 0.968
##  A statistically significant difference do not exist between groups
##
```

Data analysis using R

Boohwi Hong

```
## Present data is ** Suppl_Fig4.B_Threshold_Male.csv **
##
## **  Data structure  **
## 'data.frame':    13 obs. of  3 variables:
##  $  ubject: int  1 2 3 4 5 6 7 8 9 10 ...
##  $ group     : chr  "control" "control" "control" "control" ...
##  $ Threshold : num  -42.5 -40.3 -37.3 -55.9 -51.1 ...
##
## ** Explorative data analysis with graphics**
```

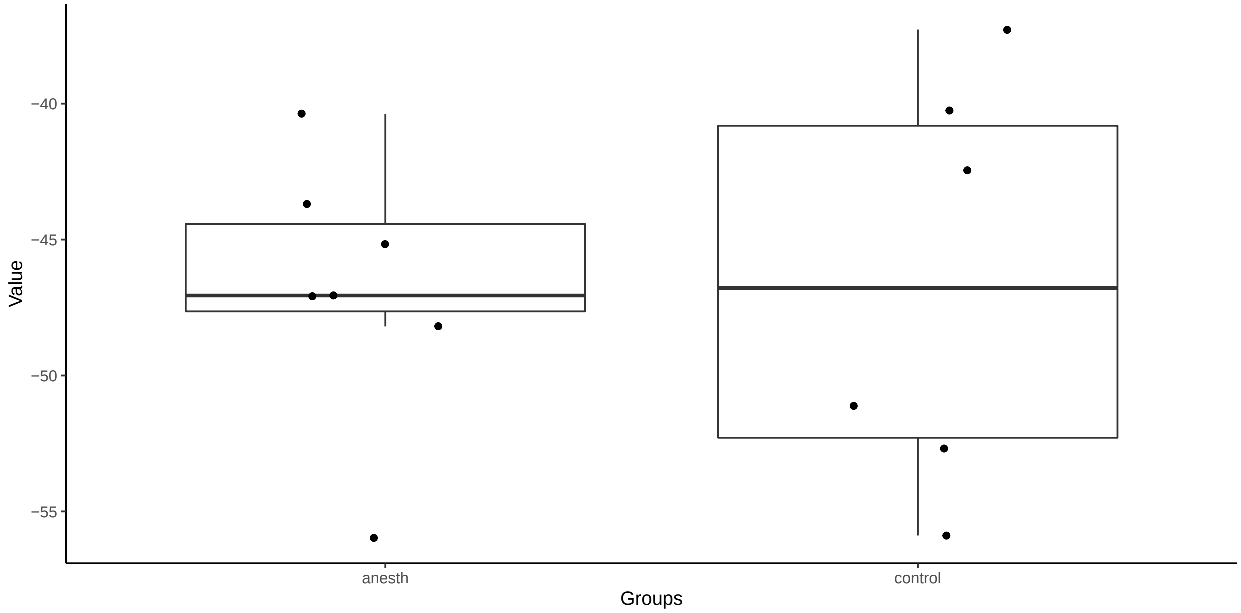


```
## 1. Normality assumption test by Shapiro_Wilk test is
##  p = 0.706
##  Normality assumption was not rejected
## 2. Equal variance test by Bartlett test is
##  p = 0.314
##  Equal variance assumption was not rejected
## 3. The result of anova is
##  p = 0.959
##  A statistically significant difference do not exist between groups
##
```

Data analysis using R

Boohwi Hong

```
## Present data is ** Suppl_Fig4.C_Input Res_Male.csv **
##
## **  Data structure  **
## 'data.frame':    13 obs. of  3 variables:
##  $ subject  : int  1 2 3 4 5 6 7 8 9 10 ...
##  $ group    : chr  "control" "control" "control" "control" ...
##  $ Input.Res: num  207 204 188 294 211 ...
##
## ** Explorative data analysis with graphics**
```

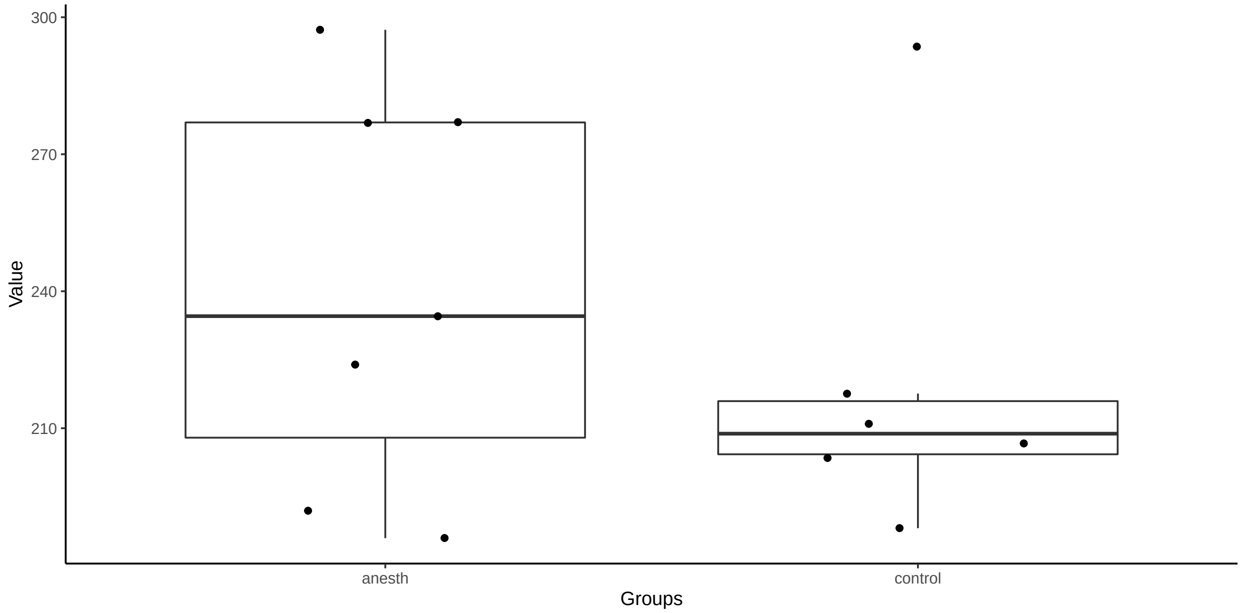


```
## 1. Normality assumption test by Shapiro_Wilk test is
##  p = 0.365
##  Normality assumption was not rejected
## 2. Equal variance test by Bartlett test is
##  p = 0.721
##  Equal variance assumption was not rejected
## 3. The result of anova is
##  p = 0.377
##  A statistically significant difference do not exist between groups
##
```

Supplementary Note 10

The results of statistical analysis
for Supplementary Figure 5

Data analysis using R

Boohwi Hong

```
## Present data is ** Suppl_Fig5.B_mEPSC_amp_Male.csv **
##
## **  Data structure  **
## 'data.frame':    12 obs. of  3 variables:
##  $ subject: int  1 2 3 4 5 6 7 8 9 10 ...
##  $ group  : chr  "control" "control" "control" "control" ...
##  $ amp    : num  46.1 36.8 28.8 20.5 19.5 ...
##
## ** Explorative data analysis with graphics**
```

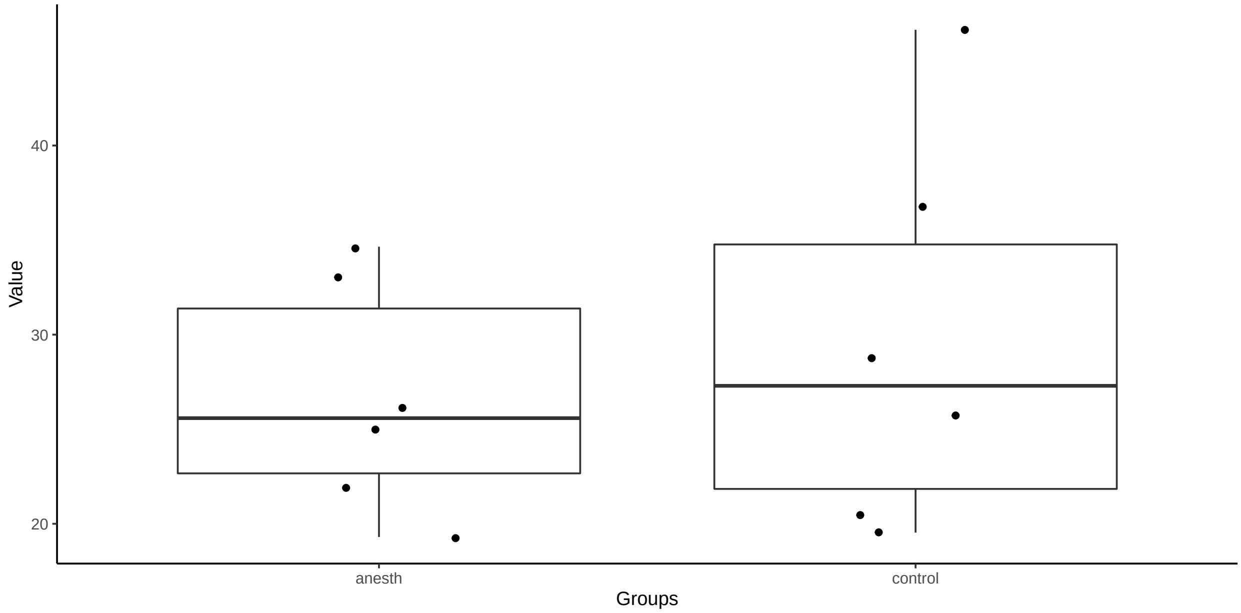


```
## 1. Normality assumption test by Shapiro_Wilk test is
##  p = 0.443
##  Normality assumption was not rejected
## 2. Equal variance test by Bartlett test is
##  p = 0.279
##  Equal variance assumption was not rejected
## 3. The result of anova is
##  p = 0.564
##  A statistically significant difference do not exist between groups
##
```

Data analysis using R

Boohwi Hong

```
## Present data is ** Suppl_Fig5.B_mEPSC_freq_Male.csv **
##
## **  Data structure  **
## 'data.frame':    12 obs. of  3 variables:
##  $ subject: int  1 2 3 4 5 6 7 8 9 10 ...
##  $ group  : chr  "control" "control" "control" "control" ...
##  $ freq   : num  0.01667 0.00417 0.00833 0.04792 0.01667 ...
##
## ** Explorative data analysis with graphics**
```

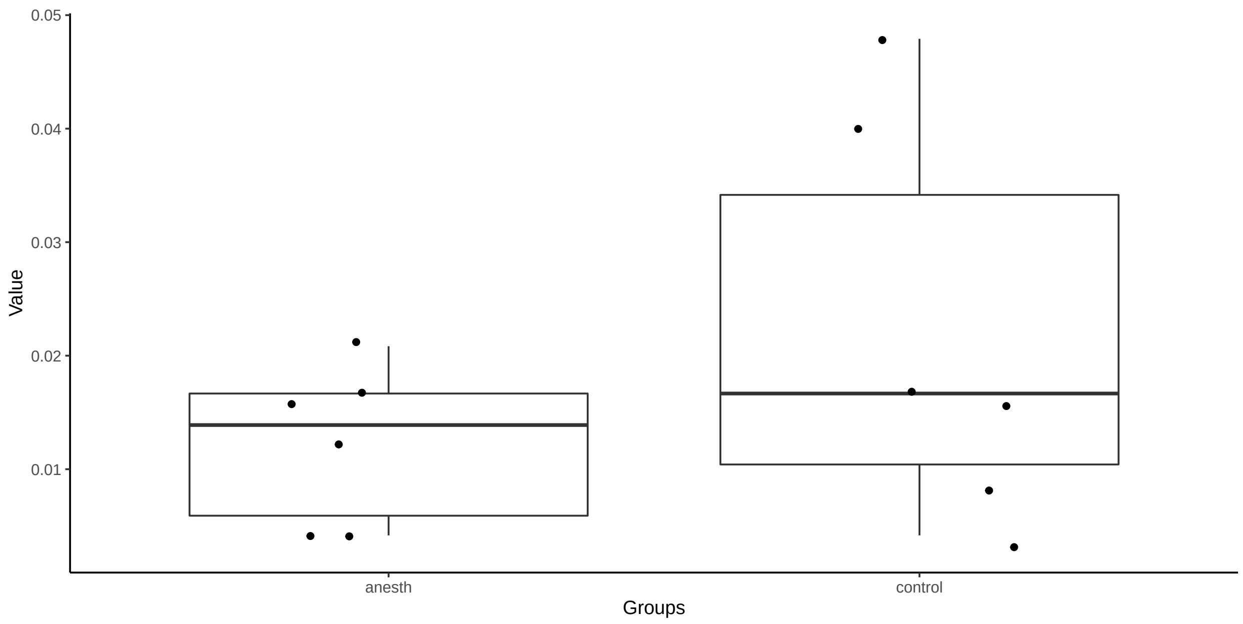


```
## 1. Normality assumption test by Shapiro_Wilk test is
##  p = 0.684
##  Normality assumption was not rejected
## 2. Equal variance test by Bartlett test is
##  p = 0.064
##  Equal variance assumption was not rejected
## 3. The result of anova is
##  p = 0.225
##  A statistically significant difference do not exist between groups
##
```

Data analysis using R

Boohwi Hong

```
## Present data is ** Suppl_Fig5.D_mIPSC_amp_Male.csv **
##
## **  Data structure  **
## 'data.frame':    13 obs. of  4 variables:
##  $ subject: int  1 2 3 4 5 6 7 8 9 10 ...
##  $ group  : chr  "control" "control" "control" "control" ...
##  $ amp    : num  32.9 29.9 31.5 32.8 36.1 ...
##  $ X      : logi  NA NA NA NA NA NA ...
##
## ** Explorative data analysis with graphics**
```

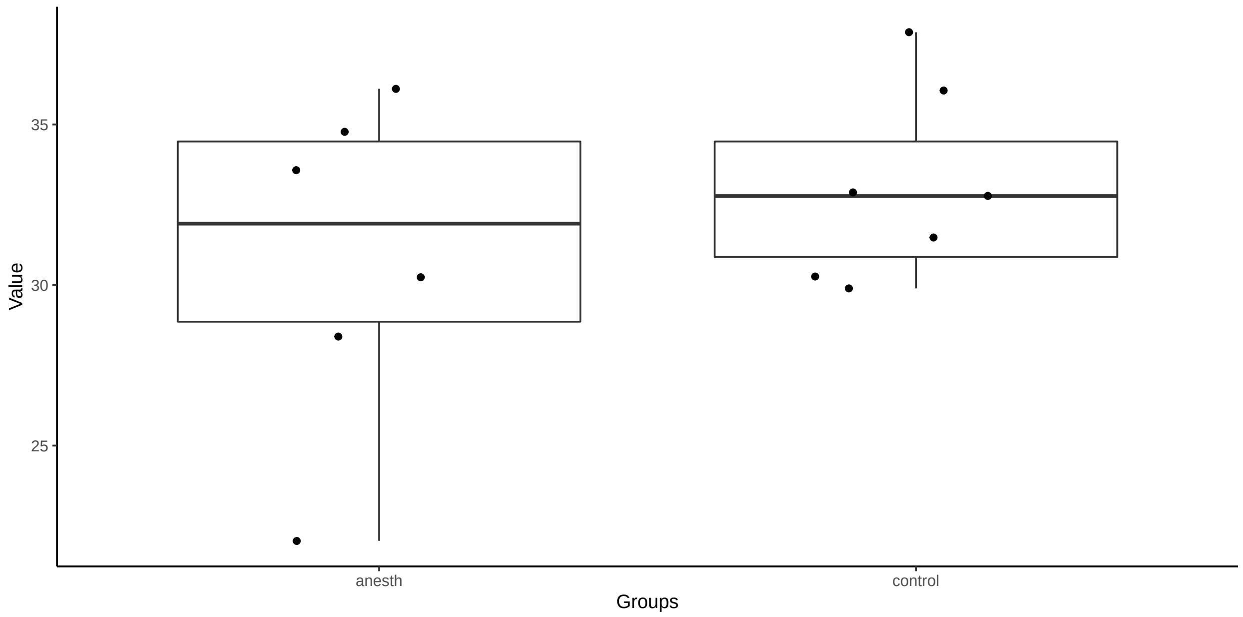


```
## 1. Normality assumption test by Shapiro_Wilk test is
##  p = 0.411
##  Normality assumption was not rejected
## 2. Equal variance test by Bartlett test is
##  p = 0.214
##  Equal variance assumption was not rejected
## 3. The result of anova is
##  p = 0.364
##  A statistically significant difference do not exist between groups
##
```

Data analysis using R

Boohwi Hong

```
## Present data is ** Suppl_Fig5.D_mIPSC_freq_Male.csv **
##
## **  Data structure  **
## 'data.frame':    13 obs. of  4 variables:
##  $ subject: int  1 2 3 4 5 6 7 8 9 10 ...
##  $ group  : chr  "control" "control" "control" "control" ...
##  $ freq   : num  16.45 9.7 9.81 9.55 10.47 ...
##  $ X      : logi  NA NA NA NA NA NA ...
##
## ** Explorative data analysis with graphics**
```

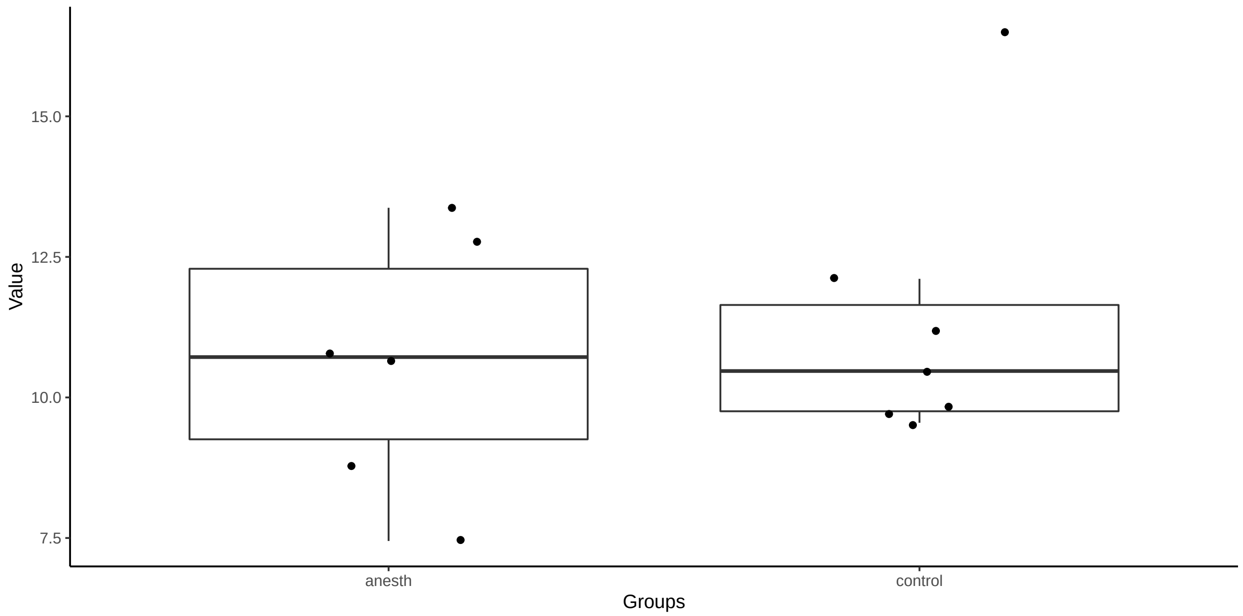


```
## 1. Normality assumption test by Shapiro_Wilk test is
##  p = 0.363
##  Normality assumption was not rejected
## 2. Equal variance test by Bartlett test is
##  p = 0.868
##  Equal variance assumption was not rejected
## 3. The result of anova is
##  p = 0.613
##  A statistically significant difference do not exist between groups
##
```

Supplementary Note 11

The results of statistical analysis for Supplementary Figure 6

Suppl_Fig6_A.LOR_duration_Female Data analysis using R

Boohwi Hong

Package install

Data import

Data structure

```
str(d1)
```

```
## 'data.frame':    60 obs. of  3 variables:
##  $ subject : int  1 2 3 4 5 6 7 8 9 10 ...
##  $ inject  : chr  "1st_inject" "1st_inject" "1st_inject" "1st_inject" ...
##  $ duration: int  273 389 120 200 259 305 136 316 192 227 ...
```

Explorative data analysis with graphics

```
## # A tibble: 5 x 11
##   inject     variable     n   min   max median   iqr  mean    sd    se    ci
##   <chr>      <chr>    <dbl> <dbl> <dbl>  <dbl> <dbl> <dbl> <dbl> <dbl> <dbl>
## 1 1st_inject duration    12     0   613    243  130. 252.  153.   44.2  97.3
## 2 2nd_inject duration    12     0   506      0  259  133.  178.   51.2 113.
## 3 3rd_inject duration    12     0   330      0  316. 107.  158.   45.6 100.
## 4 4th_inject duration    12     0   300      0    0   49.3 115.   33.3  73.2
## 5 5th_inject duration    12     0   272      0    0   22.7  78.5  22.7  49.9
```

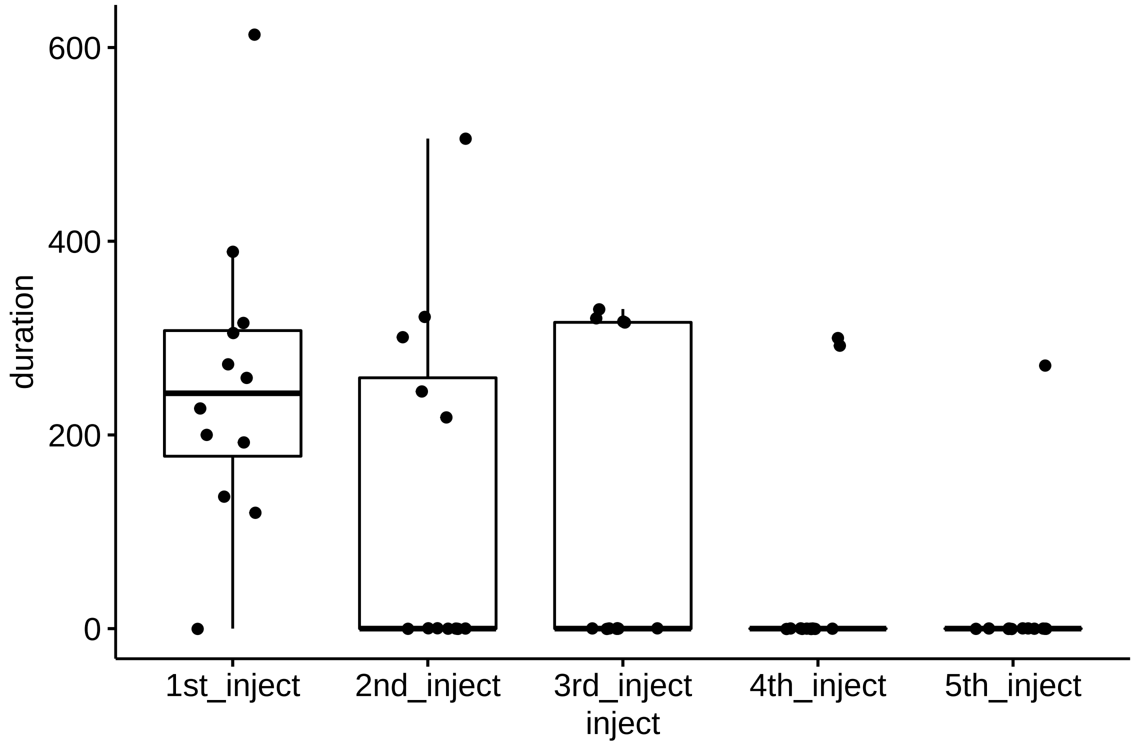

Model fit

```
## # A tibble: 1 x 6
##   .y.          n statistic    df       p method
## * <chr>    <int>     <dbl> <dbl>   <dbl> <chr>
## 1 duration    12      14.6     4 0.00564 Friedman test
```

Effect size

```
## # A tibble: 1 x 5
##   .y.          n effsize method    magnitude
## * <chr>    <int>   <dbl> <chr>     <ord>
## 1 duration    12   0.304 Kendall W moderate
```

Multiple pairwise-comparisons

```
## # A tibble: 10 x 9
##    .y.      group1     group2        n1    n2 statistic     p p.adj p.adj.signif
##  * <chr>    <chr>      <chr>      <int> <int>     <dbl> <dbl> <dbl> <chr>
##  1 duration 1st_inject 2nd_inject    12    12        60 0.11  1     ns
##  2 duration 1st_inject 3rd_inject    12    12        58 0.029 0.294 ns
##  3 duration 1st_inject 4th_inject    12    12        62 0.011 0.113 ns
##  4 duration 1st_inject 5th_inject    12    12        65 0.005 0.051 ns
##  5 duration 2nd_inject 3rd_inject    12    12         6 0.787 1     ns
```

```
##  6 duration 2nd_inject 4th_inject    12    12        13 0.178 1     ns
##  7 duration 2nd_inject 5th_inject    12    12        18 0.142 1     ns
##  8 duration 3rd_inject 4th_inject    12    12        10 0.1   1     ns
##  9 duration 3rd_inject 5th_inject    12    12        14 0.106 1     ns
## 10 duration 4th_inject 5th_inject    12    12         5 0.423 1     ns
```

Interpretation of result

```
## 1. The duration was statistically significantly different at the different time points
##  p = 0.006
```

Suppl_Fig6_B.OFT_diatance_Female Data analysis using R

Boohwi Hong

Package install

Data import

Data structure

```
str(d1)
```

```
## 'data.frame':    120 obs. of  4 variables:
##  $ subject : int  13 14 15 16 17 18 19 20 21 22 ...
##  $ group   : chr  "cont_keta" "cont_keta" "cont_keta" "cont_keta" ...
##  $ trial   : int  1 1 1 1 1 1 1 1 1 1 ...
##  $ distance: num  15.4 21.2 19.2 16.8 19.2 ...
```

Explorative data analysis with graphics

```
##          group trial  n Mean Conf.level Percentile.lower Percentile.upper
## 1  anesth_keta     1 12 16.4       0.95             14.8             17.9
## 2  anesth_keta     2 12 19.3       0.95             16.8             21.7
## 3  anesth_keta     3 12 22.5       0.95             20.3             24.8
## 4  anesth_keta     4 12 21.1       0.95             18.5             23.5
## 5  anesth_keta     5 12 30.0       0.95             27.6             32.5
## 6    cont_keta     1 12 19.7       0.95             17.8             21.7
## 7    cont_keta     2 12 22.2       0.95             20.2             24.3
## 8    cont_keta     3 12 25.4       0.95             21.7             29.2
## 9    cont_keta     4 12 26.7       0.95             23.9             29.6
## 10   cont_keta     5 12 33.1       0.95             29.7             37.0
```

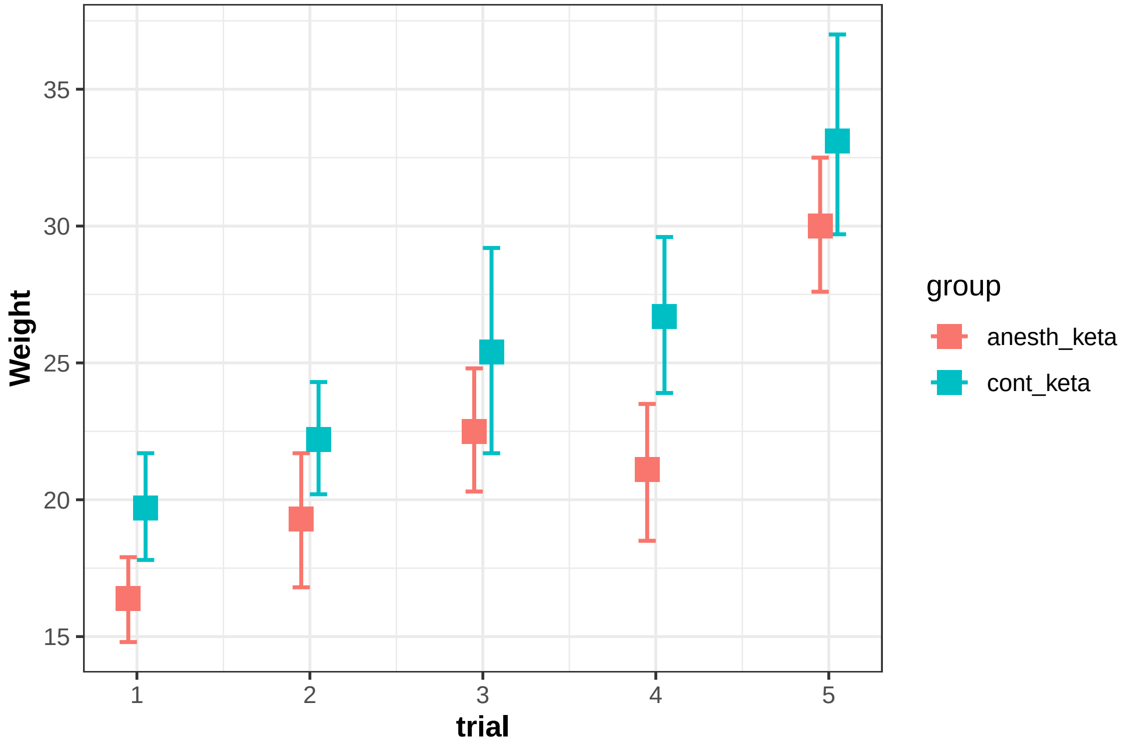

Model fit

```
##
## Error: subject
##       Df Sum Sq Mean Sq
## group  1  306.7   306.7
##
## Error: subject:trial
##       Df Sum Sq Mean Sq
## trial  1   1826    1826
##
## Error: Within
##              Df Sum Sq Mean Sq F value   Pr(>F)
## group         1  103.2   103.2   4.108 0.045017 *
## trial         1  357.3   357.3  14.225 0.000259 ***
## group:trial   1    3.7     3.7   0.149 0.700186
## Residuals   114 2863.3    25.1
## ---
## Signif. codes:  0 '***' 0.001 '**' 0.01 '*' 0.05 '.' 0.1 ' ' 1
```

Interpretation of result

```
## 1. Difference exist between groups
##  p = 0.045

## 2. Difference exist between measurement points
##  p = 0.000

## 3. Significant interaction do not exist between groups and measurement points
##  p = 0.700
```

Supplementary Note 12

The results of statistical analysis
for Supplementary Figure 7

Data analysis using R

Boohwi Hong

Statistical Result

```
## Present data is ** Suppl_Fig7_B.nico-CPP_Anesthesia_Female.csv ** ##
## **  Data structure  **
## 'data.frame':    28 obs. of  3 variables:
## $  ubjectint 1 2 3 4 5 6 7 8 9 10 ...
##  $ group     : chr  "pre" "pre" "pre" "pre" ...
##  $ duration  : num  299 339 226 360 336 ...
##
## ** Explorative data analysis with graphics**
```

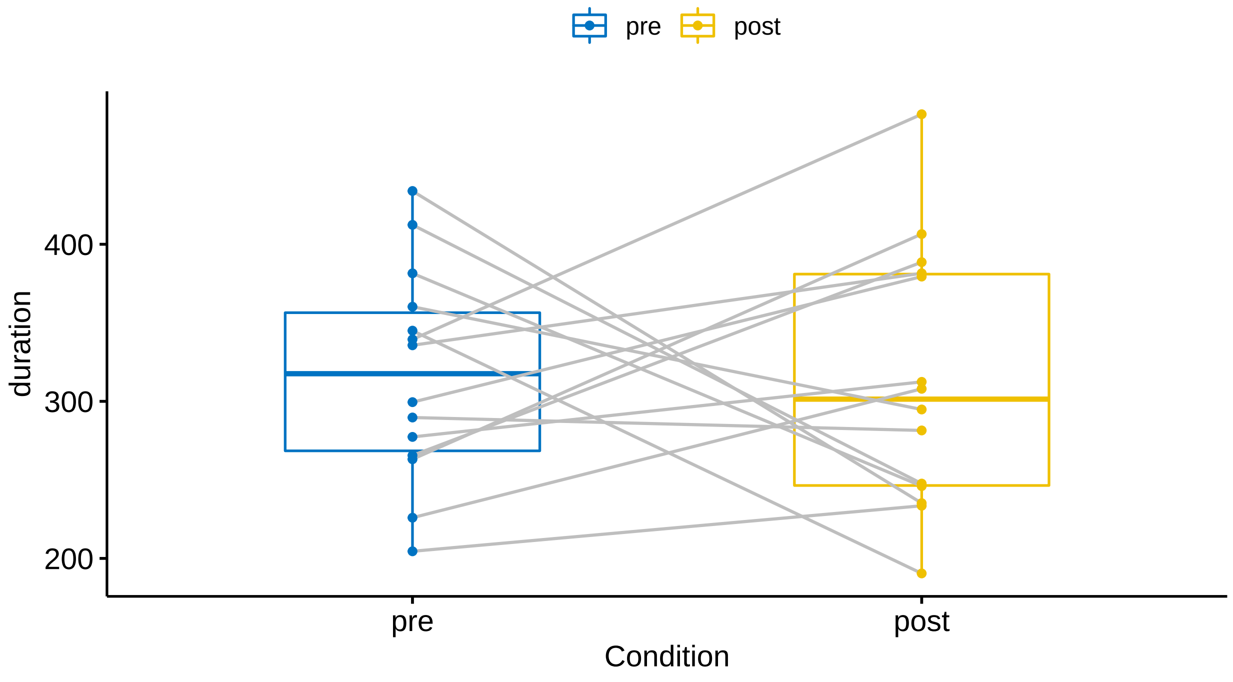


```
## 1. Normality assumption test by Shapiro_Wilk test is
##  p = 0.714
##  Normality assumption was not rejected
## 2. The result of paired t-test is
##  p = 0.921
##  A statistically significant difference do not exist between groups
```

Data analysis using R

Boohwi Hong

Statistical Result

```
## Present data is ** Suppl_Fig7_B.nico-CPP_Control_Female.csv ** ##
## **  Data structure  **
## 'data.frame':    34 obs. of  3 variables:
##$  ubjectint 1 2 3 4 5 6 7 8 9 10 ...
##  $ group     : chr  "pre" "pre" "pre" "pre" ...
##  $ duration  : num  411 425 397 389 427 ...
##
## ** Explorative data analysis with graphics**
```

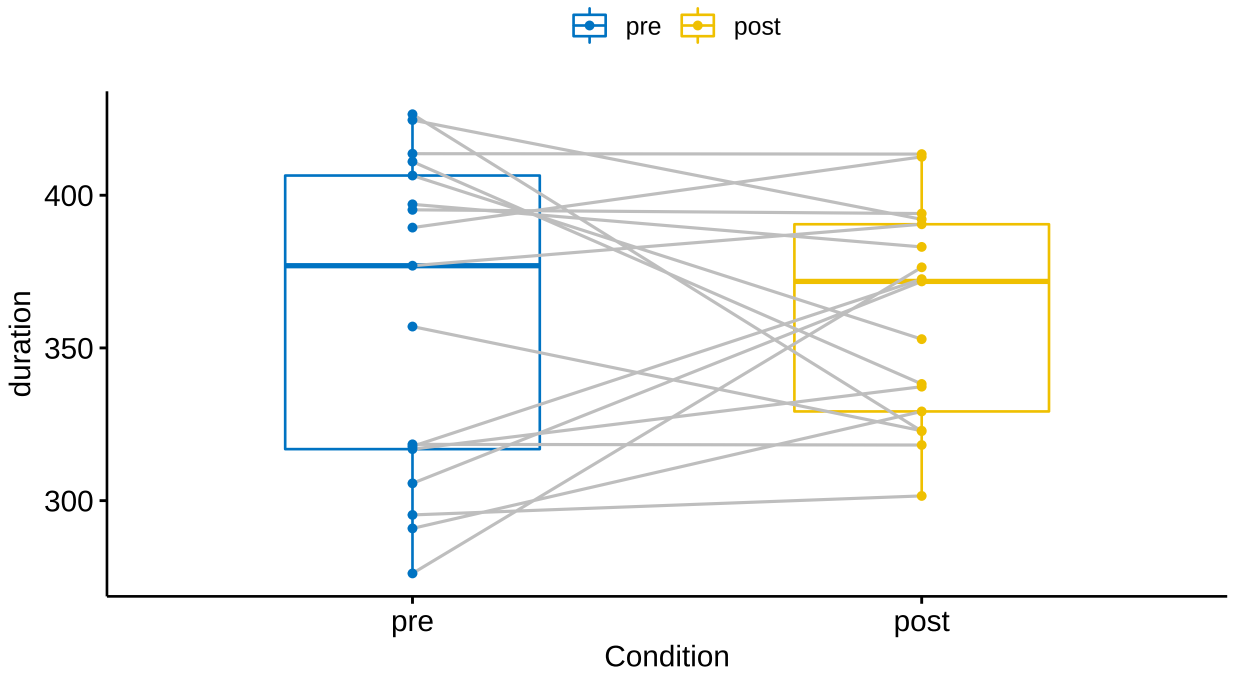


```
## 1. Normality assumption test by Shapiro_Wilk test is
##  p = 0.049
##  Normality assumption was rejected
## 2. The result of Wilcoxon test is
##  p = 0.946
##  A statistically significant difference do not exist between groups
```

Data analysis using R

Boohwi Hong

```
## Present data is ** Suppl_Fig7_C.nicotine-CPP_CPPscore_Female.csv ** ##
## **  Data structure  **

## 'data.frame':    31 obs. of  3 variables:
##  $  ubject: int  1 2 3 4 5 6 7 8 9 10 ...
##  $ group     : chr  "control" "control" "control" "control" ...
##  $ difference: num  -72.8 -32.5 -13.9 23.2 -103.8 ...
##
## ** Explorative data analysis with graphics**
```

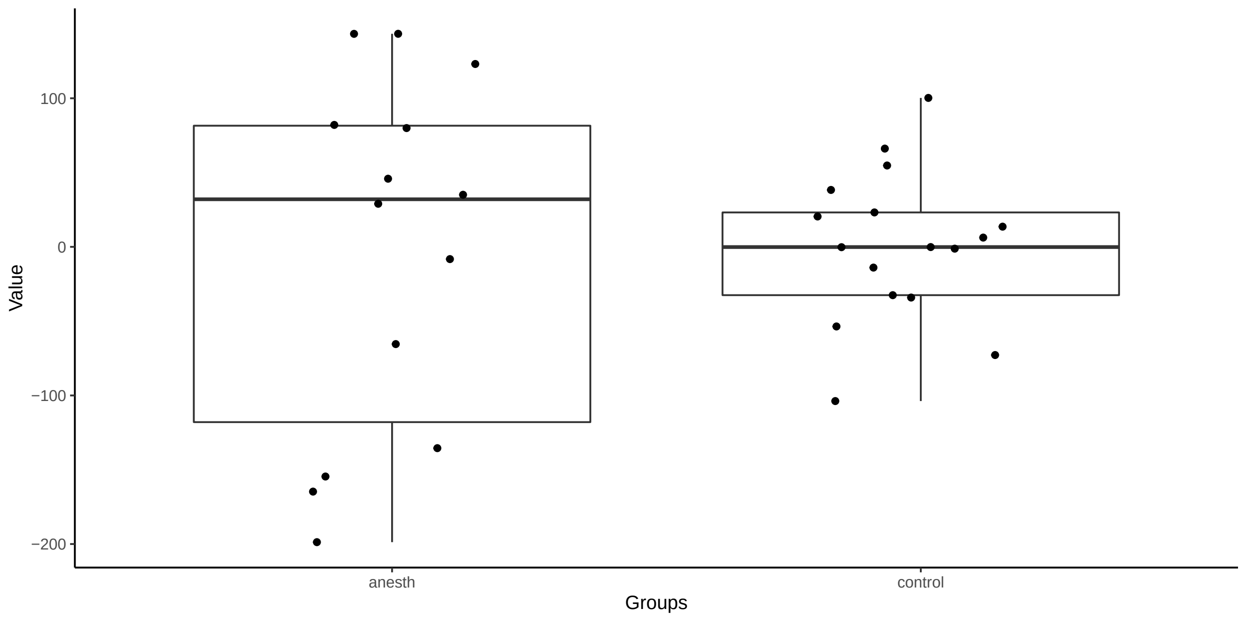


```
## 1. Normality assumption test by Shapiro_Wilk test is
##  p = 0.468
##  Normality assumption was not rejected
## 2. Equal variance test by Bartlett test is
##  p = 0.002
##  Equal variance assumption was rejected
## 3. The result of Welch ANOVA is
##  p = 0.912
##  A statistically significant difference do not exist between groups
##
```

Supplementary Note 13

The results of statistical analysis for Supplementary Figure 8

Suppl_Fig8A.Barnes maze_Total latency Data analysis using R

Boohwi Hong

Package install

Data import

Data structure

```
str(d1)
```

```
## 'data.frame':    92 obs. of  4 variables:
##  $ subject: int  1 2 3 4 5 6 7 8 9 10 ...
##  $ group  : chr  "Control" "Control" "Control" "Control" ...
##  $ trial  : chr  "day1" "day1" "day1" "day1" ...
##  $ value  : num  149.3 83.3 161.3 162 37.7 ...
```

Explorative data analysis with graphics

```
##        group trial  n  Mean Conf.level Percentile.lower Percentile.upper
## 1 Anesthesia  day1 12 100.0       0.95             80.0            120.0
## 2 Anesthesia  day2 12  71.9       0.95             50.3             95.1
## 3 Anesthesia  day3 12  44.8       0.95             37.6             52.6
## 4 Anesthesia  day4 12  27.3       0.95             21.9             33.6
## 5    Control  day1 11 108.0       0.95             81.5            134.0
## 6    Control  day2 11  70.8       0.95             51.8             92.5
## 7    Control  day3 11  39.9       0.95             34.9             45.3
## 8    Control  day4 11  27.5       0.95             18.9             39.4
```

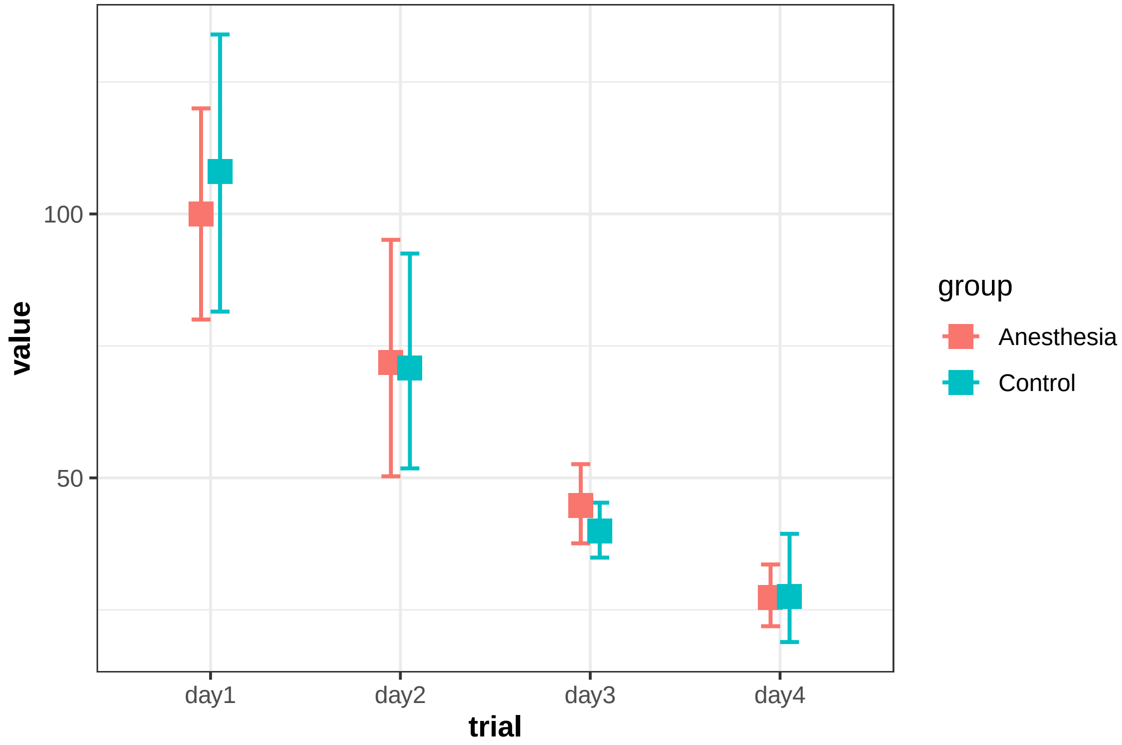

Model fit

```
##
## Error: subject
##       Df Sum Sq Mean Sq
## group  1  5.558   5.558
##
## Error: subject:trial
##       Df Sum Sq Mean Sq
## trial  3  53089   17696
##
## Error: Within
##             Df Sum Sq Mean Sq F value   Pr(>F)
## group        1     70      70   0.080    0.778
## trial        3  28431    9477  10.855 4.66e-06 ***
## group:trial  3   3714    1238   1.418    0.244
## Residuals   80  69843     873
## ---
## Signif. codes:  0 '***' 0.001 '**' 0.01 '*' 0.05 '.' 0.1 ' ' 1
```

Interpretation of result

```
## 1. Difference do not exist between groups
##  p = 0.778

## 2. Difference exist between measurement points
##  p = 0.000

## 3. Significant interaction do not exist between groups and measurement points
##  p = 0.244
```

Suppl_Fig8B.Barnes maze_Total length Data analysis using R

Boohwi Hong

Package install

Data import

Data structure

```
str(d1)
```

```
## 'data.frame':    92 obs. of  4 variables:
##  $ subject: int  1 2 3 4 5 6 7 8 9 10 ...
##  $ group  : chr  "Control" "Control" "Control" "Control" ...
##  $ trial  : chr  "day1" "day1" "day1" "day1" ...
##  $ value  : num  860 603 1190 958 1480 ...
```

Explorative data analysis with graphics

```
##        group trial  n Mean Conf.level Percentile.lower Percentile.upper
## 1 Anesthesia  day1 12  554       0.95              429              669
## 2 Anesthesia  day2 12  471       0.95              354              600
## 3 Anesthesia  day3 12  307       0.95              234              382
## 4 Anesthesia  day4 12  221       0.95              179              270
## 5    Control  day1 11  806       0.95              612             1020
## 6    Control  day2 11  511       0.95              381              652
## 7    Control  day3 11  316       0.95              270              372
## 8    Control  day4 11  224       0.95              152              326
```

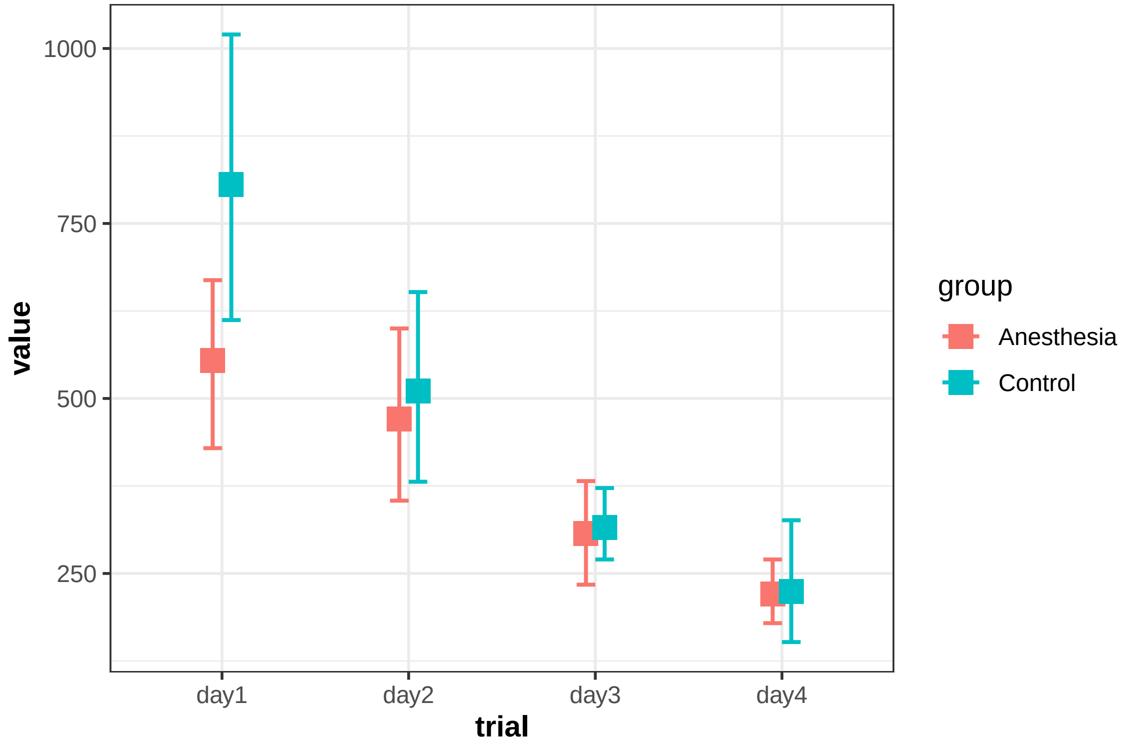

Model fit

```
##
## Error: subject
##       Df Sum Sq Mean Sq
## group  1  64127   64127
##
## Error: subject:trial
##       Df  Sum Sq Mean Sq
## trial  3 1525768  508589
##
## Error: Within
##             Df  Sum Sq Mean Sq F value   Pr(>F)
## group        1   83986   83986   1.964    0.165
## trial        3 1626452  542151  12.675 7.36e-07 ***
## group:trial  3   56882   18961   0.443    0.723
## Residuals   80 3421809   42773
## ---
## Signif. codes:  0 '***' 0.001 '**' 0.01 '*' 0.05 '.' 0.1 ' ' 1
```

Interpretation of result

```
## 1. Difference do not exist between groups
##  p = 0.165

## 2. Difference exist between measurement points
##  p = 0.000

## 3. Significant interaction do not exist between groups and measurement points
##  p = 0.723
```

Suppl_Fig8C.Barnes maze_Total errors Data analysis using R

Boohwi Hong

Package install

Data import

Data structure

```
str(d1)
```

```
## 'data.frame':    92 obs. of  4 variables:
##  $ subject: int  1 2 3 4 5 6 7 8 9 10 ...
##  $ group  : chr  "Control" "Control" "Control" "Control" ...
##  $ trial  : chr  "day1" "day1" "day1" "day1" ...
##  $ value  : num  9.67 10.33 10.67 20 17 ...
```

Explorative data analysis with graphics

```
##        group trial  n  Mean Conf.level Percentile.lower Percentile.upper
## 1 Anesthesia  day1 12 11.10       0.95             8.78            13.10
## 2 Anesthesia  day2 12 10.20       0.95             7.36            13.10
## 3 Anesthesia  day3 12  9.44       0.95             7.58            11.40
## 4 Anesthesia  day4 12  7.33       0.95             6.17             8.89
## 5    Control  day1 11 12.80       0.95            10.00            15.60
## 6    Control  day2 11 11.90       0.95             8.43            16.20
## 7    Control  day3 11  8.45       0.95             6.97             9.94
## 8    Control  day4 11  7.09       0.95             4.73             9.70
```

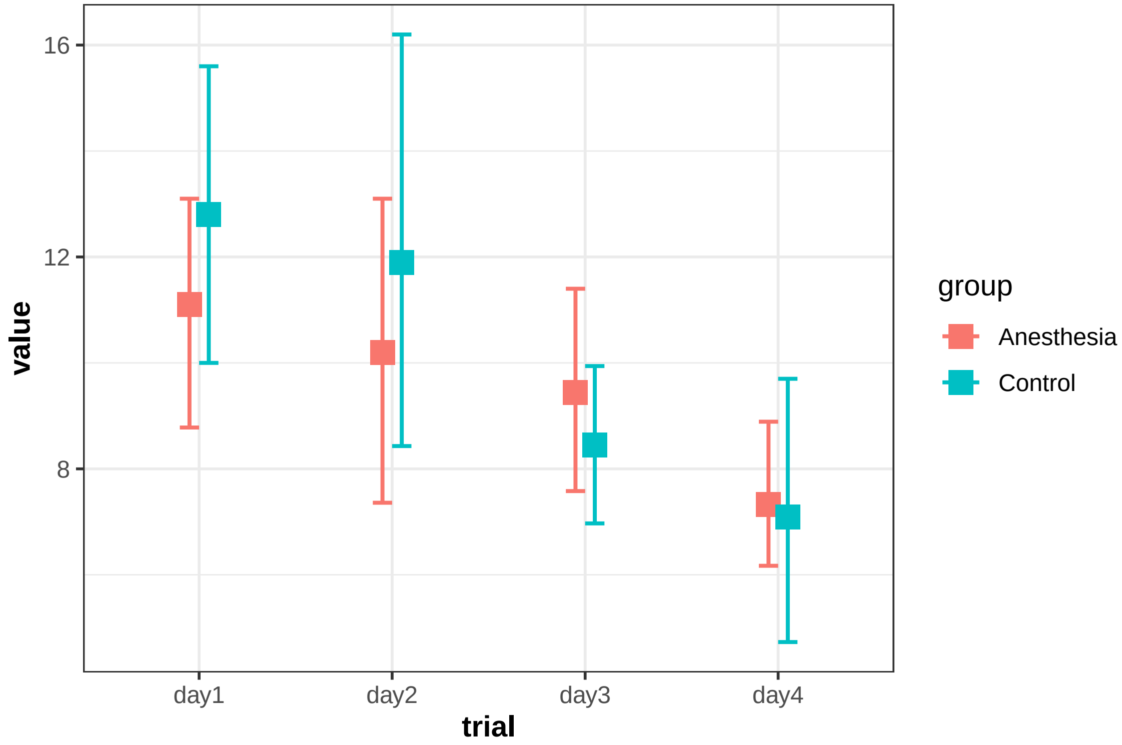

Model fit

```
##
## Error: subject
##       Df Sum Sq Mean Sq
## group  1  3.758   3.758
##
## Error: subject:trial
##       Df Sum Sq Mean Sq
## trial  3  166.2    55.4
##
## Error: Within
##             Df Sum Sq Mean Sq F value Pr(>F)
## group        1   76.3   76.31   3.882 0.0523 .
## trial        3  178.5   59.48   3.026 0.0343 *
## group:trial  3   53.4   17.81   0.906 0.4422
## Residuals   80 1572.8   19.66
## ---
## Signif. codes:  0 '***' 0.001 '**' 0.01 '*' 0.05 '.' 0.1 ' ' 1
```

Interpretation of result

```
## 1. Difference do not exist between groups
##  p = 0.052

## 2. Difference exist between measurement points
##  p = 0.034

## 3. Significant interaction do not exist between groups and measurement points
##  p = 0.442
```

Suppl_Fig8D.Barnes maze_Primary latency Data analysis using R

Boohwi Hong

Package install

Data import

Data structure

```
str(d1)
```

```
## 'data.frame':    92 obs. of  4 variables:
##  $ subject: int  1 2 3 4 5 6 7 8 9 10 ...
##  $ group  : chr  "Control" "Control" "Control" "Control" ...
##  $ trial  : chr  "day1" "day1" "day1" "day1" ...
##  $ value  : num  45.3 22.1 135 126.5 27.1 ...
```

Explorative data analysis with graphics

```
##        group trial  n Mean Conf.level Percentile.lower Percentile.upper
## 1 Anesthesia  day1 12 74.8       0.95             53.0             97.2
## 2 Anesthesia  day2 12 58.2       0.95             37.9             80.9
## 3 Anesthesia  day3 12 31.2       0.95             23.7             39.7
## 4 Anesthesia  day4 12 20.8       0.95             15.5             27.1
## 5    Control  day1 11 79.7       0.95             53.2            109.0
## 6    Control  day2 11 58.9       0.95             42.0             79.1
## 7    Control  day3 11 27.6       0.95             21.3             34.3
## 8    Control  day4 11 21.2       0.95             13.8             32.7
```

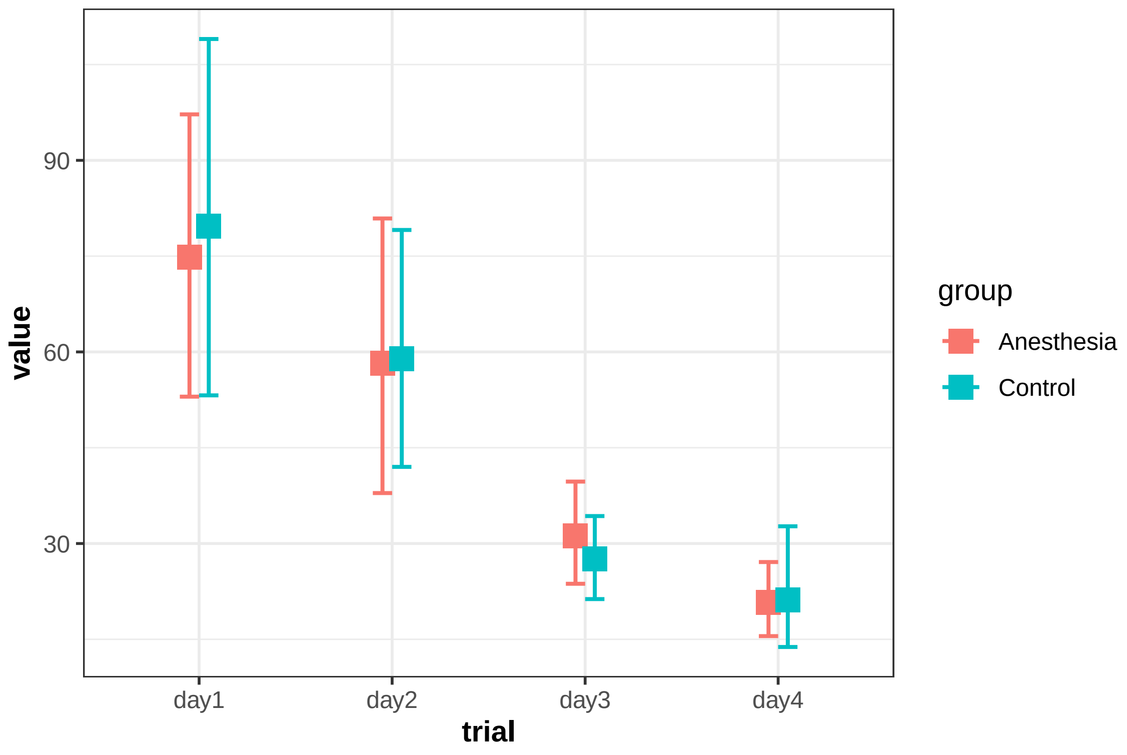

Model fit

```
##
## Error: subject
##       Df Sum Sq Mean Sq
## group  1    285     285
##
## Error: subject:trial
##       Df Sum Sq Mean Sq
## trial  3  34977   11659
##
## Error: Within
##             Df Sum Sq Mean Sq F value  Pr(>F)
## group        1   1227    1227   1.291 0.25926
## trial        3  11870    3957   4.164 0.00856 **
## group:trial  3    552     184   0.194 0.90049
## Residuals   80  76012     950
## ---
## Signif. codes:  0 '***' 0.001 '**' 0.01 '*' 0.05 '.' 0.1 ' ' 1
```

Interpretation of result

```
## 1. Difference do not exist between groups
##  p = 0.259

## 2. Difference exist between measurement points
##  p = 0.009

## 3. Significant interaction do not exist between groups and measurement points
##  p = 0.900
```

Suppl_Fig8E.Barnes maze_Primary length Data analysis using R

Boohwi Hong

Package install

Data import

Data structure

```
str(d1)
```

```
## 'data.frame':    92 obs. of  4 variables:
##  $ subject: int  1 2 3 4 5 6 7 8 9 10 ...
##  $ group  : chr  "Control" "Control" "Control" "Control" ...
##  $ trial  : chr  "day1" "day1" "day1" "day1" ...
##  $ value  : num  172 230 691 780 169 ...
```

Explorative data analysis with graphics

```
##        group trial  n Mean Conf.level Percentile.lower Percentile.upper
## 1 Anesthesia  day1 12  435       0.95              299              583
## 2 Anesthesia  day2 12  427       0.95              305              559
## 3 Anesthesia  day3 12  252       0.95              182              333
## 4 Anesthesia  day4 12  206       0.95              167              253
## 5    Control  day1 11  502       0.95              339              675
## 6    Control  day2 11  470       0.95              350              606
## 7    Control  day3 11  253       0.95              193              312
## 8    Control  day4 11  207       0.95              140              305
```

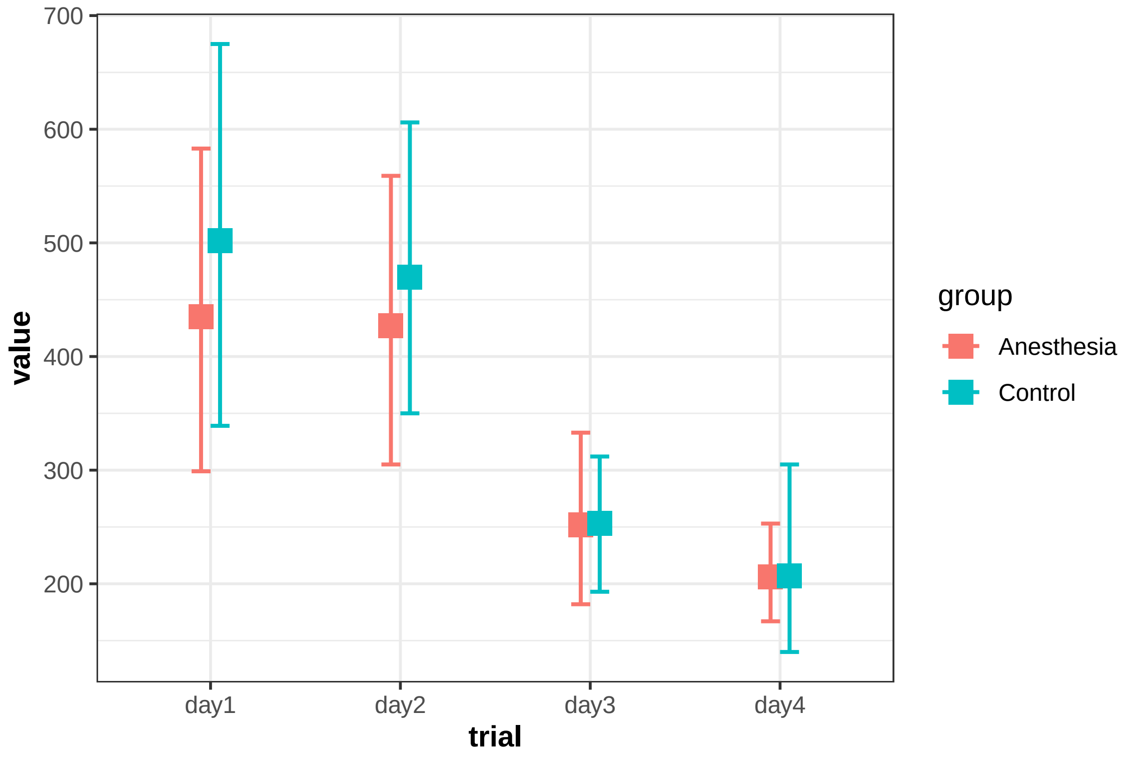

Model fit

```
##
## Error: subject
##       Df Sum Sq Mean Sq
## group  1   4016    4016
##
## Error: subject:trial
##       Df Sum Sq Mean Sq
## trial  3 911233  303744
##
## Error: Within
##             Df  Sum Sq Mean Sq F value Pr(>F)
## group        1  144686  144686   3.565 0.0626 .
## trial        3  309888  103296   2.545 0.0619 .
## group:trial  3   51635   17212   0.424 0.7363
## Residuals   80 3247144   40589
## ---
## Signif. codes:  0 '***' 0.001 '**' 0.01 '*' 0.05 '.' 0.1 ' ' 1
```

Interpretation of result

```
## 1. Difference do not exist between groups
##  p = 0.063

## 2. Difference do not exist between measurement points
##  p = 0.062

## 3. Significant interaction do not exist between groups and measurement points
##  p = 0.736
```

Suppl_Fig8F.Barnes maze_Primary errors Data analysis using R

Boohwi Hong

Package install

Data import

Data structure

```
str(d1)
```

```
## 'data.frame':    92 obs. of  4 variables:
##  $ subject: int  1 2 3 4 5 6 7 8 9 10 ...
##  $ group  : chr  "Control" "Control" "Control" "Control" ...
##  $ trial  : chr  "day1" "day1" "day1" "day1" ...
##  $ value  : num  3.33 3.67 7 15.33 4.67 ...
```

Explorative data analysis with graphics

```
##        group trial  n Mean Conf.level Percentile.lower Percentile.upper
## 1 Anesthesia  day1 12 8.89       0.95             6.36            11.50
## 2 Anesthesia  day2 12 9.08       0.95             6.50            11.70
## 3 Anesthesia  day3 12 7.94       0.95             6.19             9.89
## 4 Anesthesia  day4 12 7.14       0.95             5.89             8.83
## 5    Control  day1 11 9.06       0.95             5.91            12.60
## 6    Control  day2 11 9.94       0.95             6.97            14.10
## 7    Control  day3 11 6.70       0.95             5.06             8.36
## 8    Control  day4 11 6.58       0.95             4.52             8.97
```

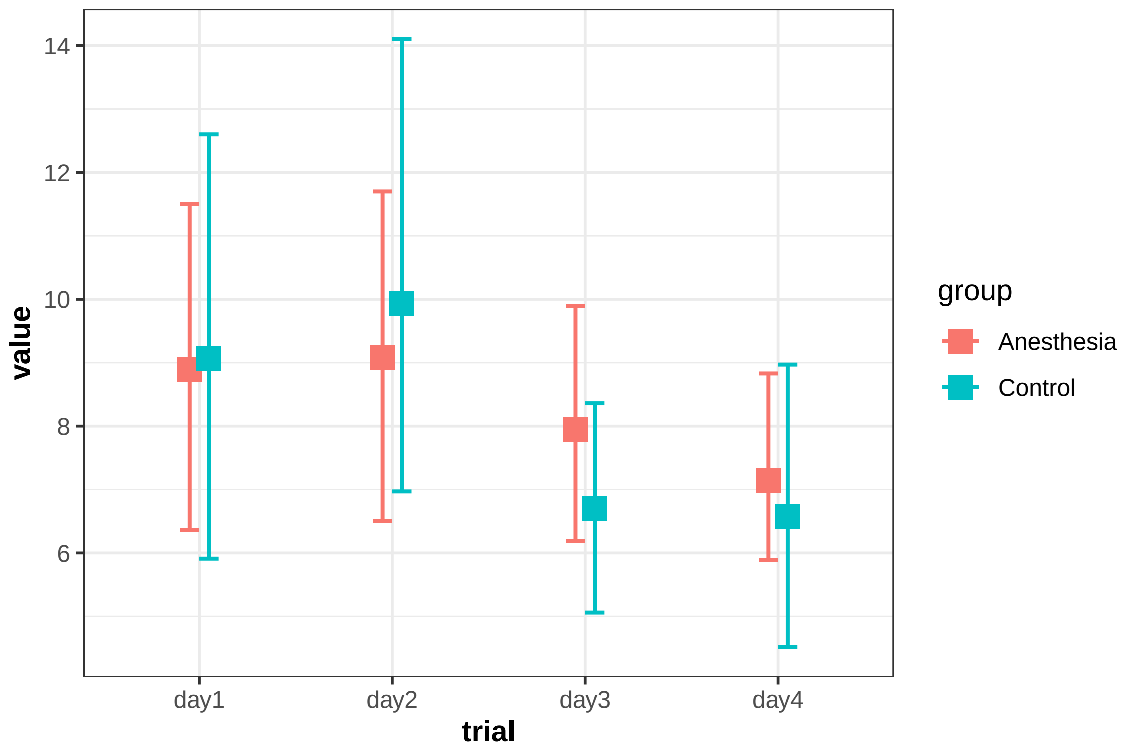

Model fit

```
##
## Error: subject
##       Df Sum Sq Mean Sq
## group  1  33.21   33.21
##
## Error: subject:trial
##       Df Sum Sq Mean Sq
## trial  3  62.01   20.67
##
## Error: Within
##             Df Sum Sq Mean Sq F value Pr(>F)
## group        1   65.7   65.72   3.326 0.0719 .
## trial        3   60.8   20.27   1.026 0.3857
## group:trial  3   11.3    3.77   0.191 0.9022
## Residuals   80 1580.8   19.76
## ---
## Signif. codes:  0 '***' 0.001 '**' 0.01 '*' 0.05 '.' 0.1 ' ' 1
```

Interpretation of result

```
## 1. Difference do not exist between groups
##  p = 0.072

## 2. Difference do not exist between measurement points
##  p = 0.386

## 3. Significant interaction do not exist between groups and measurement points
##  p = 0.902
```

Suppl_Fig8G.Barnes maze_distance Data analysis using R

Boohwi Hong

Package install

Data import

Data structure

```
str(d1)
```

```
## 'data.frame':    23 obs. of  3 variables:
##  $ subject: int  1 2 3 4 5 6 7 8 9 10 ...
##  $ group  : chr  "Control" "Control" "Control" "Control" ...
##  $ value  : num  663 792 470 554 543 ...
```

Explorative data analysis with graphics

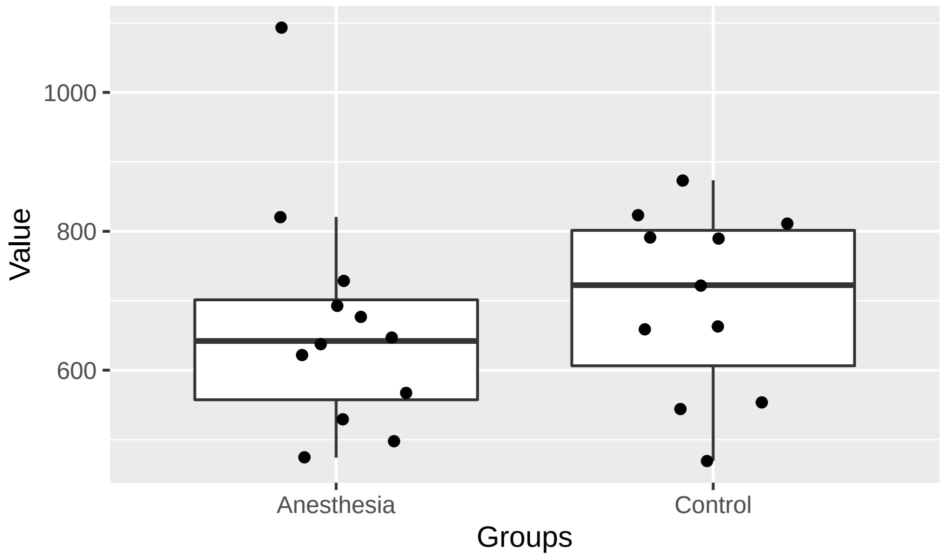

Easystat function developed by S. Park (available at https://rpubs.com/goodlebang)

Statistical Result

```
easystat(d1)
```

```
## 1. Normality assumption test by Shapiro_Wilk test is
##  p = 0.213
##  Normality assumption was not rejected
## 2. Equal variance test by Bartlett test is
##  p = 0.470
##  Equal variance assumption was not rejected
## 3. The result of anova is
##  p = 0.5905
##  A statistically significant difference do not exist between groups
```

Suppl_Fig8G.Barnes maze_latency Data analysis using R

Boohwi Hong

Package install

Data import

Data structure

```
str(d1)
```

```
## 'data.frame':    23 obs. of  3 variables:
##  $ subject: int  1 2 3 4 5 6 7 8 9 10 ...
##  $ group  : chr  "Control" "Control" "Control" "Control" ...
##  $ value  : num  18.4 27.9 12.8 17.7 31.1 ...
```

Explorative data analysis with graphics

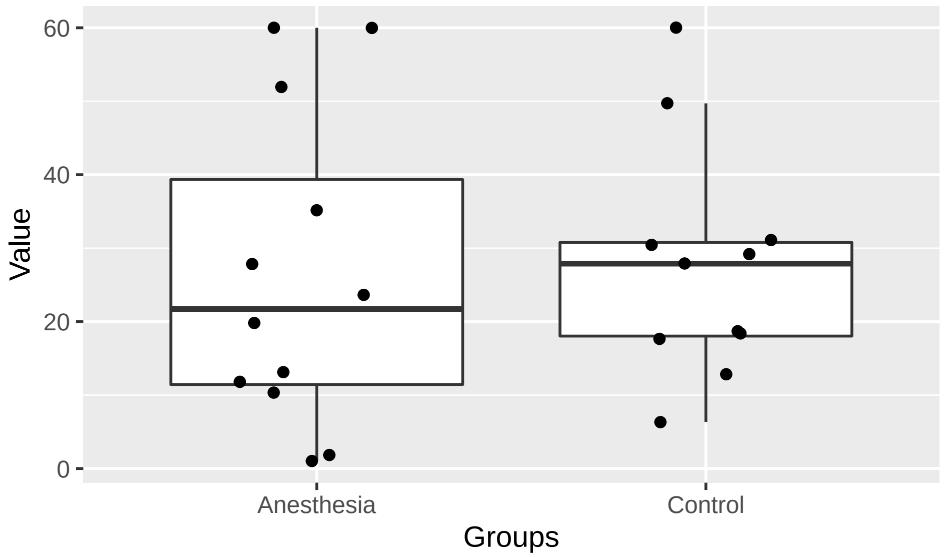

Easystat function developed by S. Park (available at https://rpubs.com/goodlebang)

Statistical Result

```
easystat(d1)
```

```
## 1. Normality assumption test by Shapiro_Wilk test is
##  p = 0.043
##  Normality assumption was rejected
## 2. The result of Kruskall_Wallis test:
##  p = 0.666
##  A statistically significant difference do not exist between groups
```

Suppl_Fig8G.Barnes maze_time Data analysis using R

Boohwi Hong

Package install

Data import

Data structure

```
str(d1)
```

```
## 'data.frame':    23 obs. of  3 variables:
##  $ subject: int  1 2 3 4 5 6 7 8 9 10 ...
##  $ group  : chr  "Control" "Control" "Control" "Control" ...
##  $ value  : num  39.1 35 44.8 24.1 44.4 ...
```

Explorative data analysis with graphics

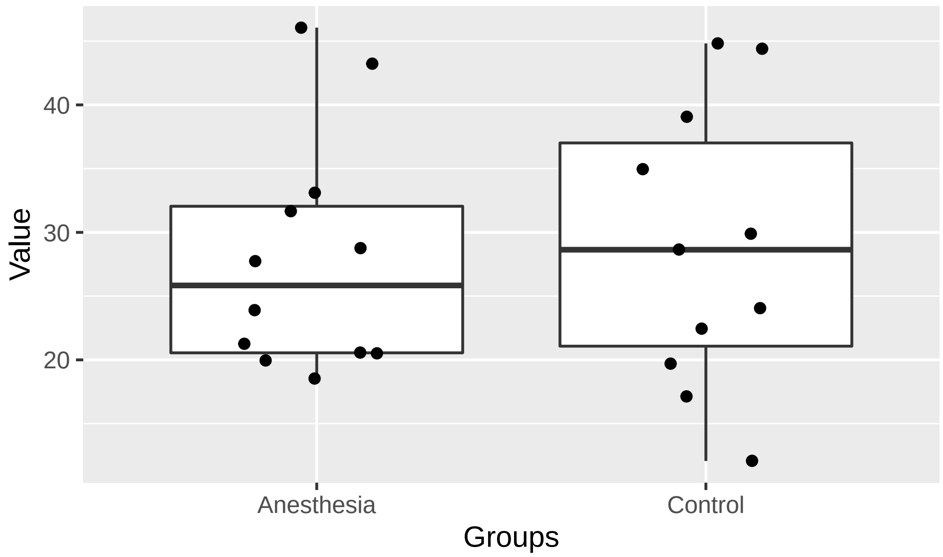

Easystat function developed by S. Park (available at https://rpubs.com/goodlebang)

Statistical Result

```
easystat(d1)
```

```
## 1. Normality assumption test by Shapiro_Wilk test is
##  p = 0.165
##  Normality assumption was not rejected
## 2. Equal variance test by Bartlett test is
##  p = 0.572
##  Equal variance assumption was not rejected
## 3. The result of anova is
##  p = 0.8344
##  A statistically significant difference do not exist between groups
```

Suppl_Fig8H.Fear chamber test Data analysis using R

Boohwi Hong

Package install

Data import

Data structure

```
str(d1)
```

```
## 'data.frame':    23 obs. of  3 variables:
##  $ subject: int  1 2 3 4 5 6 7 8 9 10 ...
##  $ group  : chr  "Control" "Control" "Control" "Control" ...
##  $ value  : num  43.11 72.44 4.09 51.29 40.98 ...
```

Explorative data analysis with graphics

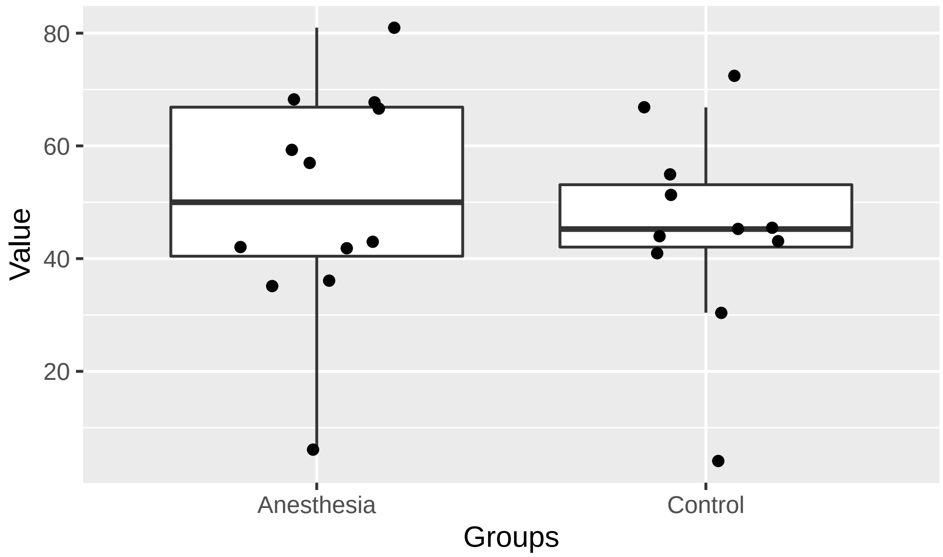

Easystat function developed by S. Park (available at https://rpubs.com/goodlebang)

Statistical Result

```
easystat(d1)
```

```
## 1. Normality assumption test by Shapiro_Wilk test is
##  p = 0.26
##  Normality assumption was not rejected
## 2. Equal variance test by Bartlett test is
##  p = 0.717
##  Equal variance assumption was not rejected
## 3. The result of anova is
##  p = 0.5418
##  A statistically significant difference do not exist between groups
```

Supplementary Note 14

The results of statistical analysis
for Supplementary Figure 9

Data analysis using R

Boohwi Hong

```
## Present data is ** Suppl_Fig9.A_RMP_Female.csv **
##
## **  Data structure  **
## 'data.frame':    12 obs. of  3 variables:
##  $  ubject: int  1 2 3 4 5 6 7 8 9 10 ...
##  $ group     : chr  "control" "control" "control" "control" ...
##  $ RMP       : num  -60 -60.5 -58 -59.4 -58.8 ...
##
## ** Explorative data analysis with graphics**
```

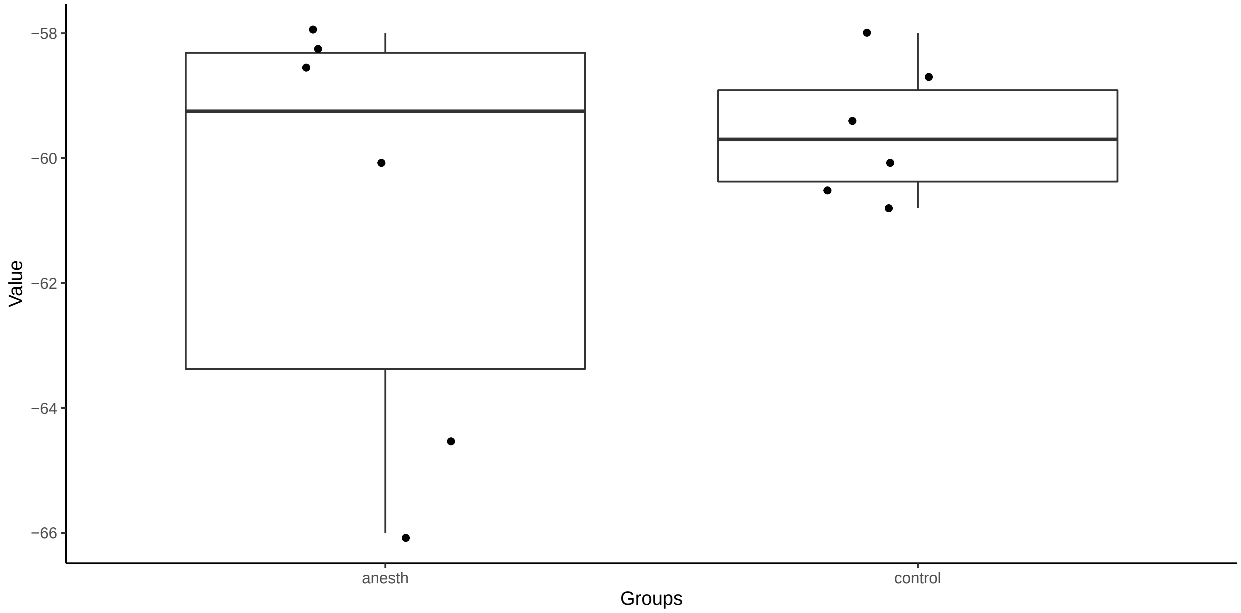


```
## 1. Normality assumption test by Shapiro_Wilk test is
##  p = 0.298
##  Normality assumption was not rejected
## 2. Equal variance test by Bartlett test is
##  p = 0.022
##  Equal variance assumption was rejected
## 3. The result of Welch ANOVA is
##  p = 0.417
##  A statistically significant difference do not exist between groups
##
```

Data analysis using R

Boohwi Hong

```
## Present data is ** Suppl_Fig9.B_Threshold_Female.csv **
##
## **  Data structure  **
## 'data.frame':    12 obs. of  3 variables:
##  $ subject  : int  1 2 3 4 5 6 7 8 9 10 ...
##  $ group    : chr  "control" "control" "control" "control" ...
##  $ Threshold: num  -39.5 -40.1 -36 -45.3 -48.8 ...
##
## ** Explorative data analysis with graphics**
```

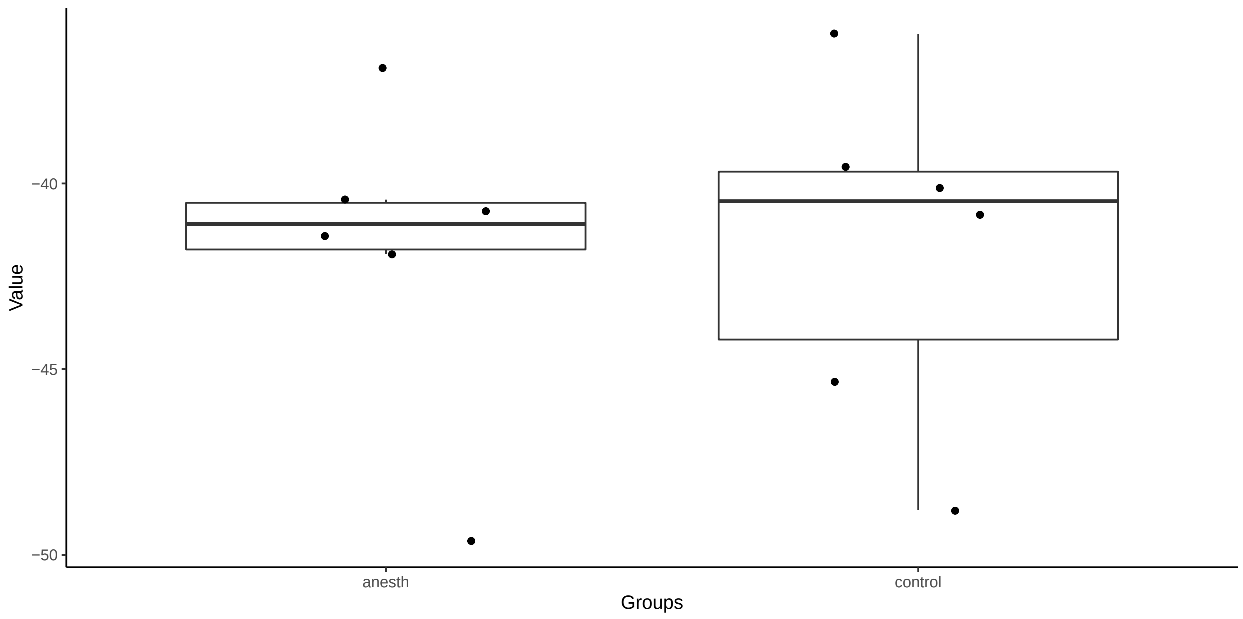


```
## 1. Normality assumption test by Shapiro_Wilk test is
##  p = 0.141
##  Normality assumption was not rejected
## 2. Equal variance test by Bartlett test is
##  p = 0.864
##  Equal variance assumption was not rejected
## 3. The result of anova is
##  p = 0.975
##  A statistically significant difference do not exist between groups
##
```

Data analysis using R

Boohwi Hong

```
## Present data is ** Suppl_Fig9.C_Input Res_Female.csv **
##
## **  Data structure  **
## 'data.frame':    12 obs. of  3 variables:
##  $ subject  : int  1 2 3 4 5 6 7 8 9 10 ...
##  $ group    : chr  "control" "control" "control" "control" ...
##  $ Input.Res: num  183 190 202 270 338 ...
##
## ** Explorative data analysis with graphics**
```

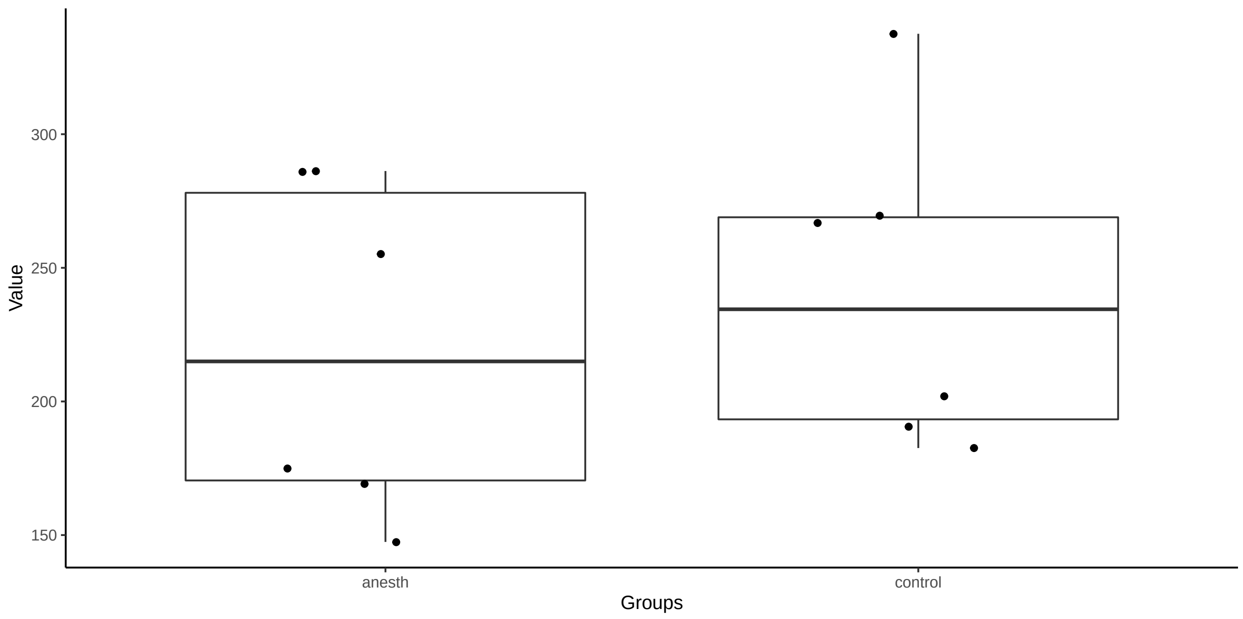


```
## 1. Normality assumption test by Shapiro_Wilk test is
##  p = 0.102
##  Normality assumption was not rejected
## 2. Equal variance test by Bartlett test is
##  p = 0.931
##  Equal variance assumption was not rejected
## 3. The result of anova is
##  p = 0.555
##  A statistically significant difference do not exist between groups
##
```

Supplementary Note 15

The results of statistical analysis
for Supplementary Figure 10

Data analysis using R

Boohwi Hong

```
## Present data is ** Suppl_Fig10.B_mEPSC_amp_female.csv **
##
## **  Data structure  **
## 'data.frame':    15 obs. of  3 variables:
##  $ subject: int  1 2 3 4 5 6 7 8 9 10 ...
##  $ group  : chr  "control" "control" "control" "control" ...
##  $ amp    : num  37.1 29.9 17.5 22.4 39.4 ...
##
## ** Explorative data analysis with graphics**
```

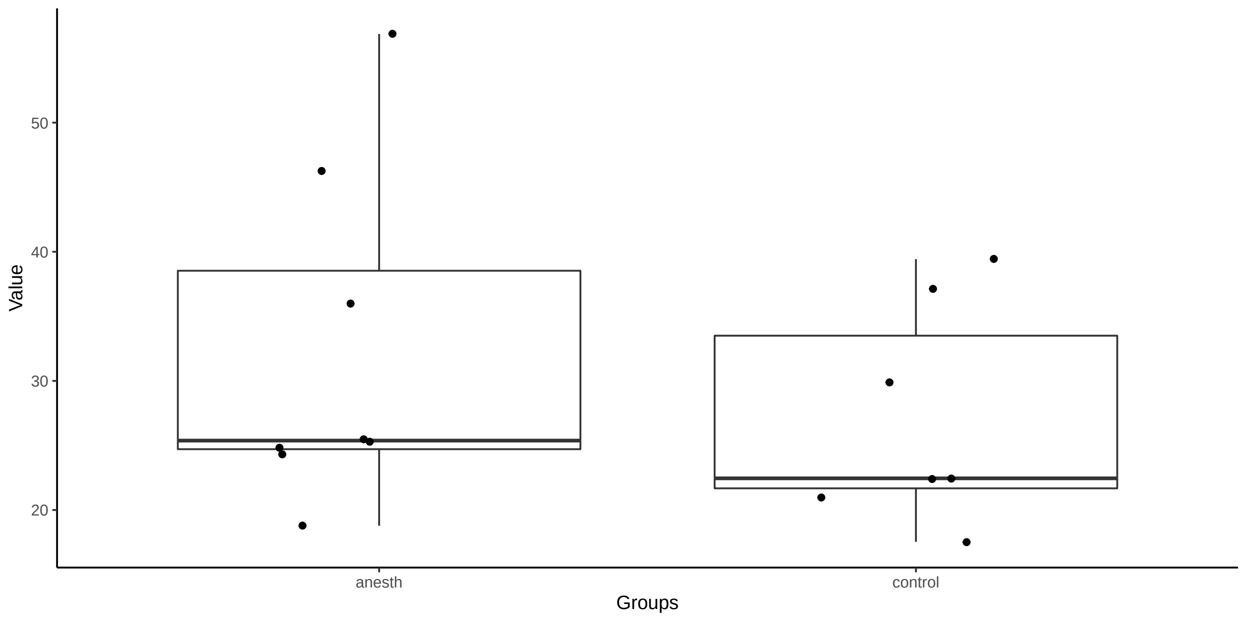


```
## 1. Normality assumption test by Shapiro_Wilk test is
##  p = 0.052
##  Normality assumption was not rejected
## 2. Equal variance test by Bartlett test is
##  p = 0.298
##  Equal variance assumption was not rejected
## 3. The result of anova is
##  p = 0.396
##  A statistically significant difference do not exist between groups
##
```

Data analysis using R

Boohwi Hong

```
## Present data is ** Suppl_Fig10.B_mEPSC_freq_female.csv **
##
## **  Data structure  **
## 'data.frame':    15 obs. of  3 variables:
##  $ subject: int  1 2 3 4 5 6 7 8 9 10 ...
##  $ group  : chr  "control" "control" "control" "control" ...
##  $ freq   : num  0.00417 0.00833 0.025 0.04167 0.04167 ...
##
## ** Explorative data analysis with graphics**
```

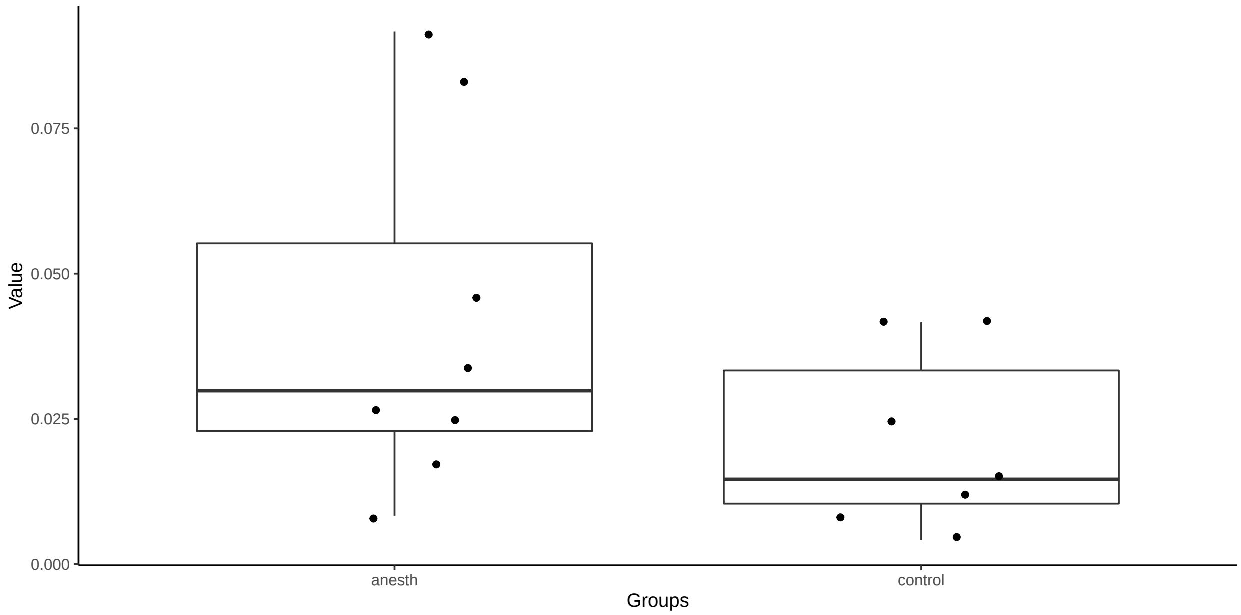


```
## 1. Normality assumption test by Shapiro_Wilk test is
##  p = 0.149
##  Normality assumption was not rejected
## 2. Equal variance test by Bartlett test is
##  p = 0.111
##  Equal variance assumption was not rejected
## 3. The result of anova is
##  p = 0.140
##  A statistically significant difference do not exist between groups
##
```

Data analysis using R

Boohwi Hong

```
## Present data is ** Suppl_Fig10.D_mIPSC_amp_female.csv **
##
## **  Data structure  **
## 'data.frame':    14 obs. of  3 variables:
##  $ subject: int  1 2 3 4 5 6 7 8 9 10 ...
##  $ group  : chr  "control" "control" "control" "control" ...
##  $ amp    : num  38.6 27.2 34 27.4 41.2 ...
##
## ** Explorative data analysis with graphics**
```

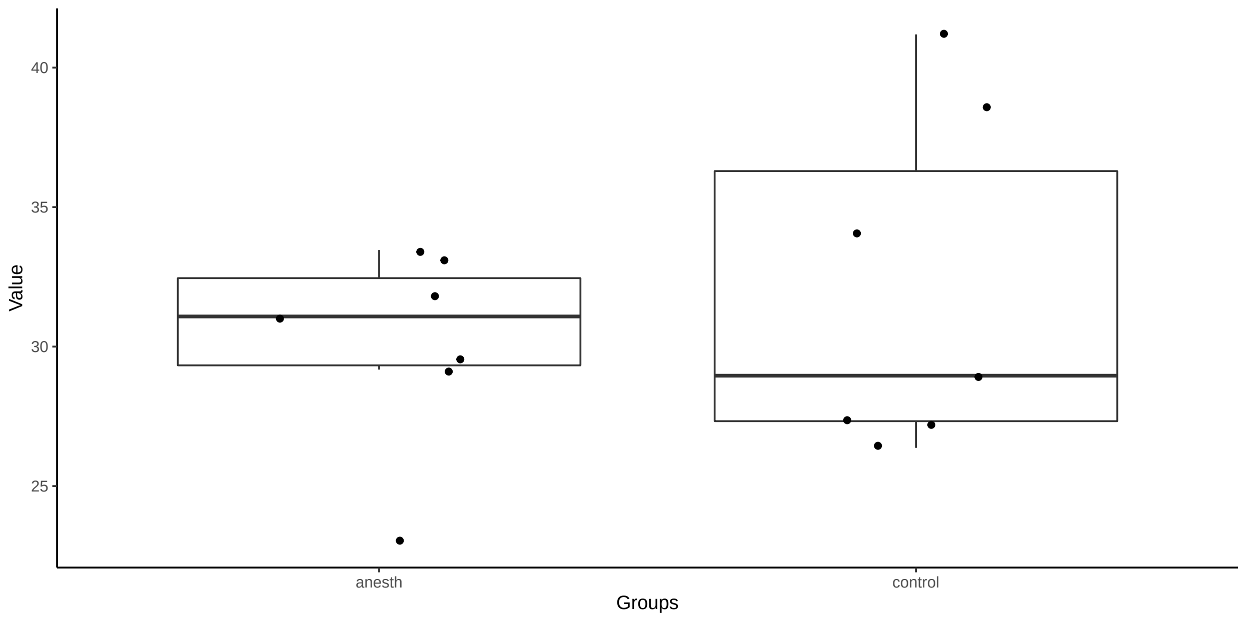


```
## 1. Normality assumption test by Shapiro_Wilk test is
##  p = 0.874
##  Normality assumption was not rejected
## 2. Equal variance test by Bartlett test is
##  p = 0.227
##  Equal variance assumption was not rejected
## 3. The result of anova is
##  p = 0.506
##  A statistically significant difference do not exist between groups
##
```

Data analysis using R

Boohwi Hong

```
## Present data is ** Suppl_Fig10.D_mIPSC_freq_female.csv **
##
## **  Data structure  **
## 'data.frame':    14 obs. of  3 variables:
##  $ subject: int  1 2 3 4 5 6 7 8 9 10 ...
##  $ group  : chr  "control" "control" "control" "control" ...
##  $ freq   : num  12.7 12 14.4 11.2 13.6 ...
##
## ** Explorative data analysis with graphics**
```

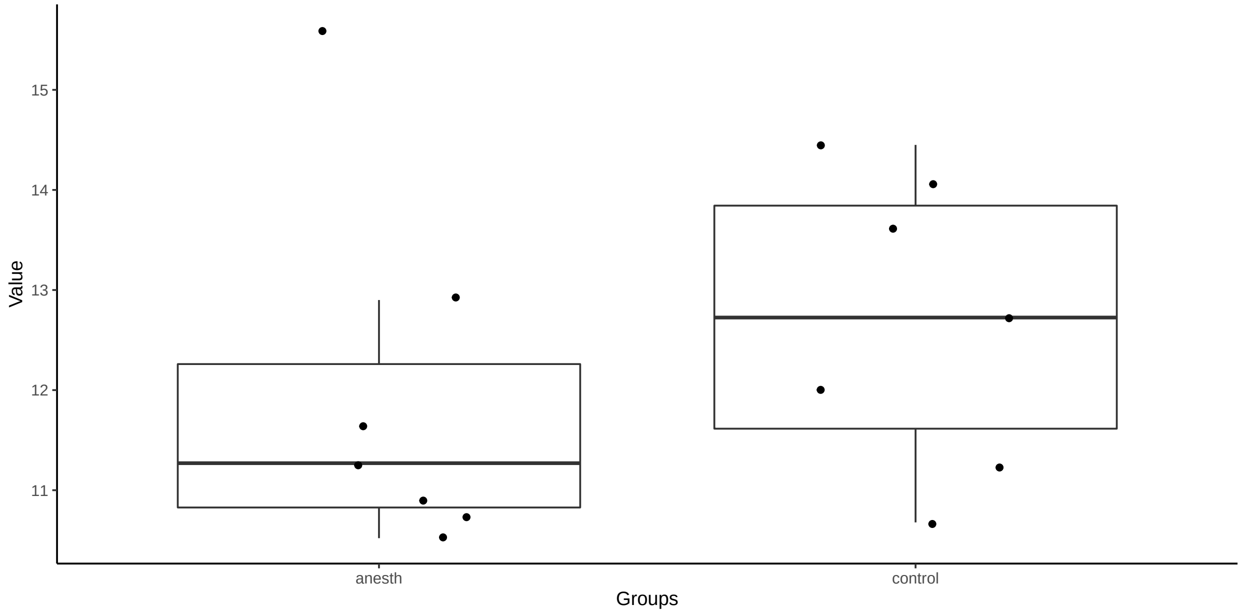


```
## 1. Normality assumption test by Shapiro_Wilk test is
##  p = 0.254
##  Normality assumption was not rejected
## 2. Equal variance test by Bartlett test is
##  p = 0.611
##  Equal variance assumption was not rejected
## 3. The result of anova is
##  p = 0.410
##  A statistically significant difference do not exist between groups
##
```
