# Supplementary material for: Repeated ketamine anesthesia during neurodevelopment upregulates hippocampal activity and enhances drug reward in male mice
Source: Commun Biol. 2022 Jul 15;5:709. doi: 10.1038/s42003-022-03667-4 (PMC9287305; doi:10.1038/s42003-022-03667-4)
Supplement: Supplementary file 3 — Description of Additional Supplementary Files [file 42003_2022_3667_MOESM3_ESM.pdf]

# Description of Additional Supplementary Files

**File name:** Supplementary Data 1
**Description:** Functional enrichment analysis of DEGs: Gene ontological term (GO) Biological Process

**File name:** Supplementary Data 2
**Description:** The source data behind the graphs in the paper
