# Supplementary material for: Repeated ketamine anesthesia during neurodevelopment upregulates hippocampal activity and enhances drug reward in male mice
Source: Commun Biol. 2022 Jul 15;5:709. doi: 10.1038/s42003-022-03667-4 (PMC9287305; doi:10.1038/s42003-022-03667-4)
Supplement: Supplementary file 6 — Reporting Summary [file 42003_2022_3667_MOESM6_ESM.pdf]

nature portfolio

Corresponding author(s): Jun Young Heo, Woosuk Chung

Last updated by author(s): Jun 15, 2022

Reporting Summary

Nature Portfolio wishes to improve the reproducibility of the work that we publish. This form provides structure for consistency and transparency in reporting. For further information on Nature Portfolio policies, see our Editorial Policies and the Editorial Policy Checklist.

Statistics

For all statistical analyses, confirm that the following items are present in the figure legend, table legend, main text, or Methods section.

n/a	Confirmed	
☐	☒	The exact sample size (*n*) for each experimental group/condition, given as a discrete number and unit of measurement
☐	☒	A statement on whether measurements were taken from distinct samples or whether the same sample was measured repeatedly
☐	☒	The statistical test(s) used AND whether they are one- or two-sided<br>*Only common tests should be described solely by name; describe more complex techniques in the Methods section.*
☐	☒	A description of all covariates tested
☐	☒	A description of any assumptions or corrections, such as tests of normality and adjustment for multiple comparisons
☐	☒	A full description of the statistical parameters including central tendency (e.g. means) or other basic estimates (e.g. regression coefficient) AND variation (e.g. standard deviation) or associated estimates of uncertainty (e.g. confidence intervals)
☐	☒	For null hypothesis testing, the test statistic (e.g. *F*, *t*, *r*) with confidence intervals, effect sizes, degrees of freedom and *P* value noted<br>*Give P values as exact values whenever suitable.*
☒	☐	For Bayesian analysis, information on the choice of priors and Markov chain Monte Carlo settings
☒	☐	For hierarchical and complex designs, identification of the appropriate level for tests and full reporting of outcomes
☒	☐	Estimates of effect sizes (e.g. Cohen's *d*, Pearson's *r*), indicating how they were calculated

Our web collection on statistics for biologists contains articles on many of the points above.

Software and code

Policy information about availability of computer code

Data collection	Clampex 11.0.3
Data analysis	Ethovision XT 11.5<br>ChemStation software (Agilent Technologies, CA, USA)<br>Clampfit 11.0.3<br>R (v.3.5.3; v.4.0.3)<br>Salmon (v1.1.0)<br>R/Bioconductor DEseq2 (v1.30.1)<br>Enrichr software

For manuscripts utilizing custom algorithms or software that are central to the research but not yet described in published literature, software must be made available to editors and reviewers. We strongly encourage code deposition in a community repository (e.g. GitHub). See the Nature Portfolio guidelines for submitting code & software for further information.

Data

Policy information about availability of data

All manuscripts must include a data availability statement. This statement should provide the following information, where applicable:

- Accession codes, unique identifiers, or web links for publicly available datasets
- A description of any restrictions on data availability
- For clinical datasets or third party data, please ensure that the statement adheres to our policy

All data generated or analyzed in the paper are present in the paper and/or the Supplementary Information. Additional data are available from corresponding authors on reasonable request.

Field-specific reporting

Please select the one below that is the best fit for your research. If you are not sure, read the appropriate sections before making your selection.

☒ Life sciences ☐ Behavioural & social sciences ☐ Ecological, evolutionary & environmental sciences

For a reference copy of the document with all sections, see nature.com/documents/nr-reporting-summary-flat.pdf

Life sciences study design

All studies must disclose on these points even when the disclosure is negative.

Sample size	Sample sizes for behavior/electrophysiology studies were based on previous studies. Detailed information for each study was described in the manuscript and supplementary data.
Data exclusions	For behavioral tests, mice with growth disturbance due to malocclusion or severe injury due to fighting in group-housed mice were excluded for behavioral tests.<br>Whole-cell recordings were included only in the following conditions: initial access resistance (Ra) < 20 MΩ; membrane resistance (Rm) > 100 MΩ; membrane capacitance (Cm) >100 pF; and < 20 % change in Ra at the end of the recording.
Replication	Behavioral experiments were performed with multiple cohorts of mice.
Randomization	Mice were randomly divided into two groups at PND 16 (Control and Anesthesia group)
Blinding	Behavioral tests were performed and video recorded by a blind experimenter. Video recordings were later automatically analyzed using a tracking software.<br>In order to avoid confusion, electrophysiology (whole-cell recordings) studies were performed by a non-blinded experimenter. However, recordings were blindly analyzed.

Reporting for specific materials, systems and methods

We require information from authors about some types of materials, experimental systems and methods used in many studies. Here, indicate whether each material, system or method listed is relevant to your study. If you are not sure if a list item applies to your research, read the appropriate section before selecting a response.

Materials & experimental systems

n/a	Involved in the study	
☒	☐	Antibodies
☒	☐	Eukaryotic cell lines
☒	☐	Palaeontology and archaeology
☐	☒	Animals and other organisms
☒	☐	Human research participants
☒	☐	Clinical data
☒	☐	Dual use research of concern

Methods

n/a	Involved in the study	
☒	☐	ChIP-seq
☒	☐	Flow cytometry
☒	☐	MRI-based neuroimaging

Animals and other organisms

Policy information about studies involving animals; ARRIVE guidelines recommended for reporting animal research

Laboratory animals	C57BL/6J mice (both sex)

Wild animals	Wild animals were not included.
Field-collected samples	No sample was collected from field
Ethics oversight	All procedures were approved by the Committees on Animal Research at Chungnam National University Hospital (Daejeon, South Korea, CNUH-018-P0006, 202009A-CNU-155)

Note that full information on the approval of the study protocol must also be provided in the manuscript.
